# Assessing Vertical Allocation of Wildfire Smoke Emissions Using Observational Constraints From Airborne Lidar in the Western U.S.

**DOI:** 10.1029/2022JD036808

**Published:** 2022-11-02

**Authors:** Xinxin Ye, Pablo E. Saide, Johnathan Hair, Marta Fenn, Taylor Shingler, Amber Soja, Emily Gargulinski, Elizabeth Wiggins

**Affiliations:** ^1^ Department of Atmospheric and Oceanic Sciences University of California Los Angeles CA USA; ^2^ Institute of the Environment and Sustainability University of California Los Angeles CA USA; ^3^ NASA Langley Research Center Hampton VA USA; ^4^ Science Systems and Applications, Inc Hampon VA USA; ^5^ National Institute of Aerospace Hampton VA USA; ^6^ NASA Langley Research Center Hampton VA USA

**Keywords:** biomass burning, inverse modeling, airborne lidar, plume rise

## Abstract

Wildfire emissions are a key contributor of carbonaceous aerosols and trace gases to the atmosphere. Induced by buoyant lifting, smoke plumes can be injected into the free troposphere and lower stratosphere, which by consequence significantly affects the magnitude and distance of their influences on air quality and radiation budget. However, the vertical allocation of emissions when smoke escapes the planetary boundary layer (PBL) and the mechanism modulating it remain unclear. We present an inverse modeling framework to estimate the wildfire emissions, with their temporal and vertical evolution being constrained by assimilating aerosol extinction profiles observed from the airborne Differential Absorption Lidar‐High Spectral Resolution Lidar during the Fire Influence on Regional to Global Environments and Air Quality field campaign. Three fire events in the western U.S., which exhibit free‐tropospheric injections are examined. The constrained smoke emissions indicate considerably larger fractions of smoke injected above the PBL (*f*_>PBL_, 80%–94%) versus the column total, compared to those estimated by the WRF‐Chem model using the default plume rise option (12%–52%). The updated emission profiles yield improvements for the simulated vertical structures of the downwind transported smoke, but limited refinement of regional smoke aerosol optical depth distributions due to the spatiotemporal coverage of flight observations. These results highlight the significance of improving vertical allocation of fire emissions on advancing the modeling and forecasting of the environmental impacts of smoke.

## Key Points

- An inverse modeling framework is developed to estimate vertical wildfire emission allocation using airborne lidar observations
- Inversions for four fire cases with free‐troposphere injections suggest larger fractions of smoke injected above the planetary boundary layer than modeled
- Constrained emission profiles lead to improved vertical allocation of transported smoke but limited refinement of regional aerosol optical depth

## 1 Introduction

Biomass burning (BB) is one of the most significant emission sources of carbon‐containing aerosols and trace gases in the atmosphere (Akagi et al., 2011). The smoke constituents greatly affect atmospheric composition, optical property, carbon storage, and air quality, imposing severe risks to public health and property (Gan et al., 2017; Lassman et al., 2017; Liu et al., 2015; Reid et al., 2016). In the western U.S., wildfires have seen increased severity and frequency over the last decades (Westerling, 2006; Williams et al., 2019) owing primarily to the warming climate and consequent fuel drying (Abatzoglou & Williams, 2016; Williams et al., 2019), thus there has been an imperative need of improving our understanding of wildfire burning dynamics and the environmental impacts.

Wildfire smoke plumes can spread over a wide range of spatiotemporal scales, which is strongly associated with plume rise, or plume injection. The determinant mechanism for the initial rapid ascent of smoke plumes lies on updrafts above fires triggered by the heat and buoyancy generated during fuel combustion (Freitas et al., 2007; Torres et al., 2020). Plume injection determines the altitudes at which the fire emissions are entrained into the atmosphere. Under favorable meteorological conditions, for example, with formations of deep convection clouds known as pyrocumulonimbus (pyroCb; Peterson et al., 2021) and cyclonic systems (Magaritz‐Ronen & Raveh‐Rubin, 2021), strong fires can release plumes into the upper troposphere (Labonne et al., 2007; Val Martin et al., 2010) and the lower stratosphere in extreme cases (Das et al., 2021; Gettelman et al., 2011; Magaritz‐Ronen & Raveh‐Rubin, 2021; Peterson et al., 2018, 2021), where the smoke can persist for much longer times and travel over widespread areas, due to the faster wind speed, weaker turbulence mixing, less efficient removal processes, and thus longer species lifetime at high altitudes than within the PBL (Ansmann et al., 2018; Dirksen et al., 2009). Therefore, the vertical distribution of fire emissions, and the consequent smoke plumes, play a crucial role in determining surface air pollutant exposure (Cheeseman et al., 2020; Wang et al., 2006), as well as the downwind radiative forcing of plume aerosols over long distances (Das et al., 2021; Magaritz‐Ronen & Raveh‐Rubin, 2021) due to the different transport, turbulence conditions, and chemical processes at different vertical levels.

Despite the significance, the plume rise process is still challenging to reproduce in atmospheric chemical transport models (Baker et al., 2016; Paugam et al., 2016; Thapa et al., 2022; Val Martin et al., 2012; Ye et al., 2021). The vertical profile of smoke emissions can be determined according to two terms: the plume injection height, and the assumption to allocate the total column emissions. For the plume injection height, a variety of methodologies have been proposed for estimating it, such as: (a) prescribed region‐dependent injection height based on observations (Davison, 2004; Lavoué et al., 2000; Liousse et al., 1996), (b) empirical‐statistical approaches adapted from formulations of stacks injections (Briggs, 1975; Pavlovic et al., 2016; Raffuse et al., 2012), (c) semi‐empirical formula using key inputs of fire radiative power (FRP), PBL height, and atmospheric stability in the free troposphere following the analogy to the convective Available Potential Energy formulations (Sofiev et al., 2012), and (d) physical‐process‐based approaches that explicitly consider buoyancy, microphysics and entrainment (Freitas et al., 2007, 2010), and fire‐energy thermodynamics (Anderson et al., 2011; Chen et al., 2019). Evaluation studies have suggested limitations of these approaches and large discrepancies when compared against remote sensing data, for example, the plume height from the Multi‐angle Imaging SpectroRadiometer and Cloud‐Aerosol LiDAR with Orthogonal Polarization (CALIOP). For example, the Briggs method shows limited applicability for wildfires (Raffuse et al., 2012; Sofiev et al., 2012). Large uncertainties and discrepancies are present in predicted plume heights by different kinds of plume rise models for an individual fire event, based on a comparison against the airborne lidar data (Ye et al., 2021). Challenges exist because plume injection behavior is dependent on both the meteorological conditions and fire characteristics that are highly dynamic and heterogeneous.

Compared to the plume injection height, the observational constraints on the vertical allocation of smoke emissions are even less understood. Many models homogeneously distribute all of the fire emissions from the ground level to a prescribed plume injection height (e.g., Davison, 2004; Lavoué et al., 2000; Liousse et al., 1996). Sofiev et al. (2013) uniformly allocate the emissions inside each plume over one third to the full plume top height. A more sophisticated method (Freitas et al., 2007) also use a uniform distribution, but it's applied only for the flaming part of the total emissions, with the partition of flaming verses smoldering emissions assumed by fuel category (see Section 2.2). The allocation of fire emissions at vertical levels, especially below vs. above the PBL height, has been highlighted as a potential source of uncertainty of smoke forecasting systems for the representation of surface pollution and lofted smoke undergoing long‐range transport (Ye et al., 2021). Therefore, in this work, we use airborne lidar data to objectively constrain the vertical allocation of fire emissions using the inverse modeling approach.

Previous evaluations of plume rise models predominately focus on plume injection heights (e.g., Raffuse et al., 2012; Sofiev et al., 2012, 2013; Thapa et al., 2022; Val Martin et al., 2012), while the vertical allocation of injected smoke constituents has not been investigated. These investigations are hindered by both the absence of accurate key input parameters driving the plume rise schemes and the lack of observations of smoke plume structures. Lidar observations are unique with their ability to resolve the vertical layering of atmospheric aerosols. CALIOP is a widely used spaceborne lidar (Winker et al., 2009), which can detect aerosol layers with high precision, but have limited spatial sampling due to the narrow swath and long revisit cycle. In contrast, airborne lidars provide measurements through specific smoke plumes of interest with exceptional temporal and spatial resolutions. The NASA Langley Research Center (LaRC) airborne Differential Absorption and High Spectral Resolution Lidar high spectral resolution lidar (DIAL‐HSRL; Hair et al., 2018) has been providing measurements suitable for characterizing the structure of aerosol layers over recent decades, which are used in many perspectives, for example, the evaluations of modeled wildfire plumes (Saide et al., 2015; Ye et al., 2021), aerosol layer structures (Fast et al., 2016), and the quantification of smoke emissions (Stockwell et al., 2022; Wiggins et al., 2021).

In this work, we present an inverse modeling framework to constrain the wildfire smoke emissions by exploiting the observations from DIAL‐HSRL deployed onboard the DC‐8 aircraft, during the recent Fire Influence on Regional to Global Environments and Air Quality (FIREX‐AQ) field campaign (https://csl.noaa.gov/projects/firex-aq/) in the summer of 2019. The inversion methodology is configured with the capability of resolving the vertical and temporal evolution of fire emissions. Simulations are implemented for three individual fires that are comprehensively sampled by the scientific flights during FIREX‐AQ and exhibited free‐tropospheric injections, based on a separate study by our team investigating the ability of the model to predict the injection behavior (i.e., within the PBL or into the free‐troposphere; Thapa et al., 2022). We investigate the plume injection allocation with a particular focus on the fraction of smoke getting injected into the free troposphere, along with the assessment of the impacts of constrained vertical emission allocations on the model representation of downwind smoke distributions. Implications for future improvement of the plume rise scheme are discussed.

## 2 Fire Events, Data, and Methods

### 2.1 Fire Events and Modeling

We focus on three fire events, including the Shady, Tucker, and Williams Flats fires, that were sampled during the western U.S. portion of the FIREX‐AQ field campaign. These western fires as compared to the Midwestern U.S. agriculture fires are selected, because they exhibit smoke plume injections into the free troposphere at least for one of the flight overpasses, based on a study thoroughly evaluating the plume injection behavior for all the fire events observed during FIREX‐AQ (Thapa et al., 2022). The specific dates and fire locations selected for analyses are shown in Table 1, along with the daily burned areas based on the Fuel2Fire data (see more details in Section 2.2). These fire events are mainly fueled by forest, grasslands, shrublands, and mixed vegetations. The processing of their emissions and fuel vegetation coverage is described in Section 2.2. We note that all fires that were sampled by FIREX‐AQ and are suitable for inversion have been included in this work. For the other fire events also exhibiting injections above the PBL, they are excluded because of insufficient flight sampling with little coverage through the plume, or complex plume structures due to mountainous terrain or significantly different advection directions below and above the PBL, which prevent the inversion methodology to be feasible.

**Table 1:** Summary of Fire Events Analyzed in This Work

Fire name	Sampling date (local time)	Fire location	Burned area (km^2^)	Flight track sampling time (UTC)		Main fuel category (>75% burned area)	Proportion of fuel category (grouped types)			Proportion of fuel category (FCCS) (version 2014)	Fraction of smoke emissions injected above PBL (*f*_>PBL_)		
				Start	End		EF	SV	GR		Freitas	ANA	ANA (adjusted PBL height)
Shady	25 July 2019	44.52°N	4.558	22:20	03:28 (+1 day)	EF	0.89	0.04	0.07	Understory litter (0.42), grassland (0.38), shrubland (0.19), other (0.01)	0.52 ± 0.01	0.87 ± 0.11	0.72 ± 0.14
		115.02°W											
Tucker	29 July 2019	41.73°N	39.77	02:14 (+1 day)	04:29 (+1 day)	GR	0.02	0.08	0.90	Shrubland (0.54), grassland (0.43), other (0.03)	0.12 ± 0.08	0.80 ± 0.04	0.80 ± 0.04
		121.24°W											
Williams Flats	3 August 2019	47.98°N	43.25	21:44	02:28 (+1 day)	EF, SV, GR	0.34	0.46	0.20	Grassland (0.43), forest (0.33), shrubland (0.14), savanna (0.08), other (0.02)	0.44 ± 0.02	0.89 ± 0.18	0.82 ± 0.23
		118.62°W											
	7 August 2019	47.98°N	72.27	23:01	03:00 (+1 day)	EF, SV	0.70	0.27	0.03	Forest (0.61), grassland (0.27), savanna (0.05), shrubland (0.04), understory litter (0.03)	0.49 ± 0.02	0.94 ± 0.09	0.85 ± 0.11
		118.62°W											

*Note.* The fuel category codes of “EF”, “SV”, and “GR” represent extratropical forecast, savanna, and grassland, respectively (see Section 2.2). Tropical forest (TF) did not fuel these fire events, so it is not listed. The fractions of smoke injected into the free troposphere (*f*_>PBL_) are presented with the mean and standard deviation for simulations with the Freitas scheme (Freitas et al., 2007, 2010), analyses constrained by the inverse modeling (ANA) and with adjusted PBL height, respectively (see Section 3.2 for more details).

The transport of gaseous and particulate species emitted from the fire sources is modeled using the Weather Research and Forecast with Chemistry model (WRF‐Chem) v3.6.1 (Grell et al., 2005; Skamarock et al., 2008). This system was used in FIREX‐AQ to inform air quality forecasting and flight planning (Ye et al., 2021), as well as in other field campaigns (Redemann et al., 2021). Instead of the forecasting mode implemented by Ye et al. (2021), we run the model retrospectively. The simulation domain is set with 4 km horizontal resolution and grid dimensions of 280 × 220 for the Williams Flats and Shady fires, and 360 × 360 for the Tucker fire. For the spin‐up time, the model is initialized 36 hr ahead of 00:00 UTC on the day of sampling over the fresh smoke plume for each case. The meteorological initial and boundary conditions are derived from the 12 km North American Model Non‐hydrostatic Multiscale Model (Janjic & Gall, 2012). The model uses a simplified aerosol‐aware microphysics scheme (Thompson & Eidhammer, 2014) to reduce computational costs but gives comparable results (Saide et al., 2016) with full‐chemistry simulations for smoke events. Specifically, two categories of aerosols are considered, that is, water and ice friendly aerosols, which are non‐reactive and only undergo wet deposition. Smoke aerosols are considered to be fully contained in the water friendly aerosols. Aerosol initial and boundary conditions are obtained from monthly climatological fields derived by Thompson and Eidhammer (2014). Note that for the Williams Flats fire on 7 August, the initial chemical conditions are updated using the simulation results of the earlier case for the same fire (3 August), to consider the impact of aged smoke plumes released on previous days. Ambient aerosol extinction and aerosol optical depth (AOD; at 550 nm) are computed based on the two categories of aerosol tracers using the relative humidity (RH)‐dependent mass extinction efficiencies to consider aerosol hygroscopic growth. Dry extinction is computed at RH of 20%, which is used to estimate PM_2.5_ concentrations by assuming the mass extinction efficiency of 3.5 m^2^ g^−1^, which is in the range of observations downwind of wildfires (Kleinman et al., 2020). Note that as the extinction in the model is diagnosed at 550 nm, it is converted to 532 nm for consistency with lidar measurements by assuming the Ångström Exponent of 1.89.

### 2.2 BB Emissions

The daily total BB smoke emissions are obtained from the Quick Fire Emission Data Set (QFED) v2.5 (Darmenov & da Silva, 2013) at 0.1° spatial grid resolution. QFED emissions are developed using FRP observations from polar‐orbiting satellites and FRP‐to‐emission coefficients adjusted to improve modeling agreement with AOD estimates. The QFED data is selected since the FRP‐based emission estimates show skillful performance in smoke forecasts intercomparison for AOD, compared with hotspot‐based emission estimates (Ye et al., 2021). As we use aerosol extinction to constrain the emissions, FRP‐based emissions are expected to provide better prior emission estimates and downwind aerosol distributions.

Given that QFED data only provides daily emissions at a spatial resolution ∼three times coarser than the model grid resolution (4 km), temporal and spatial redistributions are implemented to convert the emission per species hourly and onto the model grid. The redistributions of fire emissions are described in Section 2.2.1, followed by details about vegetation, fire size, and the default representation of plume injection in the model.

#### 2.2.1 Spatial and Temporal Redistribution of Fire Emissions

For each fire event, we use the daily burned area employed in the Fuel2Fire total carbon emission inventory (Soja et al., 2004) to spatially allocate the fire source. Fuel2Fire is developed specifically for the FIREX‐AQ fire events, and the data is publicly available on the FIREX‐AQ data archive under the analysis tab (https://www-air.larc.nasa.gov/cgi-bin/ArcView/firexaq?ANALYSIS=1#SOJA.AMBER/). Fuel2Fire data has been used to intercompare with and validate fire emissions derived from other methods (Stockwell et al., 2022; Wiggins et al., 2021). The daily burned area is derived using a combination of satellite active fire detections from the Moderate Resolution Imaging Spectroradiometer (MODIS) and the Visible Infrared Imaging Radiometer Suite. Active fire pixels from these products are selected to best match ground‐verified interagency situational reports from fire management teams, as well as Geospatial Multi‐Agency Coordination (GeoMAC) fire perimeters (Wiggins et al., 2021). The daily QFED total emissions are spatially reallocated according to the re‐mapped burned area onto the model grid. An example for the spatial re‐distribution is illustrated in Figure S1 in Supporting Information S1.

In addition to the spatial redistribution, the gridded emissions need to be converted to hourly emissions by applying diurnal factors. However, the hourly distribution represented by the commonly used temporal patterns prescribed in smoke and air quality models can often differ from actual fire behavior (Ye et al., 2021). Thus, we temporally re‐distribute the daily emissions using hourly diurnal patterns obtained also from the Fuel2Fire data, which are estimated using the FRP from geostationary satellite sensors, that is, the Advanced Baseline Instruments on both Geostationary Operational Environmental Satellites −16 and −17 (Schmidt, 2019; Schmidt et al., 2013), available at every 5 min and nominal spatial resolution of 2 km at nadir (Wiggins et al., 2021). A comparison of the emission time series for the Williams Flats fire before and after the temporal redistribution is shown in Figure S1 in Supporting Information S1.

The emission redistribution method combines the capability of polar‐orbiting and geostationary satellites and provides better information on the spatial and temporal distribution of fire emissions, compared to using the original QFED footprints and the prescribed diurnal variation factors that are generally used in air quality models (Ye et al., 2021). Note that for the fires other than the selected events within the model domain, their diurnal emissions at the model grid are obtained using the processor tool “fire_emiss” provided by the Atmospheric Chemistry Observations and Modeling Lab (ACOM) of National Center for Atmospheric Research (NCAR).

#### 2.2.2 Fuel Vegetation and Fire Size

The fuel vegetation type and instantaneous fire size are also important inputs for the plume rise scheme. For the simulations, the vegetation cover areas and their fractions for each of the four fuel vegetation types are used (Table 1), namely: tropical forest (TF), extratropical forest, savanna (SV), and grassland (GR), which are derived per horizontal model grid cell by using the “fire_emiss” tool, implementing a simplified version of the MODIS Collection 5 Land Cover Type product (Wiedinmyer et al., 2011). These four fuel types are identical with what are adopted by the QFED data, and they are grouped based on the more detailed MODIS International Geosphere‐Biosphere Program (IGBP) classifications (Darmenov & da Silva, 2013). The main fuel category is in consistency with the classification reported by Wiggins et al. (2020). For comparison, the fractions of fuel types based on the 30 m resolution Fuels Characteristics and Classification System data for 2014 (Ottmar et al., 2007) within the daily burned area, as used by the Fuel2Fire emission inventory (Soja et al., 2004), are also listed in Table 1. Note that shrubland is categorized as savanna according to the mapping of IGBP classification to the four fuel types (Darmenov & da Silva, 2013). Although the simplified fuel categories lead to less specific biomass information, these types represent the generic vegetation types for which emission factors are reported in the literature, and they are consistent with those adopted by the plume rise scheme in WRF‐Chem (Section 2.2.3). Therefore, these grouped fuel types are used in the following analysis. The instantaneous fire size is assumed to be 0.25 km^2^ per grid cell and per fuel category for which the fire emissions are active (Freitas et al., 2007).

#### 2.2.3 Plume Rise in WRF‐Chem

To parameterize plume rise which is a sub‐grid process, the online coupled 1‐D plume rise model in WRF‐Chem (Grell et al., 2011) based on the methodology provided in Freitas et al. (2007, 2010) (referred to as Freitas scheme hereinafter) is used, which is the default scheme in the WRF‐Chem model. The Freitas scheme separates emissions as smoldering and flaming. In the default implementation used here, the grid column total emissions are equally split (50% vs. 50%) between smoldering and flaming. It should be noted that this assumption is different than what is used in the original formulation (Freitas et al., 2007), where the fraction of flaming emissions is dependent on fuel type (45%, 75%, and 97% for forest, woody savanna, and grassland, respectively). For the vertical allocation, smoldering emissions are placed in the first model level, while flaming emissions are uniformly distributed between the lower and upper injection height bounds derived using the 1‐D cloud resolving model embedded in the host model (WRF‐Chem), for each fuel category with prescribed combustion heat flux lower and upper limits. For grassland, only the upper bound of heat flux is provided, and the lower bound of the injection height is fixed to the third model level (Freitas et al., 2007).

These spatiotemporal configurations of smoke emissions represent the best available a priori information of the fire source, which is used as base emissions input into the model, and the simulation results with these settings are referred to as the “Base” run accordingly in the following sections.

### 2.3 Observations

#### 2.3.1 DIAL‐HSRL

The inversion system assimilates observations collected from the airborne DIAL‐HSRL (Hair et al., 2008, 2018) onboard the National Aeronautics and Space Administration (NASA) DC‐8 aircraft during the FIREX‐AQ field campaign. The flight track transects sampling the smoke plume when overpassing the plume axis and scanning along transverse legs are selected (Table 1 and Figure S2 in Supporting Information S1). DIAL‐HSRL consists of dual nadir/zenith pointing lidar to characterize the full vertical distribution of aerosols and their optical properties along the flight track. The retrieval data includes aerosol backscatter (532 and 1,064 nm) and aerosol extinction profiles (532 nm) generated at 10 s temporal and 30 m vertical resolution.

**Figure 1:**
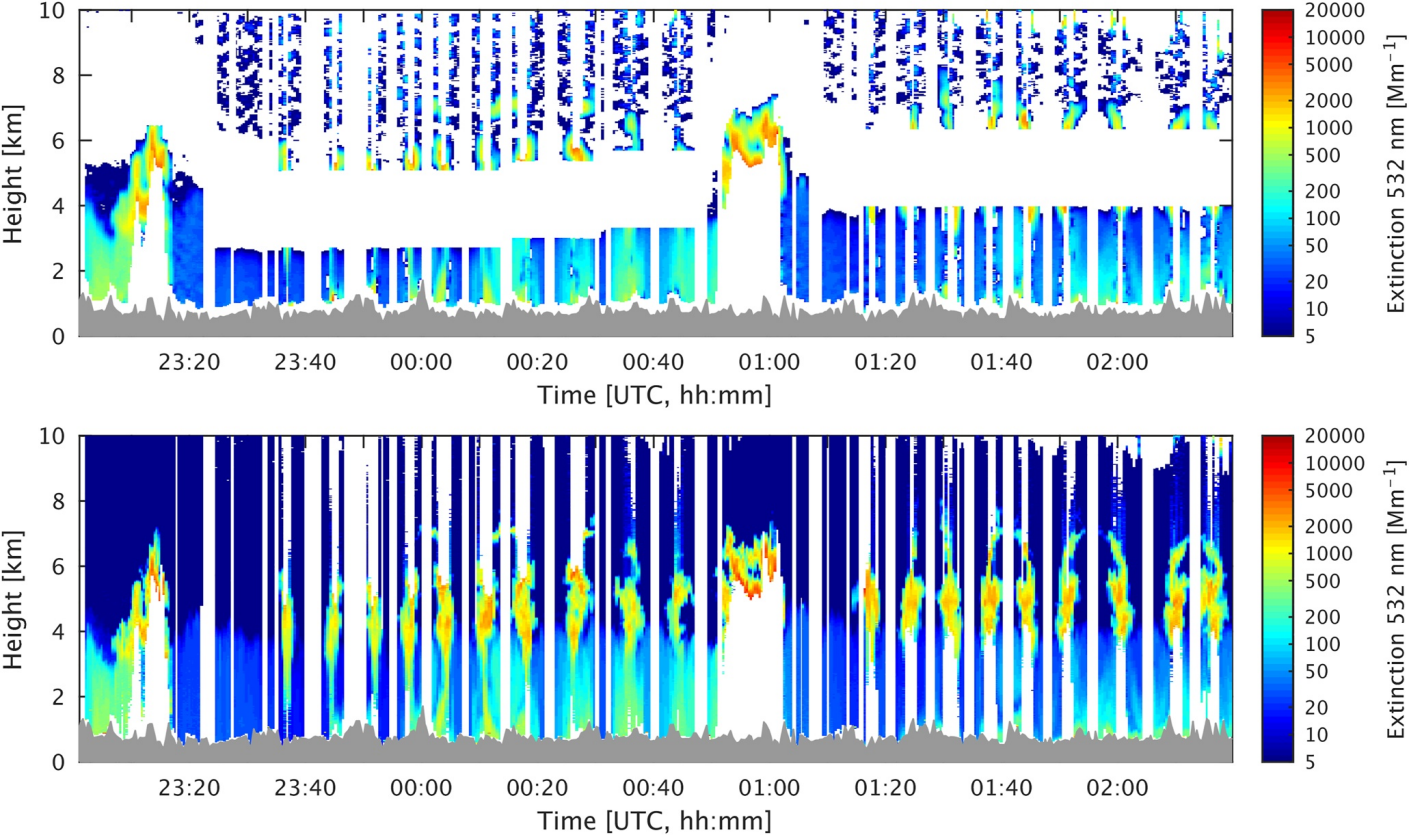
Aerosol extinction (532 nm) measured by Differential Absorption and High Spectral Resolution Lidar high spectral resolution lidar (DIAL‐HSRL; upper panel) and calculated extinction by multiplying the observed backscatter by lidar ratio (lower panel, see Section 2.3 for the method) along a portion of flight track over the Williams Flats fire on 7 August 2019.

The extinction (532 nm) profiles can directly provide constraints on fire emissions, which can characterize smoke aerosol loading through the column. However, because the DIAL‐HSRL extinction measurement requires a large standoff distance from the aircraft (0.75–1 km) in both directions due to the telescope geometrical overlap, the availability of the extinction data is limited when the aircraft flies directly within the smoke plume (Figure 1) as was the case most of the time. By contrast, the backscatter coefficient does not have this limitation. Therefore, we used the backscatter observations to estimate extinction. First, the small backscatter profile gaps (∼100 m) adjacent to the flight height are filled in by linear interpolation. Then the average lidar ratio (i.e., extinction/backscatter) is derived for each flight track segment between flight turns (e.g., Figure S3 in Supporting Information S1) using the mean extinction and backscatter profiles. The average lidar ratio weighted by aerosol extinction is used for a better representation for smoke aerosol. Lidar ratio is an intensive parameter that does not depend on the aerosol number concentration and only depends on the particle composition and microphysical properties, varying less than 10%–15% along each plume. Thus, we calculate the extinction profiles by multiplying the backscatter by the average lidar ratio. The calculated extinction results exhibit an overall good agreement with the direct extinction measurements (Figure 1), with the overall mean bias (MB) ranging from 1.50 to 9.75 Mm^−1^ and correlation coefficient of 0.83–0.91 for the flights included here (see the comparisons in Figure S4 in Supporting Information S1). To isolate the aerosol signal contributed by the fire, smoke aerosol extinction enhancement (smoke EXT532) profiles are derived by subtracting a background profile represented by the average extinction profile taken upwind of the fire location for each plume analyzed. The extracted smoke EXT532 are averaged for each set of profiles matched to the same model grid, which are then assimilated in the inversion system (Section 2.4).

In addition to the lidar measurements over the fresh plumes that are included into the inversions, flight sampling was implemented over the transported smoke plumes originating from the fire cases being constrained, which allows us to independently evaluate the modeled smoke based on the inversion results. For this purpose and depending on data availability, we include three flight track subsets for Tucker fire (21:20–22:43 UTC, 30 July) and Williams Flats fire (21:44–22:40 UTC, 8 August; 00:09–00:36 UTC, 9 August) for the evaluation of modeled smoke plumes (Section 3.3).

#### 2.3.2 MODIS MAIAC AOD

Satellite and surface in situ observations (see Section 2.3.3) are used to examine downwind air quality and validate the simulations driven by emissions based on the inversion results. The retrievals of AOD at 550 nm from MODIS using the Multi‐Angle Implementation of Atmospheric Correction (MAIAC) algorithm (MCD19A2, Version 6; Lyapustin & Wang, 2018; Lyapustin et al., 2018) provides evidence of the spatial distribution of smoke. The MAIAC algorithm uses both Terra and Aqua satellite data to provide AOD at 1 km pixel resolution (https://lpdaac.usgs.gov/products/mcd19a2v006/). Since the AOD is retrieved from visible band measurements, only daytime data are available. The AOD accuracy is reported as ± (0.05% + 10%) in a global validation (Lyapustin et al., 2018). For post‐processing, the data are filtered according to the quality assessment flags in a similar way as implemented by Ye et al. (2021), keeping the retrievals with cloud masks indicating “clear” or “possibly cloudy” and adjacency flags of “clear” or “adjacent to a single cloudy pixel”. The filtered tiles of retrievals are concatenated and remapped onto a 0.1°‐resolution grid at observation times rounded to full hours.

#### 2.3.3 Ground‐Based Measurements

AErosol RObotic NETwork (AERONET) Version 3, Level 2.0 (Giles et al., 2019) AOD retrievals at the Rimrock site allows for the evaluation of temporal evolution of smoke loading at the specific location. The AERONET AOD at 500 nm is converted to 550 nm using the Ångström exponent retrieved for 440–675 nm, for consistency with the wavelength of the MODIS MAIAC AOD data and modeled AOD. In addition, hourly surface observations of PM_2.5_ mass concentrations are used, which are collected from the AirNow (https://www.airnow.gov/) network and accessed from the OpenAQ Platform (https://openaq.org). The AirNow data has been compared with the U.S. EPA's Air Data (https://www.epa.gov/outdoor-air-quality-data) and confirmed their consistency (Ye et al., 2021).

### 2.4 Inversion Methodology

A recent study derived smoke emissions by integrating DIAL‐HSRL over the whole column (Wiggins et al., 2021). Thus, we presumed that a similar approach could be used to derive the injection fraction by vertically integrating the smoke loading below and above the mixed layer. Multiple challenges were encountered that include: (a) due to attenuation of the lidar signal, there are missing retrievals that prevent us from accurately estimating the total smoke load, (b) wind speeds in the mixed layer are generally much slower than in the free‐troposphere (due to surface roughness) and thus smoke below and above the mixed layer in an individual column does not correspond to the same time of emission, and (c) the plume age of boundary layer smoke is challenging to estimate owing to the strong turbulent and convective mixing. Thus, we developed an inverse modeling approach to derive the vertically resolved emissions which can be used to estimate the injected smoke fraction above the PBL.

**Figure 2:**
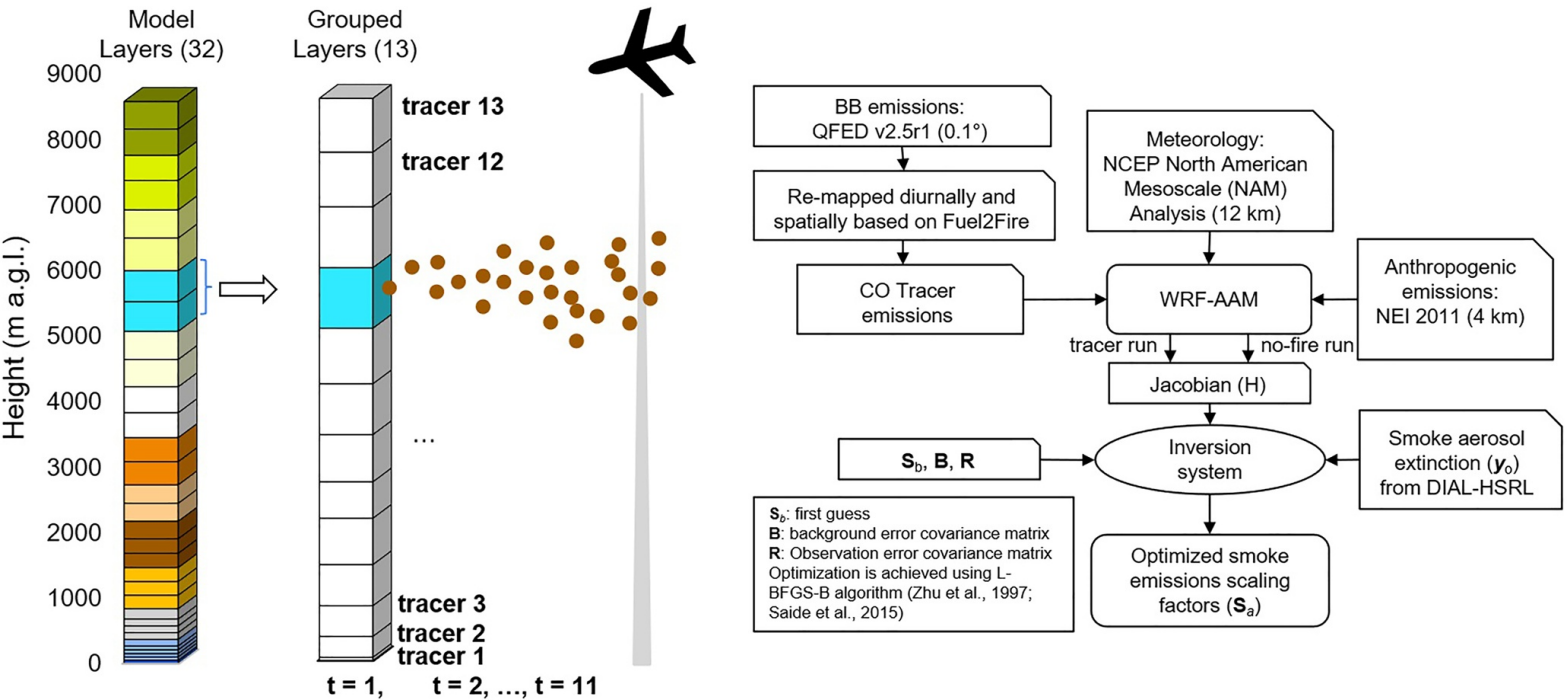
Schematic diagram (left) of the fire smoke tracers set to track emitted species in each hour and from each grouped layer. The corresponding model layer(s) belonging to each grouped layer are highlighted in different colors. The time step (*t*) represents the hour of emissions. The Flow chart (right) shows framework of the inversion system used to estimate smoke emissions by assimilating the Differential Absorption and High Spectral Resolution Lidar high spectral resolution lidar (DIAL‐HSRL) data.

To inversely estimate smoke emissions, forward simulations with the WRF‐Chem model are performed to quantitatively construct the connections between smoke emissions and downwind smoke aerosol extinction, similar to the methodology proposed by Saide et al. (2015) based on the Bayesian inversion theory (Brasseur & Jacob, 2017). Instead of the hourly passive tracers employed by Saide et al. (2015), in this work we use multiple tracers per hour to track the transport and dispersion of smoke CO released during each hour and from different vertical levels, as shown by the schematic plot in Figure 2. To reduce computational cost, rather than using the original model layers, we designate the layers of interest with grouped layers from the first to the 32nd model layers, approximately covering up to 8.5 km above the ground level. Emissions from a period of interest over 11 hr and within 13 grouped layers are tracked separately, resulting 143 tracers. Another two tracers are tagged with smoke emissions before the period of interest from the first model layer and all the layers above, respectively. One more tracer is tagged to emissions after the period of interest and from all the other fires. One hundred forty‐six tracers are used in total. The tracers are sampled at the observation time (rounded to full hours), column locations, and heights to construct the Jacobian matrix, which represents the sensitivity of downwind smoke aerosol extinction to the emissions. These emissions are not distributed vertically following the Freitas parameterization to make results independent of this approach. Instead, the total emissions from each hour during the period of interest are uniformly distributed vertically through the 13 grouped layers, which is used as the first‐guess emissions. This allows the tracking of the possible contributions from all layers of interest and allows for constraining the height of plume injection. This distribution is applied for all chemical species emitted. Examples of the first‐guess emissions and the normalized emission strength by time and height for the four cases are presented in Figure S5 in Supporting Information S1. The 11 hr period of interest is selected based on observation data availability. For the four sampling dates (Table 1), the periods of interest are 17:00 UTC 25 July to 03:00 UTC 26 July, 18:00 UTC 29 July to 04:00 UTC 30 July, 16:00 UTC 3 August to 02:00 UTC 4 August, and 17:00 UTC 7 August to 03:00 UTC 8 August, respectively. The tracer emissions before the period of interest follows the Base run with the plume rise parameterized using the Freitas scheme.

The diagram of data flow of the inversion system is shown in Figure 2. For each fire event and sampling day, four simulations are performed, including: (a) base run with the emissions and Freitas plume rise scheme as detailed in Section 2.2, (b) tracer run with emissions temporally and vertically tracked, (c) no‐fire run with fire emissions turned off, and (d) “Analysis” run with constrained emissions. The difference in results from runs (b) and (c) are used to compute the derivatives (**H**), which represents the sensitivity of the smoke plume aerosol extinctions per observation time/location to fire emissions from each vertical level and each hour. As the tracer run represents results with unit scaling factors, the finite difference derivative can be calculated as:

(1) $$H_{i}{,}_{k}=\frac{\partial y_{i}}{\partial S_{k}}=\frac{y_{i,p}-y_{i,b}}{S_{k,p}-S_{k,b}}={\left(\Delta CO_{tr}\right)}_{i,k}\cdot \alpha_{i}$$

where $H_{i}{,}_{k}$ is a component of the Jacobian matrix, *i* represents each observation time/location, *k* represents the emission tracer index ranging from 1 to *N*_f_ = 146, *y* is the aerosol extinction, *S*_k_ is the scaling factor, Δ*CO*_*tr*_ is the CO tracer concentration increment due to fire emissions. The subscripts *b* and *p* stand for the no‐fire run and the tracer run. *α*_i_ is used to convert the CO sensitivity to extinction sensitivity, which is derived as follows (assuming proportional contribution of smoke CO and aerosol extinction):

(2) $$\alpha_{i}=\frac{y_{i,p}-y_{i,b}}{\sum_{k=1}^{N_{f}}{\left(\Delta CO_{tr}\right)}_{i,k}}$$

The Jacobian matrix *H* is used in a variational inversion algorithm to optimize the scaling factors for the best fit between modeled and observed aerosol extinctions (***y***_o_). The cost‐function (*J*) is selected as:

(3) $$J\left(S\right)=\frac{EW_{f}^{-1}}{2}{\left(S-S_{b}\right)}^{T}B^{-1}\left(S-S_{b}\right)+\frac{1}{2}{\left(y_{o}-H\cdot S\right)}^{T}R^{-1}\left(y_{o}-H\cdot S\right)$$

which is different than the logarithm cost function used in Saide et al. (2015). Here, ***S*** represents the vector of scaling factors, ***y***_o_ is the observation vector of smoke aerosol extinction enhancements, **R** is the observation uncertainties, and **B** is the background uncertainties. A variational optimization (L‐BFGS‐B) algorithm (Saide et al., 2015; Zhu et al., 1997) is used to optimize the emission scaling factors of all the tracers, to achieve the best match to the observed smoke aerosol extinction by simultaneously accounting for error‐weighted information from the a priori smoke emissions and observations.

The cost function can be viewed as two terms as *J*_1_ + *E* * *J*_2_, which represent the differences between optimized scaling factors and initial guesses, and between modeled and observed extinction enhancements, respectively. *E* is a regularization parameter commonly used to balance the two terms of the cost function (Henze et al., 2009; Saide et al., 2015; Zhang et al., 2015). We used the *L*‐curve method to set the *E* value (Hansen, 1998; Saide et al., 2015), which plots *J*_1_ vs. *J*_2_ against different *E* values, and choose the best value as the one maximizing the local curvature (Figure S6 in Supporting Information S1). The *E* values chosen here are 100 (25 July 2019, Shady), 200 (29 July 2019, Tucker), 10 (3 August 2019, Williams Flats), and 20 (7 August 2019, Williams Flats), respectively. *W*_f_ is a constant defined as *W*_f_ = *N*_f_/*N*_obs_, which is the number of tracers being optimized divided by the total number of observations.

The observation uncertainty **R** is considered as a diagonal matrix, that is, no correlation between observation errors, and all the observation records have the same uncertainty, similar as used in Saide et al. (2015). **B** is computed by assuming an exponential decay by time:

(4) $$B=e^{-\frac{\Delta t}{L_{t}}}$$

where Δ*t* is the time difference between tracers, and *L*_t_ is the correlation length scale of 4 hr, selected by sensitivity tests to refine the inversion performance. The temporal correlation in the scaling factor errors help generate smoother results. Another option of using correlation between vertical levels are also tested, which does not show significant difference compared to the temporal correlations.

The method used in this work has advantages of not requiring an adjoint of the full‐chemistry model, and therefore is cost‐effective. It also allows for consideration of the 3‐D meteorology for the smoke transport, compared to the inversions with the transport described by Lagrangian models. The atmospheric transport depicted by the forward model plays an important role in the inversions. Accurate knowledge of meteorological condition is critical to interpreting and attributing the observed smoke aerosol signals to the upwind fire source correctly. However, given that one of the premises of inverse modeling is that errors are random (Brasseur & Jacob, 2017), the modeled smoke plume which is biased in location compared to observation can not be corrected by the inversions effectively, which turns out to be a particular issue when spatial shifts are present in the modeled smoke plume due to the transport model errors, for example, for the Shady fire (Section 3.1). In order to deal with this limitation and considering that the cross‐plume integrated smoke signal is less affected by the spatial displacement, two methods with respect to observables are tested, that is, (a) assimilating individual smoke EXT532 profiles and (b) assimilating accumulated smoke EXT532 profiles along the transverse flight transect legs, along with individual profiles for overpassing flight transects (Section 3.1).

## 3 Results

### 3.1 Inversion Tests for Assimilated Observable

**Figure 3:**
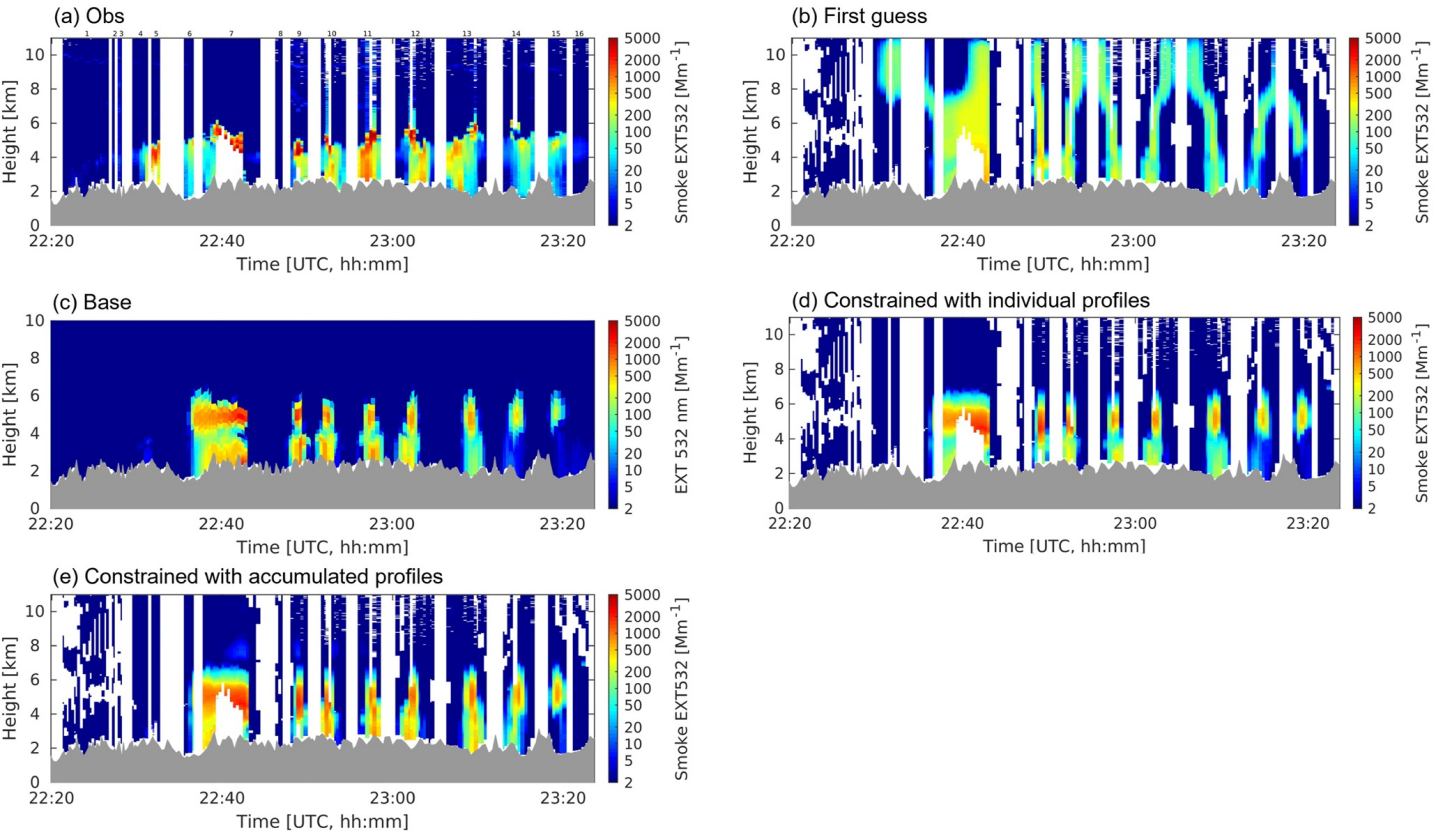
Curtain plots of smoke aerosol extinction enhancement at 532 nm for the Shady fire on 25 July 2019 from 22:20 to 23:24 UTC. (a) Differential Absorption and High Spectral Resolution Lidar high spectral resolution lidar (DIAL‐HSRL) observations; (b) simulated with first guess emissions; (c) simulation result of the Base run; (d) constrained with individual extinction profiles; (e) constrained with accumulated extinction profiles. The numbers labeled on panel (a) stand for flight transect segments between aircraft turns.

The initial experiment attempts to assimilate individual smoke EXT532 profiles, while an issue of underestimated PBL smoke enhancements is present owing to the plume location displacements. Figure 3 shows the example inversion results for the Shady fire, assimilating a subset of the selected flight transect (22:20–23:24 UTC 25 July 2019). Although the method works properly on constraining the vertical extent of injected emissions, as is evidenced by the largely improved smoke aerosol extinction distribution compared to that driven by the first‐guess emissions (Figure 3), the observed smoke within the PBL is not well captured by the analysis. For example, the strong PBL extinction enhancements existing around 23:00 UTC remains lower than the lidar measurements after the inversion (Figures 3a and 3c). This discrepancy is caused by the mismatched plume locations between the model and DIAL‐HSRL measurements with the former shifted slightly to the south (Figure S7 in Supporting Information S1). The spatial offset is more obvious within than above the PBL (Figures S7c–S7f in Supporting Information S1), suggesting that it is mostly attributed to transport errors in the lower troposphere.

In comparison with using individual profiles, assimilating transect‐accumulated extinction profiles for the transverse flight transect legs yields better performance (Figures 3d and 3e), as the accumulated signal is less biased due to the spatial plume shifts. The accumulation is implemented only for the flight transect segments crossing the plume at each sampling level (e.g., the transect segments labeled with numbers of 9–16 in Figure 3a), while for the data sampled along the plume in longitudinal direction, we use the profiles as they are. This method is used in the following inversions for all the fire cases. The time ranges of lidar observations being assimilated are listed in Table 1.

We evaluate the inversion results by comparing between the model performance with the base emissions (the Base run) and with lidar data constrained emissions (the Analysis run) regarding the assimilated observable, that is, the transect‐accumulated extinction (Table 2 and Figure S8 in Supporting Information S1). The distributions of smoke EXT532 profiles from lidar observations and simulations of the Base and Analysis runs are included in the supplement (Figures S9–S12 in Supporting Information S1). Overall, the Analysis runs show better agreement with the observed smoke extinctions, as suggested by the improved model skill statistics of the correlation coefficient (*r*), MB, and root‐mean‐square error. The remaining discrepancies can be largely attributed to the errors of transport modeling, residual background extinctions that are not fully removed by subtracting a background profile, and limited tracer vertical resolution which preclude the inversion to recognize emission vertical distribution with high vertical precision, because of the grouping of model layers for generating the tracers considering acceptable computational cost.

**Table 2:** Model Performance Statistics for the Base Run and the Analysis Run (With Constrained Emissions) Regarding the Assimilated Observable, That Is, the Cross‐Transect Accumulated Smoke Aerosol Extinction

Fire name	Sampling date (local time)	*N*	*r*		MB		RMSE	
			Base	Analysis	Base	Analysis	Base	Analysis
Shady	25 July	64,122	0.46	0.73	−67.34	−2.58	834.16	638.18
Tucker	29 July	15,698	0.64	0.89	−168.18	−84.74	846.02	541.93
Williams Flats	3 August	28,985	0.77	0.86	−238.95	−37.94	1,274.49	962.93
	7 August	37,233	0.59	0.84	−252.89	−55.01	1,223.13	810.07

*Note.* The metrics shown here are the total number of points (*N*), correlation coefficient (*r*), mean bias (MB), and root‐mean‐square error (RMSE).

### 3.2 Constrained Smoke Emissions and Free‐Troposphere Smoke Injection Fraction (*f*_>PBL_)

**Figure 4:**
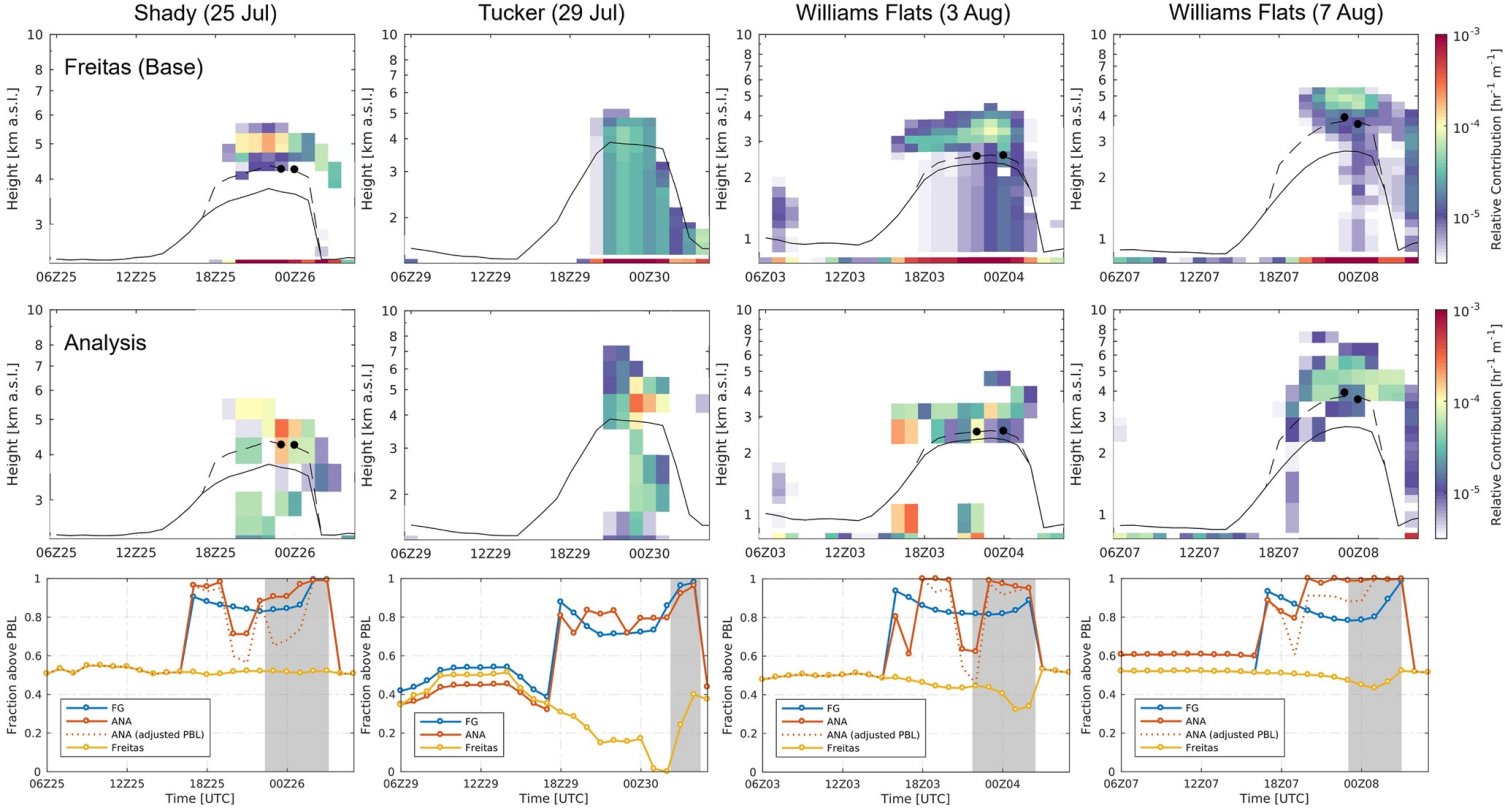
Relative biomass burning CO emission density (units: hr^−1^ km^−1^) for the Base run with plume rise determined by the Freitas scheme (top row) and for the Analysis run constrained using inversion (middle row). The black lines in emission density plots are the modeled planetary boundary layer (PBL) height derived at the fire location, and the black dots are daytime well‐mixed PBL height diagnosed using Differential Absorption and High Spectral Resolution Lidar high spectral resolution lidar (DIAL‐HSRL) backscatter profiles sampled in the upwind areas of the fire sources. The dashed lines are adjusted PBL height based on the relative bias of modeled PBL height. Adjustment is made for 11:00–18:00 PDT (UTC‐7). Note that the adjustment for Tucker fire is unavailable due to the lack of observations. Hourly fractions of emissions injected above the PBL (*f*_>PBL_) (bottom row) are shown for the first guess emissions (FG), Base run (Freitas), Analysis (ANA), and Analysis with adjusted PBL heights, respectively. The gray shading area indicates the period of DIAL‐HSRL observations being assimilated.

The vertical distributions of smoke CO emissions estimated by the Base run and constrained using lidar data are shown in Figure 4. For the Base run, 50% of column total emissions are allocated at the lowest model layer, as shown with the concentrated emissions at the surface, and the other half of smoke are evenly distributed at higher levels between the modeled lower and upper limits of plume injection height. Therefore, when the model estimated plume injection height bounds are mostly above the PBL, the vertical distribution yields a fraction of free‐troposphere smoke injections (*f*_>PBL_) around 0.5 (Figure 4), for example, for Shady and Williams Flats fires, with the means ranging from 0.44 to 0.52 (Table 1) during the hours of interest. Note that we exclude the first and last 2 hr in the period interest for the results of *f*_>PBL_ due to insufficient constraints over the entire emission profile (see Text S1 in Supporting Information S1). The temporal variability in *f*_>PBL_ for the Base run is mainly due to the relative position between the modeled PBL height and the estimated lower bound of the injection height. Noticeably, for Tucker fire, the *f*_>PBL_ is much lower, with the mean of 0.12 and more discernible variability (Table 1). The low injection fraction is related to the major fuel of grasslands, for which the lower injection limit is defined at the third model layer (see Section 2.2) and the lower limit of fire heat release is not implemented in the plume rise scheme. Therefore, the injected emissions are always assigned at some levels within the PBL (Figure 4), resulting in *f*_>PBL_ lower than 0.5.

By contrast, the constrained smoke emissions overall suggest obviously weaker emissions within the PBL without a concentrated layer at the surface, and more intense emissions exist above the PBL (Figure 4). The mean *f*_>PBL_ ranges from 0.80 to 0.94 (Table 1), much higher than for the Base run. In particular, for Tucker fire, the inversely estimated vertical emission allocation shows significant contrast below and above the PBL, rather than evenly distributing by the Base run, which implies the necessity of applying a lower limit of the fire heat flux to identify the lower limit of injection height for grasslands more realistically.

As discrepancies have been reported in different BB emission inventories (Carter et al., 2020; Kaiser et al., 2012), and uncertainties exist in the horizontal and temporal variations of smoke emissions due to limitations of satellite detection, one may wonder the impacts of prior emissions on the inversion results. We examine the effect of spatial and temporal distributions of the prior emissions by a sensitivity test implemented for Williams Flats fire on 7 August (Figure S13 in Supporting Information S1). Using QFED data processed by the “fire_emiss” tool and the redistributed emissions as described in Section 2.2.1, and with the same daily total emission magnitude, we obtain similar free‐troposphere injection fractions (0.96 ± 0.06 vs. 0.94 ± 0.09). Thus, the spatial and temporal distributions of prior emissions are not expected to significantly impact the results. Additionally, the FRP‐based inventories for Williams Flats fire differ from each other by a factor up to about 1.5 (Stockwell et al., 2022), which is much smaller than the spread of emission scaling factors retrieved from the inversion at different tracer layers (about 0–10, Figure S14 in Supporting Information S1). Therefore, we expect that using other emission inventories would not have strong influences on the inversion results.

The above *f*_>PBL_ values are based on modeled diagnosed PBL heights by the turbulence kinetic energy threshold using the Mellor‐Yamada‐Janjic scheme for PBL physics (Janjić, 2001). However, the errors in modeled thermodynamic structure and PBL heights can contribute to uncertainties in the inversely estimated injection fraction. In the recent study for all fire events sampled by FIREX‐AQ in the western U.S. (Thapa et al., 2022), the underestimated PBL heights are found to be one of the key reasons of the more frequent free‐troposphere injection cases modeled by WRF‐Chem than observed. Taking the errors in modeled PBL heights into consideration, we examine the adjusted injection fractions (*f*_>PBL,adj_) by using the corrected PBL heights based on DIAL‐HSRL data. The lidar backscatter profiles are used to identify the mixed layer heights in the daytime (e.g., Scarino et al., 2014; Tucker et al., 2009), since vertical aerosol gradients can indicate the level below which the aerosols emitted within the PBL tend to be well mixed. As the aerosols can remain in the residual layer, the identification of PBL heights is not appropriate for other conditions. Thus, the corrections of PBL heights are only implemented for 11:00–18:00 PDT (UTC‐7). We also note that the adjustment for Tucker fire is unavailable, because the lidar observation time was late and could not represent the mixed layer height.

**Figure 5:**
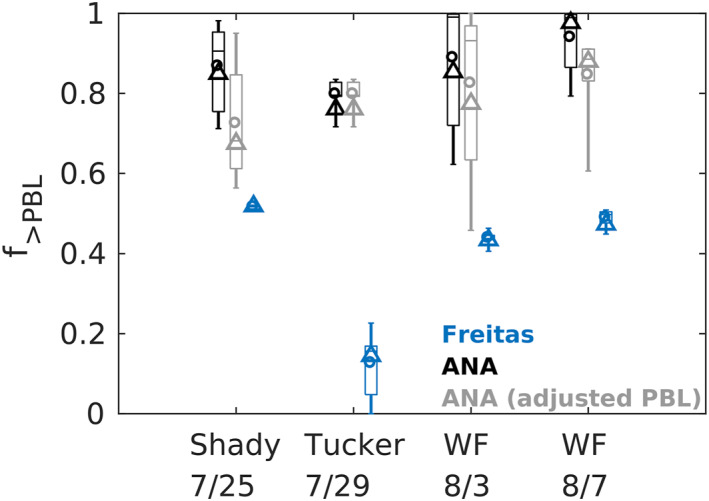
Box‐whisker plot of the free‐troposphere smoke injection fractions (*f*_>PBL_) estimated by the Freitas scheme, constrained by the inversions (ANA), and from ANA with adjusted planetary boundary layer (PBL) heights considering model errors. The edges and center line of each box stand for the 25th, 75th percentiles and the median, respectively. The lower and upper whiskers extend to the minimum and maximum, and the circle stands for the average. The triangle represents the value calculated for integrated smoke emissions during the period of interest.

The PBL heights derived from lidar data are shown as the black dots in Figure 4, using the backscatter profiles during the daytime, located upwind to the fire sources within 25 km. Overall, the model has underestimated PBL heights. We re‐calculate the fractions (*f*_>PBL,adj_) using the adjusted PBL heights, based on the relative biases in percentage compared to the observed mixed layer heights. The *f*_>PBL_ and *f*_>PBL,adj_ are summarized in Figure 5 and Table 1. The *f*_>PBL,adj_ ranges between 0.72–0.8, showing slight decreases than using the modeled PBL heights, but still much higher than for the Base run (Table 1). The largest difference is found for Shady fire with the mean injection fraction reducing from 0.87 to 0.72, because that a discernible proportion of emissions above the modeled PBL are re‐allocated within the PBL after the correction (Figure 4). Considering that the meteorological conditions are not corrected in accordance with the PBL heights, the actual *f*_>PBL_ would be slightly higher than the *f*_>PBL,adj_, because the observed free‐troposphere smoke will be attributed to emissions at higher levels if the model got the atmospheric stratification correctly. Thus, the adjusted fractions confirm the inversion results of the higher smoke injection fractions above the PBL.

The inversion results suggest that the smoke emissions are likely incorrectly allocated by the model for large and intense fire events showing injections into the free troposphere. In consequence, the loading of lofted smoke above the PBL and its contribution to air quality and radiation may be underestimated, and the surface smoke impacts may be overestimated. This issue has been reported by a multi‐model intercomparison on smoke forecasts for the Williams Flats fire, with some models presenting skillful results when evaluated against satellite AOD, but overpredicting surface PM_2.5_ concentrations over the fresh plume affected areas (Ye et al., 2021). The inversely estimated *f*_>PBL_ values for these fire events show consistency with a recent CALIOP‐based study that reports 78% of smoke detrained above the PBL (Soja et al., 2021). Therefore, improved estimation of the vertical emissions allocation, in particular, the fraction of smoke injected above the PBL, by ingesting vertical resolving information of smoke loading is recommended. However, because of the temporal and spatial coverage of the flight observations and availability for specifically designed wildfire observation missions, the information on vertical smoke aerosol distributions we can get from airborne lidar data is limited and not sufficient to constrain emissions for a longer period, especially for intense fire events featuring nighttime activity (e.g., the Williams Flats fire, see Section 3.3). Jointly assimilating airborne lidar observations and other data from multiple platforms, for example, spaceborne lidar profiles, surface species, and satellite AOD retrievals can be expected to provide more observational constraints on the complete emission profiles.

### 3.3 Impacts on Transported Smoke Distributions

Impacts of the constrained emissions on simulated downwind smoke distribution and air quality are examined with a focus on one‐day‐old, transported smoke plumes. This evaluation provides an independent verification of the inversion results as the observations involved here are not incorporated into the inverse modeling system. Due to data availability, the comparison is performed only for the Tucker and Williams Flats fires.

**Figure 6:**
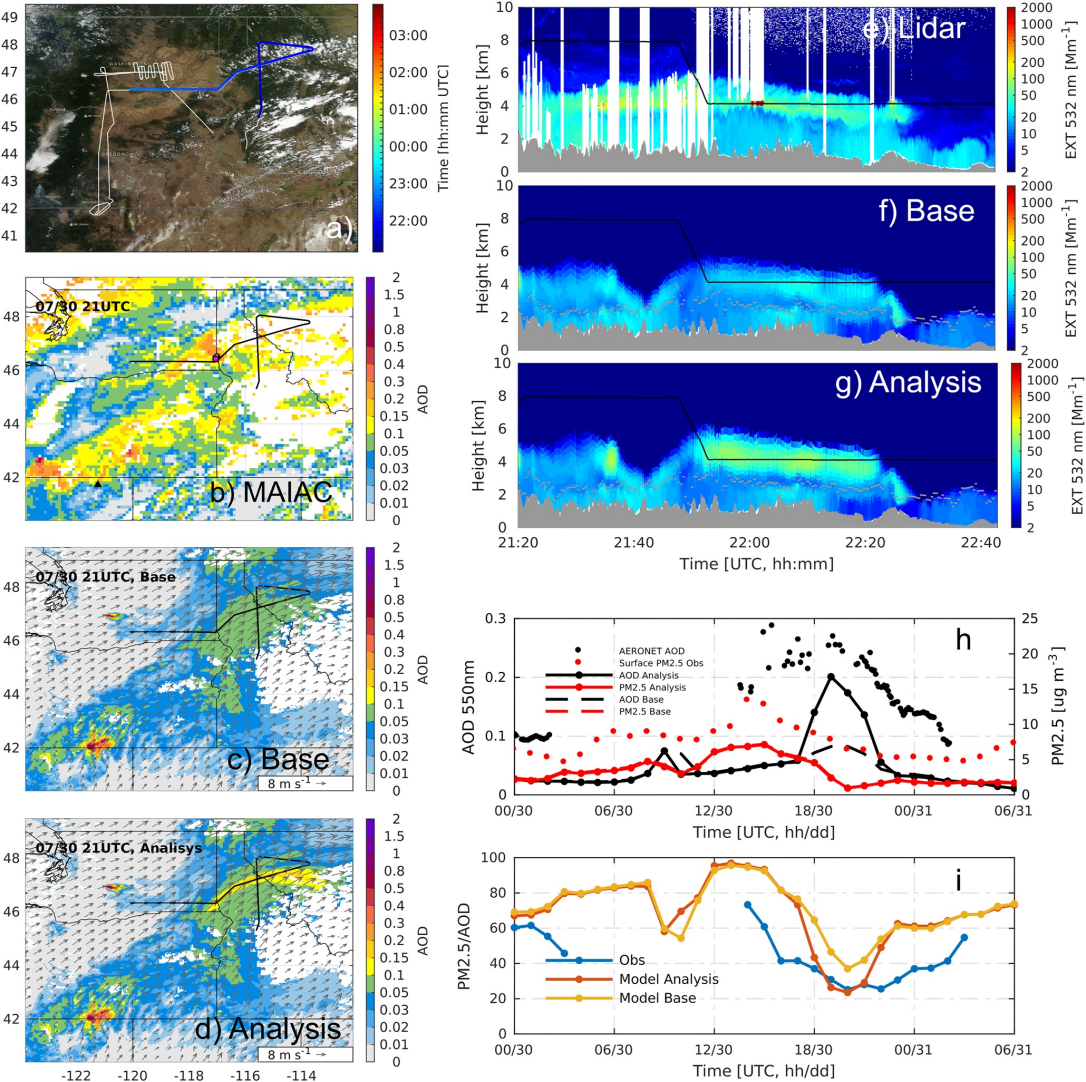
Comparison of modeled smoke from the Tucker fire against observations. Panel (a) shows the map of DC‐8 flight track at 21:20–22:43 UTC, 30 July 2019, and the selected transect is color coded by sampling time (UTC), overlayed on top of the EOS‐Aqua MODIS visible image obtained from NASA Worldview (https://worldview.earthdata.nasa.gov/). Observed smoke AOD enhancement by MODIS MAIAC data (b) and the modeled results of the (c) Base and (d) Analysis runs at 21 UTC July 30 with wind vectors at 4 km above sea level are shown for the same region. The background AOD has been subtracted. The black line shows the flight transect, the black triangle is the Tucker fire location, the white circle and the nearly overlapped magenta square denote the AERONET site Rimrock and the surface air quality monitoring site nearby. Panels (e–g) show extinction distribution by time and height observed by lidar and simulated by the Base and Analysis runs, respectively, along the colored flight track in (a). The black line shows the flight height. Panels (h and i) compare the observed and modeled results at Rimrock, including time series of AOD (black dots: AERONET; black dotted and dashed lines: modeled), surface PM_2.5_ (red dots: observation; red dotted and dashed lines: modeled), and their ratio (PM_2.5_/AOD). Note that the PM_2.5_ from Base and Analysis runs are nearly overlapped. The modeled PM_2.5_ and AOD in calculation of their ratios have been bias corrected for the time period shown in panel (i) (see Section 3.3).

Transported smoke from the Tucker fire was sampled on 30 July by the flight campaign. As shown in Figures 6e–6g, the constrained emissions yield better representation of the vertical smoke distribution along the flight track compared to the Base run, with enhanced extinction located above the PBL, corresponding to the elevated fraction of *f*_>PBL_ after the inversion. The smoke AOD enhancement (sAOD) maps present the location of the smoke plume (Figures 6b–6d). The sAOD values are derived by subtracting the background, which is represented by the average of the lowest 20% values over the whole map (Ye et al., 2021) for observations and 15% for modeling results. The smaller cutoff percentage is chosen for model to avoid excluding too much data at the borders of smoke plumes, as the background AOD is biased low than observed. The simulation with constrained emissions shows a band of plume with sAOD >0.15 extending over the north of Idaho towards Montana (Figure 6d), and it is slightly shifted to the south and narrower compared to the MODIS observations (Figure 6b). The modeled hourly AOD and wind fields (not shown) also confirm that this band of lofted smoke originates from Tucker fire. We note that although the simulation with constrained emissions shows partly improved spatial pattern of sAOD, it still does not replicate the regional sAOD distribution observed by MODIS MAIAC data. This is partly due to the misrepresented aerosol inflow from outside of the domain, that is, the smoke from the Siberia fires as shown on the northwest corner of Figure 6b, because the model used aerosol boundary conditions from monthly climatology. Moreover, the limited temporal coverage of lidar sampling has prevented the inversion from constraining the emissions before and after the period of interest, especially for the nighttime fire emissions. Third, there are other fires remaining unconstrained and underestimated by the QFED data, for instance the fire plume from the southwest of Oregon. Additionally, as aerosol chemistry is not included in this modeling, the secondary production of aerosols is not considered. Therefore, some of the observed AOD enhancements being not captured are likely associated with secondary aerosols from smoke and anthropogenic air pollutants. Regional improvements of the sAOD distributions would be more critically dependent on assimilations of satellite and surface chemical species observations over larger spatial and temporal coverages and proper boundary smoke conditions.

Besides the aerosol extinction profiles that directly measure vertical aerosol distributions, the ratio between surface PM_2.5_ and AOD (PM_2.5_/AOD) can also be used to as a useful indicator, given that a smoke plume injected higher into the free troposphere tends to have enhanced AOD but low surface PM_2.5_ over downwind area, leading to small PM_2.5_/AOD ratio and vice versa (Cheeseman et al., 2020; Ye et al., 2021). The PM_2.5_/AOD has been reported to generally decrease with increasing ratio of plume height vs. PBL height (Cheeseman et al., 2020), indicating the linkage between surface‐level and total‐column aerosols and plume injection behavior during wildfire events. Therefore, the PM_2.5_/AOD values help recognize surface smoke and lofted smoke. As shown by the modeled maps in Figure S15 in Supporting Information S1, the regions impacted by the lofted smoke plume from the Tucker fire are overall discernible with the lower PM_2.5_/AOD, in contrast to the surrounding areas. In addition, the Analysis run shows smaller ratios over the region with sAOD >0.15 than the Base run (Figure S15 in Supporting Information S1 and Figure 6d), associated with the larger proportion of smoke being injected above the PBL with inversion on the previous day.

For specific surface sites, the temporal evolution of PM_2.5_, AOD, and their ratio are examined using observations at an AERONET station (Rimrock) and a nearby surface monitoring location (with the distance of 8.65 km). Although the modeled results show biases due to using climatological aerosol concentrations as boundary conditions, as well as the unconstrained emissions owing to the reasons noted previously, the temporal variations of the surface PM_2.5_ and AOD are better captured by the Analysis (Figure 6h). We estimate the model biases according to the average model‐observation difference for the hours out of the impact of smoke (AOD bias = −0.08 and PM_2.5_ bias = −5.0 μg m^−3^). The bias‐corrected modeled PM_2.5_/AOD ratio presents better agreements with the observations (Figure 6i), suggesting the improved representation of the lofted smoke plume from Tucker fire above the PBL. This is mostly associated with the increased AOD of the Analysis, contributed by the enlarged fraction of smoke getting injected above the PBL based on the inversion. However, owing to the limited temporal and spatial coverage of the flight observations, the improvement on the ratio is shown to cover only about 3–4 hr. The peak of AERONET AOD is wider (Figure 6h), and the observed PM_2.5_ to AOD ratio remains low for a longer time period than shown by the Analysis run. Thus, more observational constraints from multiple platforms are necessary to provide a longer temporal coverage of the constraints of fire emissions and AOD distributions on a regional scale.

**Figure 7:**
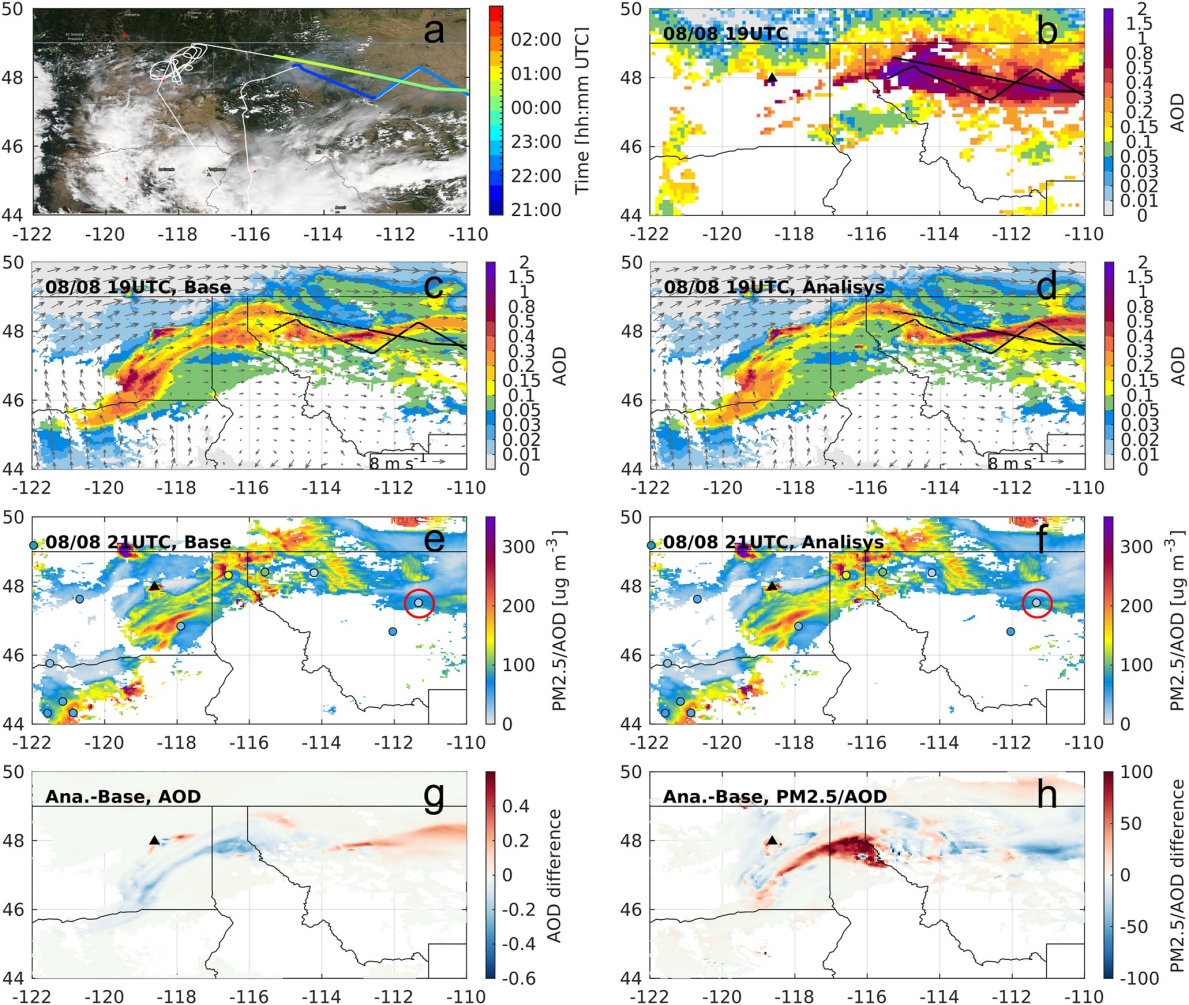
Comparison of modeled smoke from the Williams Flats fire on 8 August 2019 against observations. (a) Map of the flight track, similar as Figure 6a but showing Aqua‐MODIS visible map at around 21:00 UTC 8 August. Note that temporal offset exists between the flight sampling time and the satellite overpass; (b) Observed smoke aerosol optical depth (AOD) using MODIS MAIAC data; (c–d) Modeled smoke AOD from the Base and Analysis runs; (e–f) Modeled ratios (surface PM_2.5_/AOD) for the Base and Analysis runs, overlayed by the observed ratios (colored circles) derived from surface monitoring data and MODIS MAIAC AOD at the same hour of each panel; (g) difference of AOD between the Analysis and Base runs; (h) difference of PM_2.5_/AOD. The black triangle in (b–f) represents the fire location. The red circle in (e and f) denotes location of the surface monitor at Cascade, Montana.

Constrained emissions for the Williams Flats fire on 7 August contribute to increased loading of lofted smoke above the PBL on the following day, as suggested by the enlarged smoke AOD enhancement along a latitudinal band over the north of Montana (east of −114°E, Figures 7c and 7d), showing better representation of the transported smoke aerosols. The transported smoke plume band on the modeled map shows a slight displacement to the north compared to the satellite observations (Figure 7b), which can be due to the errors in modeled meteorology and the timing of free‐troposphere injection. For the magnitude of smoke AOD, although the Analysis result is still smaller than observed, the relative loading of surface smoke and total column smoke is improved, given the decreased PM_2.5_/AOD ratio for the Analysis run over the band of transported smoke (Figure 7h). This is confirmed by using satellite AOD and surface PM_2.5_ observations at Cascade, Montana (shown with the red circle in Figures 7e and 7f). We select this site based on the availability of surface PM2.5 observations, and collocation with the transported smoke getting affected by the inversion of emissions. The modeled PM_2.5_/AOD is 78.3 for the Analysis run and 102.2 for the Base run, with the former being closer to observed result (17.2), while it is still overestimated due to the northward spatial displacement of the modeled plume leading to the thinner plume over the Cascade site than observed. Meanwhile, the issue of limited temporal coverage of observational constraints is also present, similar to the Tucker fire case. This can be seen from the discrepancies between modeled and observed sAOD distributions, especially the underestimated aerosol loading over northwest Montana, which is not improved by the inversion (Figures 7b and 7d) and largely related to the underestimated amount of injected smoke before and after the emission period getting constrained by the lidar data. We note that for this event the initial and boundary conditions from a larger domain are not expected to contribute much to the model performance for sAOD (Ye et al., 2021).

The evaluation along flight transects on 8 August 2019 over the transported smoke from the Williams Flats fire (highlighted in Figure 7a) exhibits consistent results with the evaluation against satellite data. The comparison also shows an underrepresentation of the lofted smoke over the northwest Montana (see Figure S16 in Supporting Information S1, about 21:50–22:10 UTC). In addition, the mismatch of lofted smoke on Figure S16 in Supporting Information S1 at about 22:10–22:40 UTC corresponds to spatial shifts of the modeled smoke to the north. It's also noteworthy that, the lofted smoke plumes in model for both the Analysis and Base runs are lower than the lidar observations, and they are more clearly detached from the PBL (Figure S16 in Supporting Information S1). This is likely because of the underprediction of modeled PBL height and plume placement offset, therefore the flight track might be sampling through different portions of the plume for model and lidar data. Further investigations are needed to isolate the impacts from various relevant factors, including plume rise, transport, and aerosol processes, on the capability of modeling transported smoke distributions.

## 4 Discussions

### 4.1 Limitations

As the inversion method developed in this work relies on observations with capability of characterizing the full vertical distributions of smoke aerosol, this method is subject to limitations on scalability. Applying this approach by leveraging other measurements would be challenging, because intensive observations with good coverage through the plume is needed to make it work. Ground‐based lidar could be a candidate if the site location is adjacent to fire source. However, the effectiveness of ingesting spaceborne LIDAR data would be doubted, due to the long revisit cycle and sparseness of cases capturing fresh smoke plumes.

We recognize that because of the sporadic nature of wildfire events, continuous or regular airborne lidar observations are limited by resources and the associated costs and therefore currently impractical. Nevertheless, inversion framework similar to the one demonstrated in this work is applicable for jointly assimilating observations from different platforms (e.g., satellite‐observed AOD, ground‐based lidar data), which could be capable of constraining fire emissions over a longer time period.

Relating the free‐troposphere injection fraction to fire and atmospheric conditions are helpful for further parameterization and prediction purposes. A tentative analysis is conducted to look for possible linkages between the injection fraction and some possible explanatory parameters (see Text S2 in Supporting Information S1 and Figure S17 in Supporting Information S1). However, as the number of cases is limited, it is not sufficient to make conclusive statements. In addition, the injected fraction may vary from fire to fire depending on many combustion and atmosphere situations. In this work, we have included all the fire cases that were sampled in FIREX‐AQ and are suitable for applying the inversion method (Section 2.1). Given the complexity, additional field campaigns are needed to increase the data pool for statistics, and to provide better representativeness for various fire intensity/size, fuels, and atmospheric conditions, thus lead to more objective findings on potential explanatory variables to predict the free‐troposphere plume injection fraction.

### 4.2 Implications for Plume Rise Parameterization in Regional Models

Despite the limited scalability, the inversely constrained emission allocation below and above the PBL can advise into the selection of parameters used in the plume rise model. In the default setting of the Freitas scheme in WRF‐Chem, as well as in most regional and global chemical transport models, the vertical allocation of fire emission relies on a prescribed constant ratio and has yet to be objectively constrained using observations. In WRF‐Chem, it is assumed that 50% of emissions are emitted at the lowest model level, and the other 50% are distributed evenly within the parameterized lower and upper limits of plume injection height. This assumption is applied for any fire conditions, regardless of fuel and fire intensity. In contrast, our results using airborne lidar observations suggest that the vertical distribution of fire emissions can be largely different than the arbitrary assumptions used previously. For the fire cases examined here, the fraction of smoke getting released above the PBL is greatly underestimated by the default Freitas scheme. This means that, for similar cases when the Freitas scheme correctly estimates the plume injection behavior (into the free troposphere), increased fraction of injected smoke to about 80% will be necessary. Meanwhile, a lower limit of fire heat flux needs to be implemented for the fuel of grassland to realistically represent the injection height and emission allocation.

However, it should also be noted that the Freitas scheme generally tends to overestimate the overall occurrence frequency of free‐troposphere injections. Among all the western U.S. fires sampled in FIREX‐AQ, there is a larger fraction of cases for which the Freitas scheme estimated the plume injection heights into free troposphere, but the lidar observations suggested plumes within the PBL, likely associated with the overestimated fire heat flux and underestimated PBL heights (Thapa et al., 2022). For those cases when the plume rise model mistakenly injects smoke above the PBL, solely increasing the fraction of emissions above the PBL would make the simulation results even worse, and improved representation of the plume injection heights are important. Therefore, the enlarged free‐troposphere injection fraction needs to be implemented together with other improvements, and it tends to be applicable for fire events if the observations undoubtedly detect smoke injections above the PBL.

## 5 Conclusions

Observational constraints by airborne DIAL‐HSRL aerosol extinction profiles are ingested into an inverse modeling system to estimate the vertical allocations of fire smoke emissions associated with plume rise processes. Three fire events in the western U.S. are analyzed, which are comprehensively sampled during the FIREX‐AQ field campaign and have notable features of plume injection above the PBL. Our results suggest that the Freitas plume rise scheme implemented in the WRF‐Chem model is substantially underestimating the fraction of smoke species getting injected into the free troposphere with respect to the column total for the fire cases analyzed in this work. The fraction of free‐troposphere smoke injection (*f*_>PBL_) ranges between 80% and 94%, which would become slightly lower (72%–85%) if we consider the underestimation of modeled PBL heights. The constrained emission profiles lead to improved model representations of the vertical allocation of downwind transported smoke plume, especially the relative loading of surface and total column smoke, as suggested by the evaluation against independent observations including the satellite AOD, surface in situ PM_2.5_, and airborne DIAL‐HSRL data on the next day.

The results highlight that objectively constrained free‐troposphere plume injection behavior is important for advancing the modeling and forecasts of wildfire smoke impacts. Based on the cases investigated here, an increased fraction of smoke released above the PBL of ∼80% is suggested for fire events with well detected free‐tropospheric injection. Meanwhile, a lower limit of the fire combustion heat flux for the fuel type of grassland is suggested to be implemented to better represent the potential range of injection height. However, this increased *f*_>PBL_ is not appropriate for any fire events. Given the tendency of the Freitas scheme to overpredict the occurrence frequency of free‐tropospheric injection (Thapa et al., 2022), for the “false alarm” cases, simply increasing the fraction would worsen the simulation results. Therefore, the current recommendation for increased *f*_>PBL_ is only suitable for well detected plume injections above the PBL. With respect to broader cases, it needs to be applied together with improved fire heat flux (Thapa et al., 2022) and thermodynamic structure of the atmosphere.

Despite the improvements of vertical plume structures led to by the inversion, discrepancies between the simulations and observations are still present in terms of the smoke AOD enhancement distributions, suggesting limited impacts of the revised emissions allocation on the smoke transport/dispersion on a regional scale. This is mainly due to the limited spatiotemporal coverage of airborne lidar data, background/boundary smoke conditions, and other fires remaining misrepresented. Other reasons of the discrepancies could be the processes not accounted for in the model, for example, the aging of smoke aerosols, downwind formation and transformation of secondary organic aerosols, and aerosol properties used to calculate extinction from aerosol mass concentration. Joint assimilation of multiple categories of observations from satellite and ground‐based sensors are expected to provide more constraints on smoke distributions on regional scale.

Variability of free‐troposphere smoke injection fractions among cases can be generally contributed by the burning features (e.g., fire intensity, combustion phase, fuel amount and structure, burned area and geometry) and atmospheric conditions (e.g., stability, wind speed, and moisture distribution). However, due to the limited sample size in this work, more broad statements on the relation between these possible controlling factors and the free‐tropospheric injection fraction are not possible. Therefore, future airborne observations are required to examine the vertical smoke emission allocations over various situations. Extended investigations for more fire events based on existing and future field campaigns are warranted to explore the explanatory parameters of the free‐tropospheric injection behavior and improve predictive potential.

## Supporting information

Supporting Information S1

## Data Availability

DIAL‐HSRL data and Fuel2Fire total carbon emissions and satellite fire detections during the FIREX‐AQ mission are archived by NASA/LARC/SD/ASDC (https://doi.org/10.5067/SUBORBITAL/FIREXAQ2019/DATA001, https://www-air.larc.nasa.gov/missions/firex-aq/). QFED data are available at the NASA NCCS data portal (https://portal.nccs.nasa.gov/datashare/iesa/aerosol/emissions/QFED/v2.5r1/0.1/QFED/). AERONET AOD observations can be accessed on the AERONET website (https://aeronet.gsfc.nasa.gov/). MODIS MAIAC AOD data (MCD19A2 Version 6) are available online (https://lpdaac.usgs.gov/products/mcd19a2v006/). Surface PM_2.5_ observations are available at OpenAQ (https://openaq.org) and U.S. EPA's Air Data (https://www.epa.gov/outdoor-air-quality-data). WRF‐Chem is an open‐source community model, and the source code of WRF‐Chem v3.6.1 is available at http://www2.mmm.ucar.edu/wrf/users/download/get_source.html.
