# Supplementary material for: Assessing Vertical Allocation of Wildfire Smoke Emissions Using Observational Constraints From Airborne Lidar in the Western U.S
Source: J Geophys Res Atmos. 2022 Nov 2;127(21):e2022JD036808. doi: 10.1029/2022JD036808 (PMC10078447; doi:10.1029/2022JD036808)
Supplement: Supplementary file 1 — Supporting Information S1 [file JGRD-127-0-s001.docx]

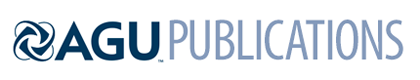


*Journal of Geophysical Research: Atmospheres*

Supporting Information for

**Assessing Vertical Allocation of Wildfire Smoke Emissions using Observational Constraints from Airborne Lidar in the Western U.S.**

Xinxin Ye^1^, Pablo E. Saide^1,2^, Johnathan Hair^3^, Marta Fenn^3,4^, Taylor Shingler^3^, Amber Soja^5,6^, Emily Gargulinski^6^, Elizabeth Wiggins^6^

1 Department of Atmospheric and Oceanic Sciences, University of California, Los Angeles, Los Angeles, CA, USA

2 Institute of the Environment and Sustainability, University of California, Los Angeles, Los Angeles, CA, USA

3 NASA Langley Research Center, Hampton, VA, USA

4 Science Systems and Applications, Inc., Hampon, VA, USA

5 National Institute of Aerospace, Hampton, VA, USA

6 NASA Langley Research Center, Hampton, VA, USA

**Contents of this file**

Text S1 to S2

Figures S1 to S19

**S1**. Sensitivity of downwind smoke to fire emissions

At a certain lidar profiling location, the sensitivity of fire smoke to tracer emissions released from different hours and levels would be different, closely depending on the atmospheric transport. We demonstrate the smoke CO concentration along a flight track in Fig. S18. The smoke CO due to emissions from the hour of 18:00 UTC is spread within the PBL, meaning that the observations would be mostly useful for constraining emissions within the PBL. This is expected, because the tracer smoke above the PBL had been transported very quickly and moved out of the sampling area of the transverse flight segments. While, for 23:00 UTC emissions, at the sampled profile locations, contributions exist for emissions both within and above the PBL.

Considering the whole flight track, we want to identify the hours of tracer emissions to which the sensitivity of downwind smoke is available both below and above the PBL, meaning that the full emission profile can be constrained by using lidar data. In other words, the observations have full representativeness for the emission profile, and the emission profile can be constrained with complete vertical coverage. Thus, we examined the flight-accumulated sensitivity of CO tracer concentrations to emissions of different hours. As seen in Fig. S19, for emissions of 16:00 UTC 3 August to 02:00 UTC 4 August, the accumulated sensitivity to the beginning 2 hours of emissions tends to be low above 5 km. This means that the downwind smoke originating from those hours cannot be fully captured by the flight transects, since the free-troposphere smoke has been transported further than the scope of the observations. For the last 2 hours, low sensitivity can be seen for 01:00 UTC below ~3 km, and for 02:00 UTC at all vertical levels. This implies that the representativeness of lidar observations is low for the smoke emitted in the hour of 01:00 UTC within the PBL and 02:00 UTC from all levels, because those smoke had not reached the flight sampling locations yet. This incomplete representativeness of emissions could lead to bias in the inversion estimates of vertical emission distributions. Therefore, the first and last two hours of the period of interest are not considered when we analyze the free-troposphere smoke injection fraction.

**S2**. Possible Factors Modulating f_>PBL_

The inversion analyses presented in section 3.2 indicate fire-to-fire and day-to-day variability in the injection fraction (f_>PBL_). The smoke injection behavior is closely relevant to fuels, combustion energy, and atmospheric environment conditions. Here several explanatory parameters are examined to investigate their relation to f>_PBL_, aiming at looking for possible indicators that could be used to empirically predict free-troposphere smoke injection fraction with fire and environmental conditions.

Multiple factors are considered, including: 1) hourly average FRP derived from GOES-17 retrievals, 2) Fire radiative energy (FRE) flux, which is calculated by dividing the total FRP by fire size, both of which are derived by the MODIS-Advanced Spaceborne Thermal Emission and Reflection Radiometer (ASTER) airborne simulator (MASTER) data (Hook et al., 2001; Thapa et al., 2022), 3) plume injection height (h_Inj_) estimated using DIAL-HSRL aerosol extinction profiles sampled within 30 km downwind to the fire location, 4) biomass burning CO emissions from the re-distributed emissions based on QFED data (see section 2.2.1 in the main text), 5) burned area and fuel type, 6) PBL height (h_PBL_) derived from model diagnosis, 7) difference between the medians of injection and PBL height (h_Inj_ – h_PBL_), 8) Brunt–Väisälä frequency and 9) average wind speed within the layer between PBL and 1.5×PBL height, using the modeled temperature and wind profiles, and 10) pyroCb firepower threshold (PFT) (Tory and Kepert, 2021).

Here we describe more about the PFT, which is a recently reported parameter to provide insight into the joint importance of atmospheric state and fire heat release for plume rise behavior and development of deep convection. More details about the derivation of PFT can be found in Tory and Kepert (2021). Briefly, PFT physically refers to the minimum fire heat flux required to generate deep, moist convection (pyroCb) in any given atmospheric environment. It can be used to diagnose the favorability of the atmospheric condition for pyroCb formation. We calculate PFT using the analytical solution in Tory and Kepert (2021). The PFT is proportional to the product of mixed-layer wind speed, temperature increment due to fire burning (Δθ), and the square of a minimum cloud-base height (z_fc_, free convection height). The smaller PFT values favor pyroCb development; on the other hand, the taller z_fc_, stronger horizontal wind, and greater capping inversion that the plume must penetrate mean the higher fire energy required, thus the higher PFT.

The above factors that could be modulating f_>PBL_ are extracted during the time period of interest (except for the FRE flux) for each sampling day. Note that the beginning and last two hours are not included, as the observational representativeness of lidar data for fire emissions of those hours is limited (see Text S1). Although taking this into consideration, the temporal range of constrained emissions still covers the hours of high daytime burning activity.

The results for each fire case are presented by the box-whisker plots in Fig. S17. Overall, Tucker fire shows the lowest f_>PBL_ (~0.80) and moderate f_>PBL,adj_ with adjustment of PBL height. As a fast-moving fire with moderate fire size and mainly fueled by grasslands, the injection is largely favored by the high convection potential (low PFT).

In comparison, the other three cases are mainly fueled by forest and savanna (shrublands before lumped) (Fig. S17). For those three cases, the f_>PBL_ values present positive correlations with FRP, fire size, h_Inj_, and PFT. In particular, the case of Williams Flats fire on 7 August shows the largest f_>PBL_ (~0.94), which is characterized with favorable conditions for the above four factors and significantly larger h_Inj_-h_PBL_. Besides, the upper-level stability is less stable for the Williams Flats fire, as shown by the lower Brunt–Väisälä frequency.

The variability of the free-troposphere injection fraction from fire to fire is relevant to the fire and atmospheric environment conditions. Due to the limited sample size, there is no discernable correlation between the f_>PBL_ and an individual explanatory factor analyzed here. Analyses of more field campaign observations and future missions are warranted to enlarge the data pool and provide statistical statements on the dependency of the free-troposphere injection behavior to the various explanatory variables.

**References**

Hook, S. J., Myers, J. J., Thome, K. J., Fitzgerald, M., & Kahle, A. B. (2001). The MODIS/ASTER airborne simulator (MASTER) — a new instrument for earth science studies. *Remote Sensing of Environment*, 76(1), 93–102. <https://doi.org/10.1016/S0034-4257(00)00195-4>

Thapa, L. H., Ye, X., Hair, J. W., Fenn, M. A., Shingler, T., Kondragunta, S., et al. (2022). Heat flux assumptions contribute to overestimation of wildfire smoke injection into the free troposphere. *Communications Earth & Environment*, *3*(1), 1–11. <https://doi.org/10.1038/s43247-022-00563-x>

Tory, K. J., & Kepert, J. D. (2021). Pyrocumulonimbus Firepower Threshold: Assessing the atmospheric potential for pyroCb. *Weather and Forecasting*, 36(2), 439-456.


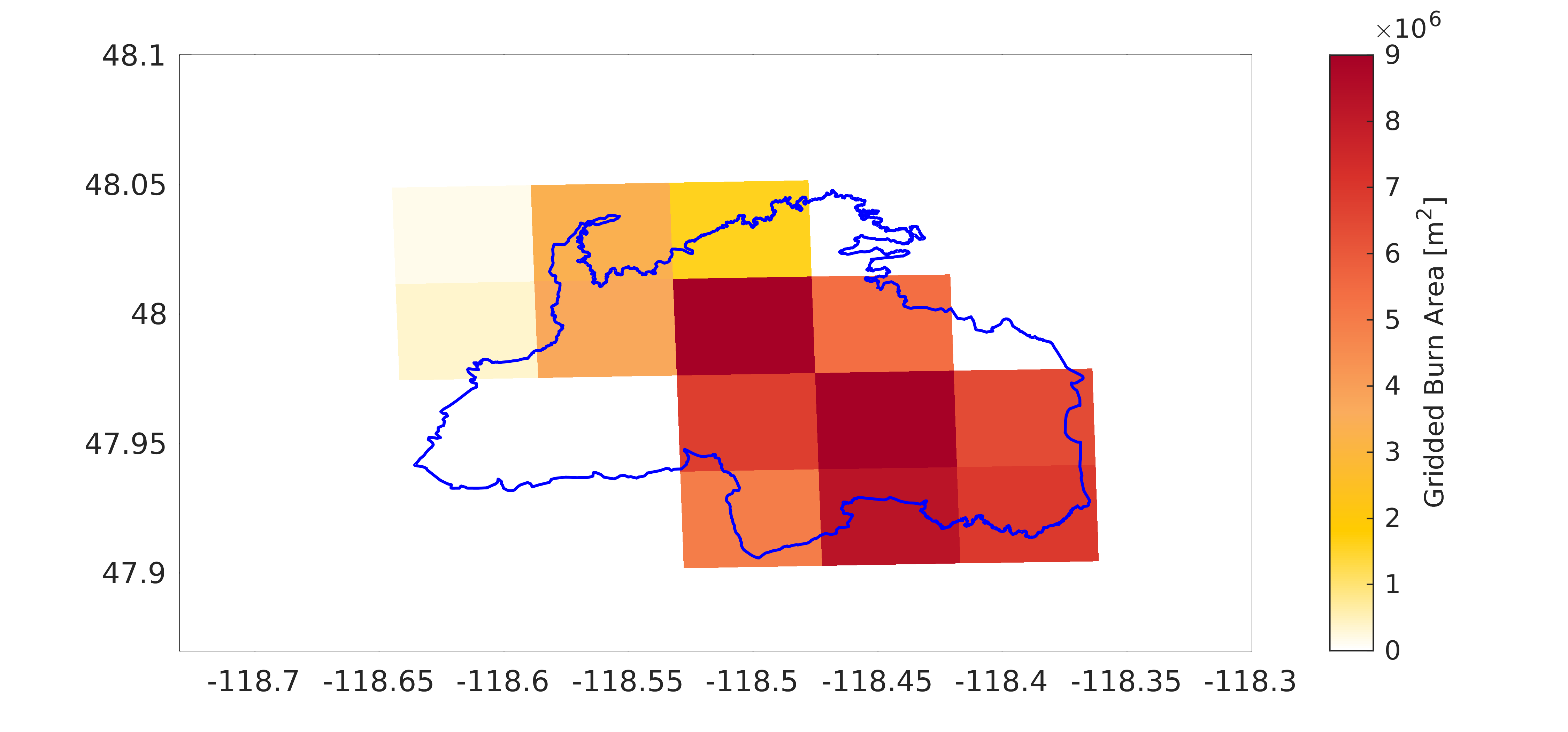

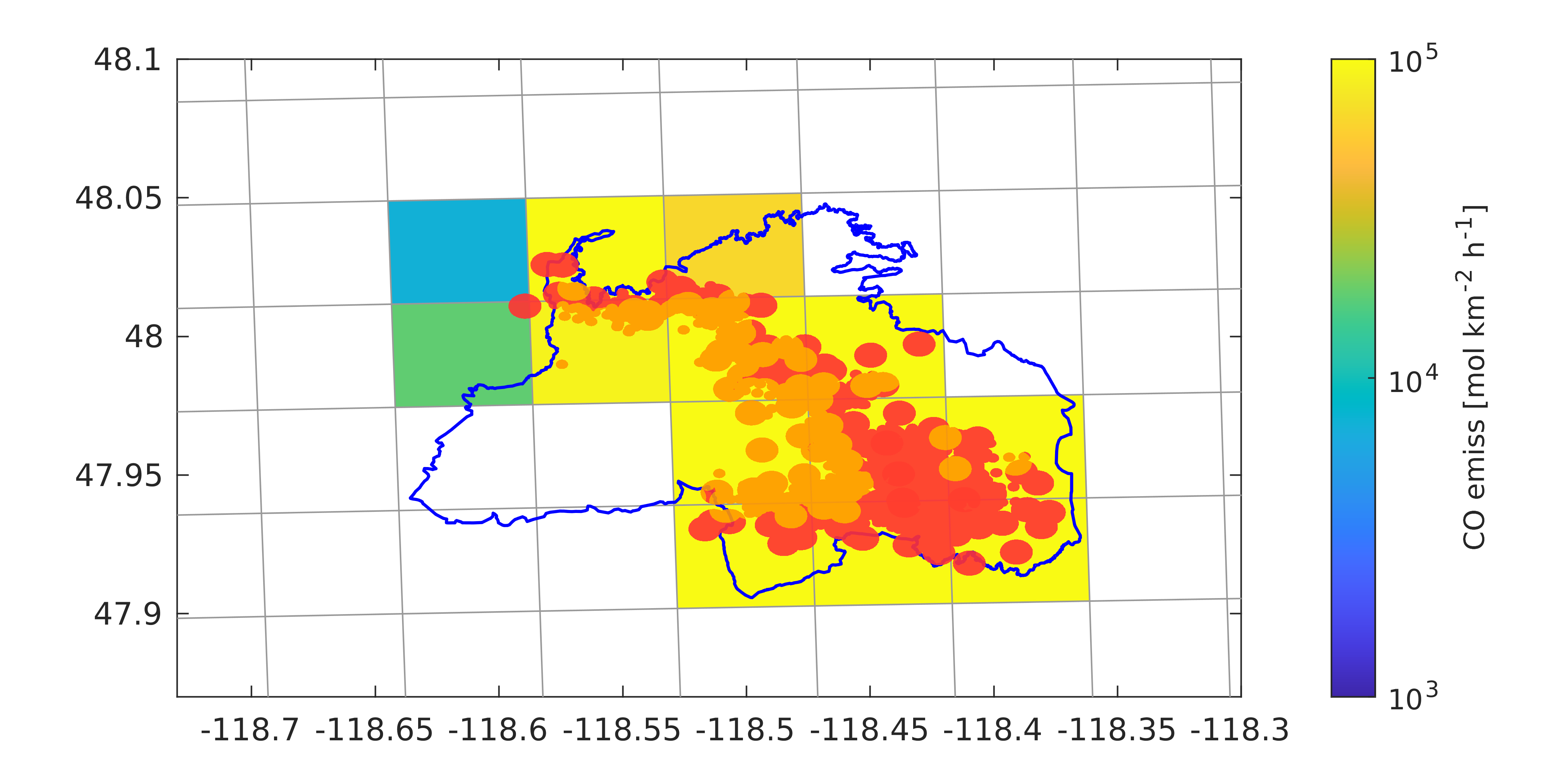


b

a


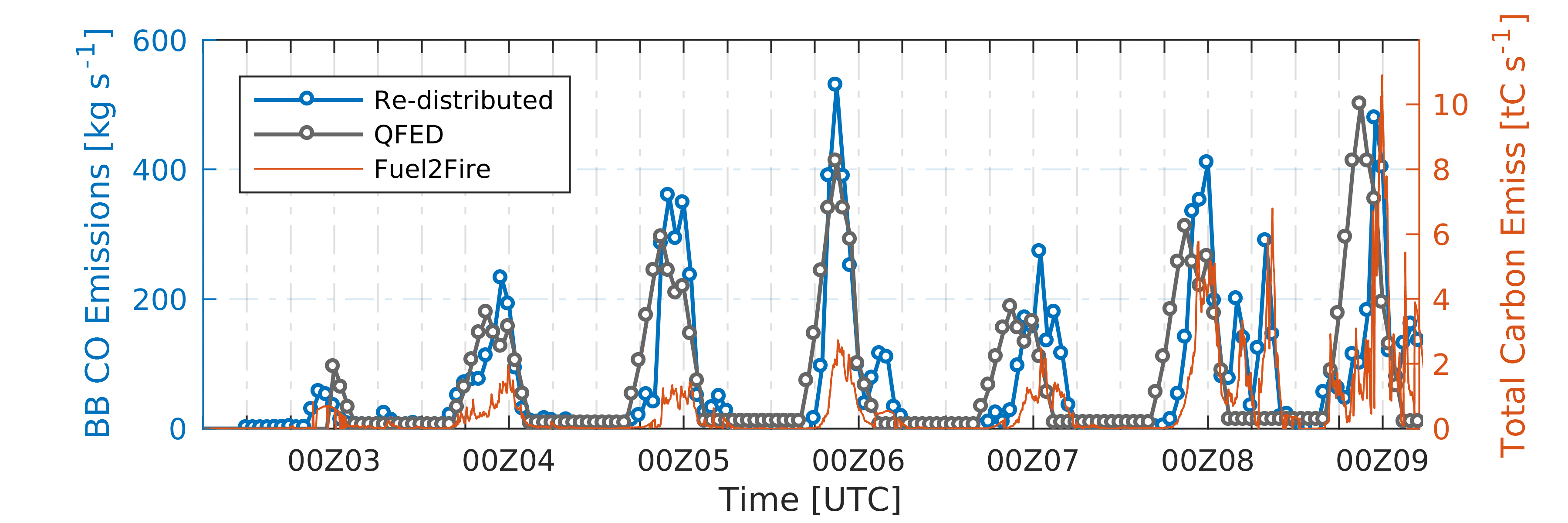

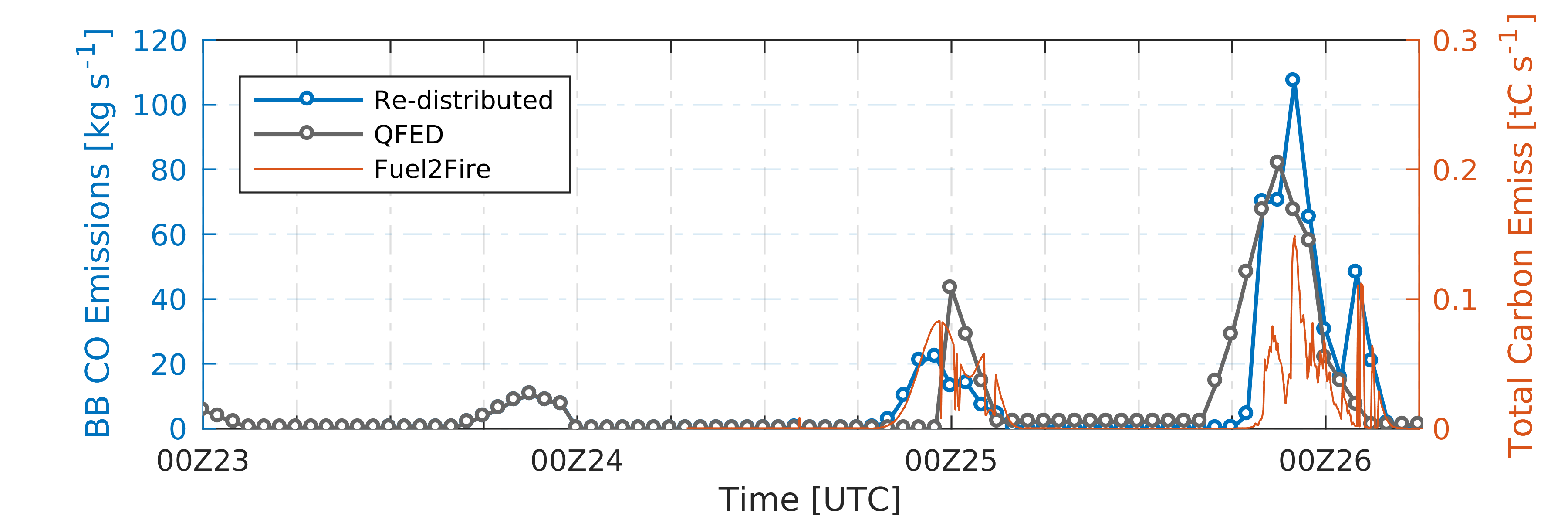

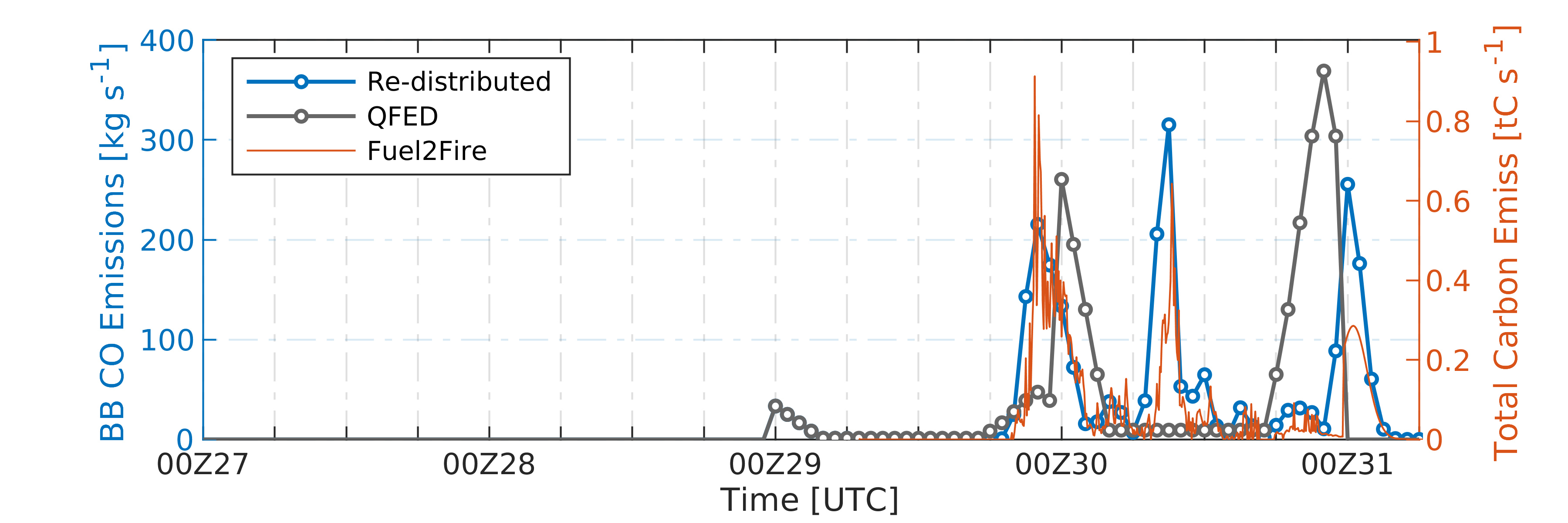


e

d

c

**Figure S1**. Example illustration of the spatial and temporal re-distribution of smoke emissions. (a-c) Williams Flats fire. (a) Gridded burned area based on satellite fire detections on 7 August 2019. (b) Comparison of the observed burned area and remapped emissions at 23:00 UTC 7 August 2019. The red- and orange-colored circles stand for observed new and residual fire detection pixels based on MODIS and VIIRS thermal anomaly data, archived with the Fuel2Fire data. The residual pixels correspond to the ones located within the fire perimeter of the previous day, and the new pixels are those newly occurred on the current day. (c) Time series of bottom-up total carbon emission estimates from the Fuel2Fire data (light red) and hourly smoke CO emissions converted from QFED daily data using the emission processor “fire_emiss” developed by NCAR (grey) and by temporal re-distribution based on the Fuel2Fire inventory (blue). (d-e) Similar as (c), but for Shady and Tucker fire, respectively.


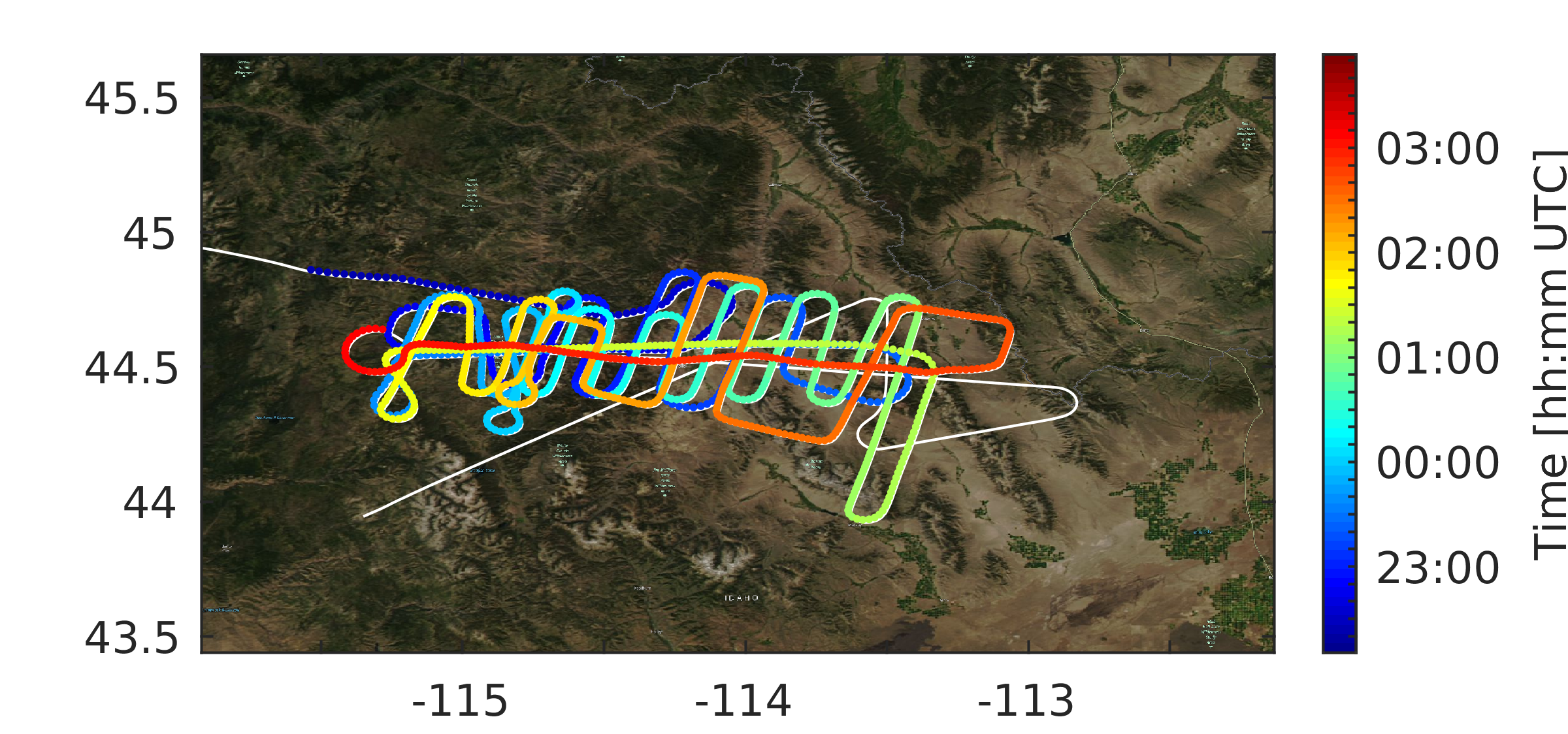

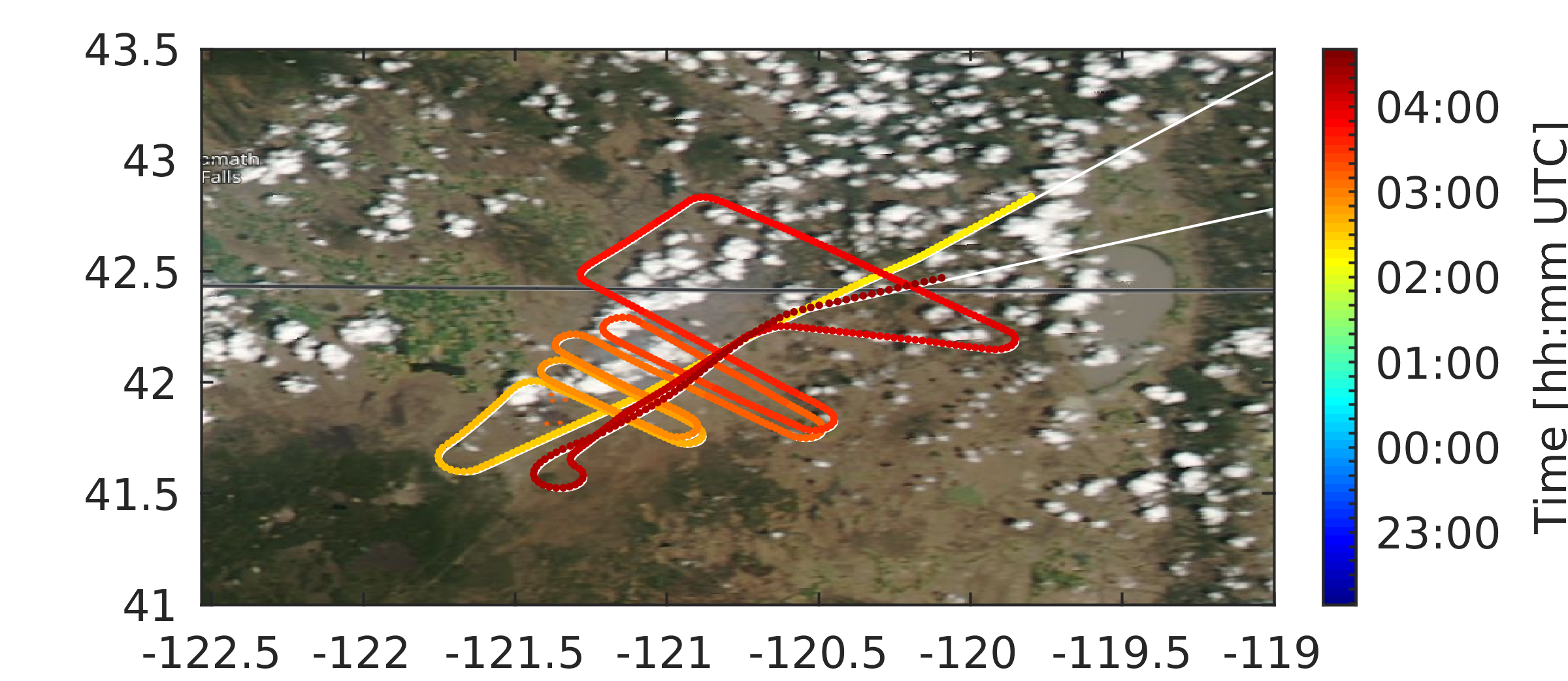

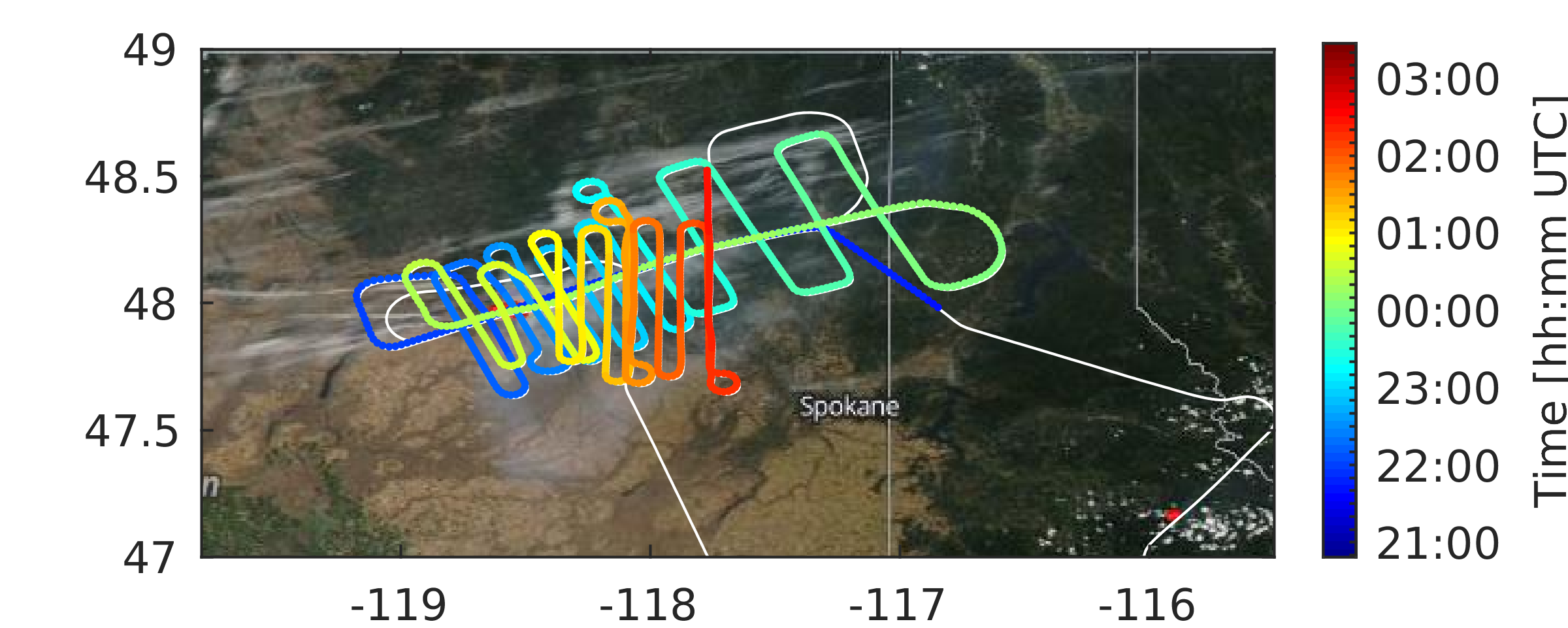

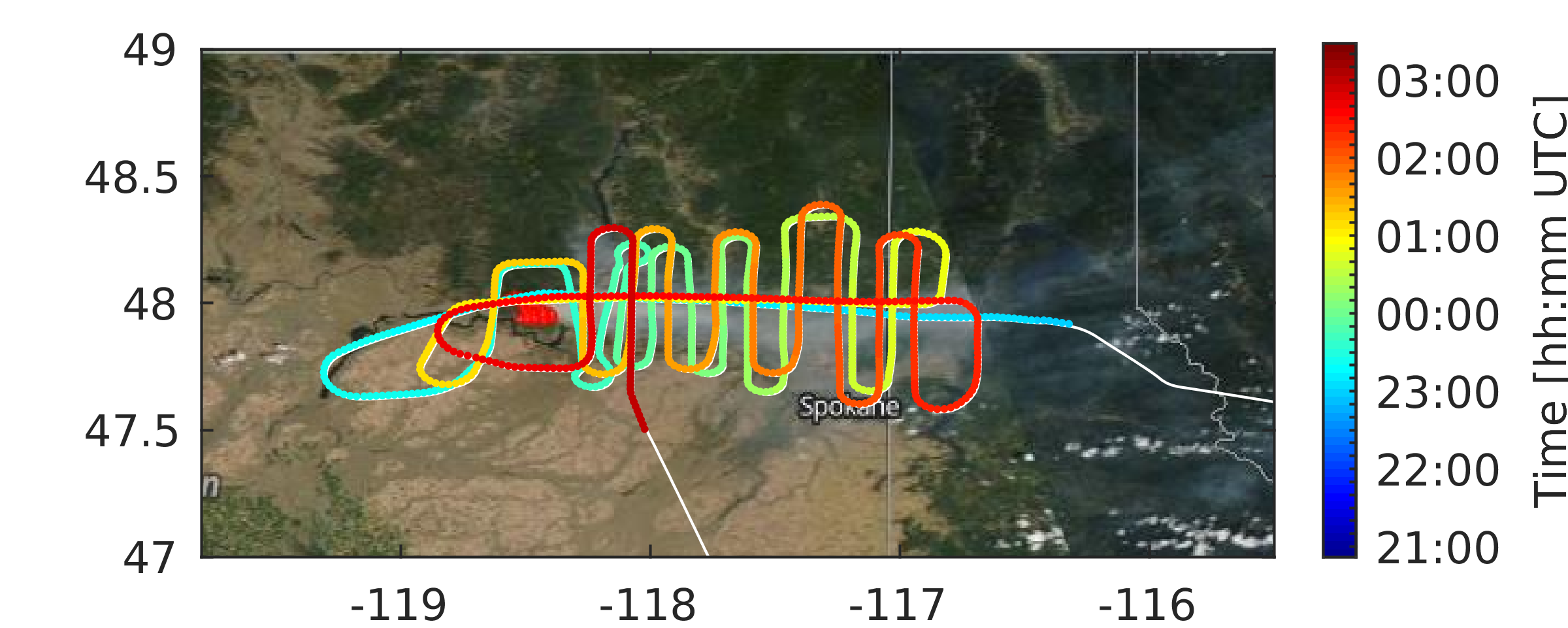


1. (b)

(c) (d)

**Figure S2**. Maps of flight tracks for (a) Shady fire, 25 July 2019, (b) Tucker fire, 29 July 2019, (c) Williams Flats fire, 3 August 2019, and (d) Williams Flats fire, 7 August 2019 (all the dates are in local time). The selected flight transects are highlighted in color by sampling time (units: hh:mm UTC), overlayed on the Aqua MODIS visible images on the same dates acquired from NASA Worldview (<https://worldview.earthdata.nasa.gov/>). The x- and y-axis indicate longitude and latitude respectively.


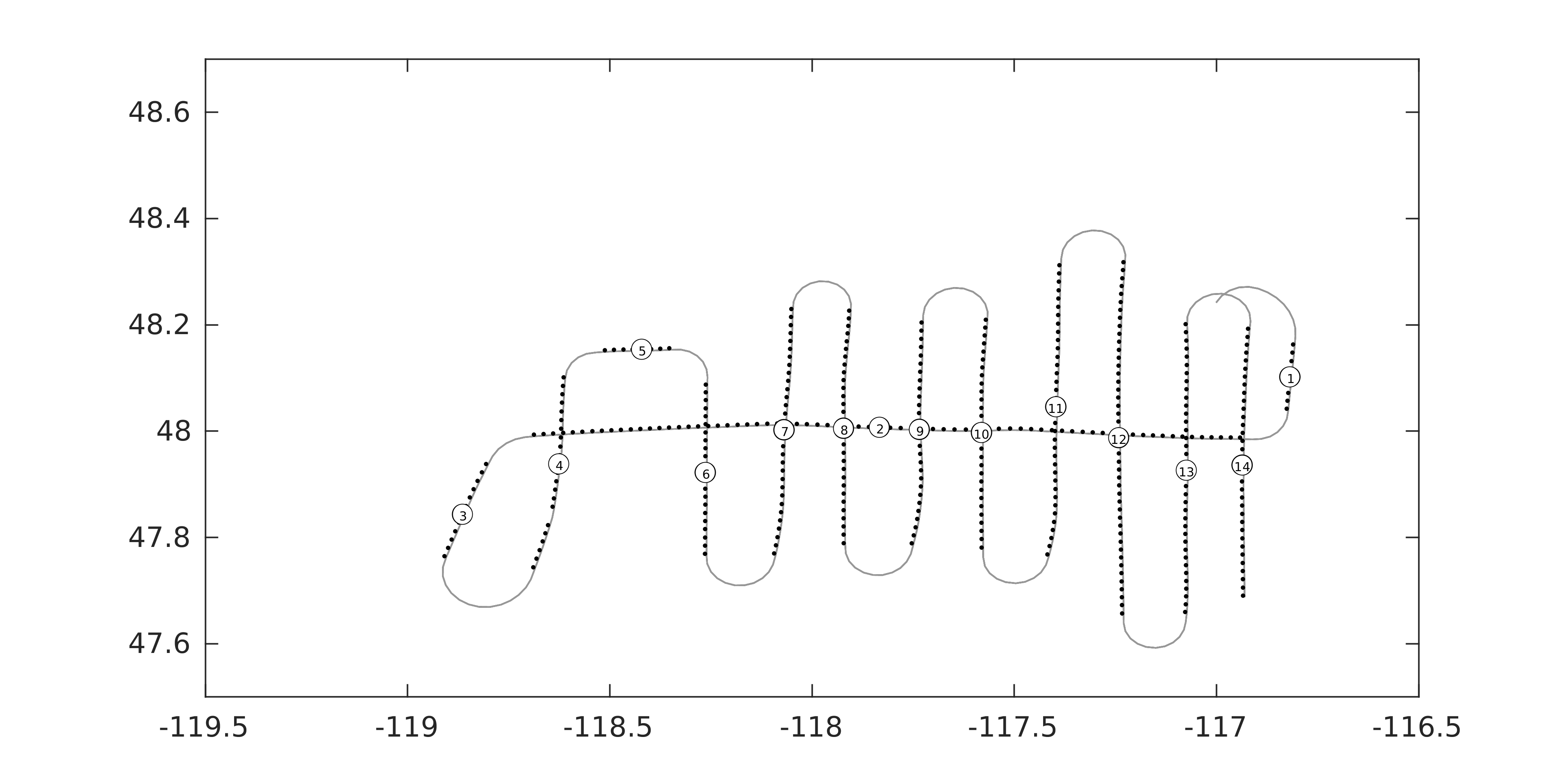


**Figure S3**. Flight track sampling the Williams Flats fire during 00:47 – 02:20 UTC, 7 August 2019. See Fig. S2d for the whole flight track on that day. The black dots represent the location of 10-s resolution lidar profiles with valid data at any vertical level. Data are missing during flight turning due to aircraft interference. Circles with numbers show flight segments between the flight turns. The x- and y-axis indicate longitude and latitude respectively.


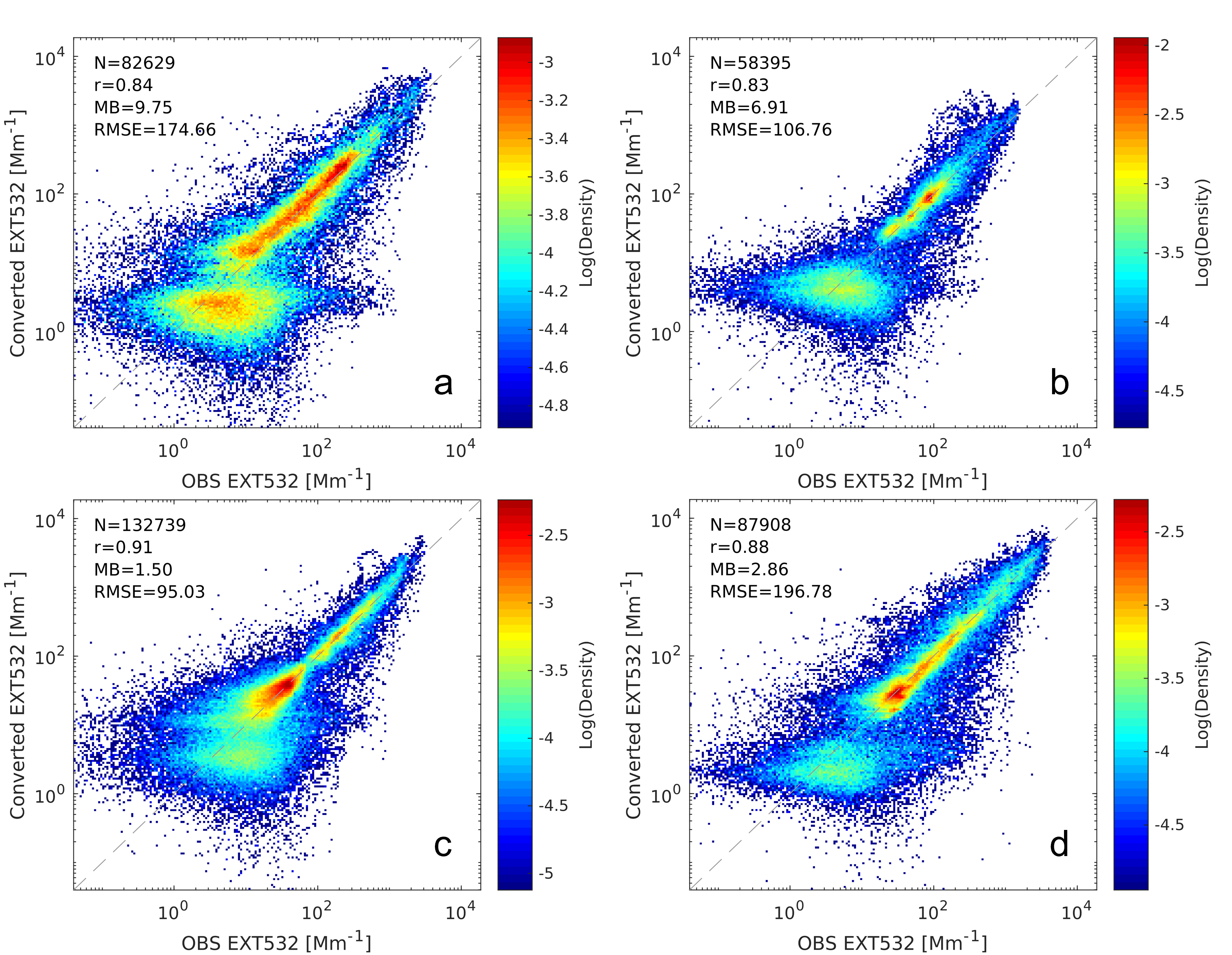


**Figure S4**. Density plots of the number fraction of lidar-observed and converted aerosol extinction at 532 nm (EXT532) in each bin. (a) Shady fire, 25 July. (b) Tucker fire, 29 July. (c) Williams Flats fire, 3 August. (d) Williams Flats fire, 7 August 2019. The data and fraction density are shown in log scale. Total number of points (N), correlation coefficient (r), mean bias (MB), and root-mean-square error (RMSE) are labeled. The grey dashed line represents 1:1.


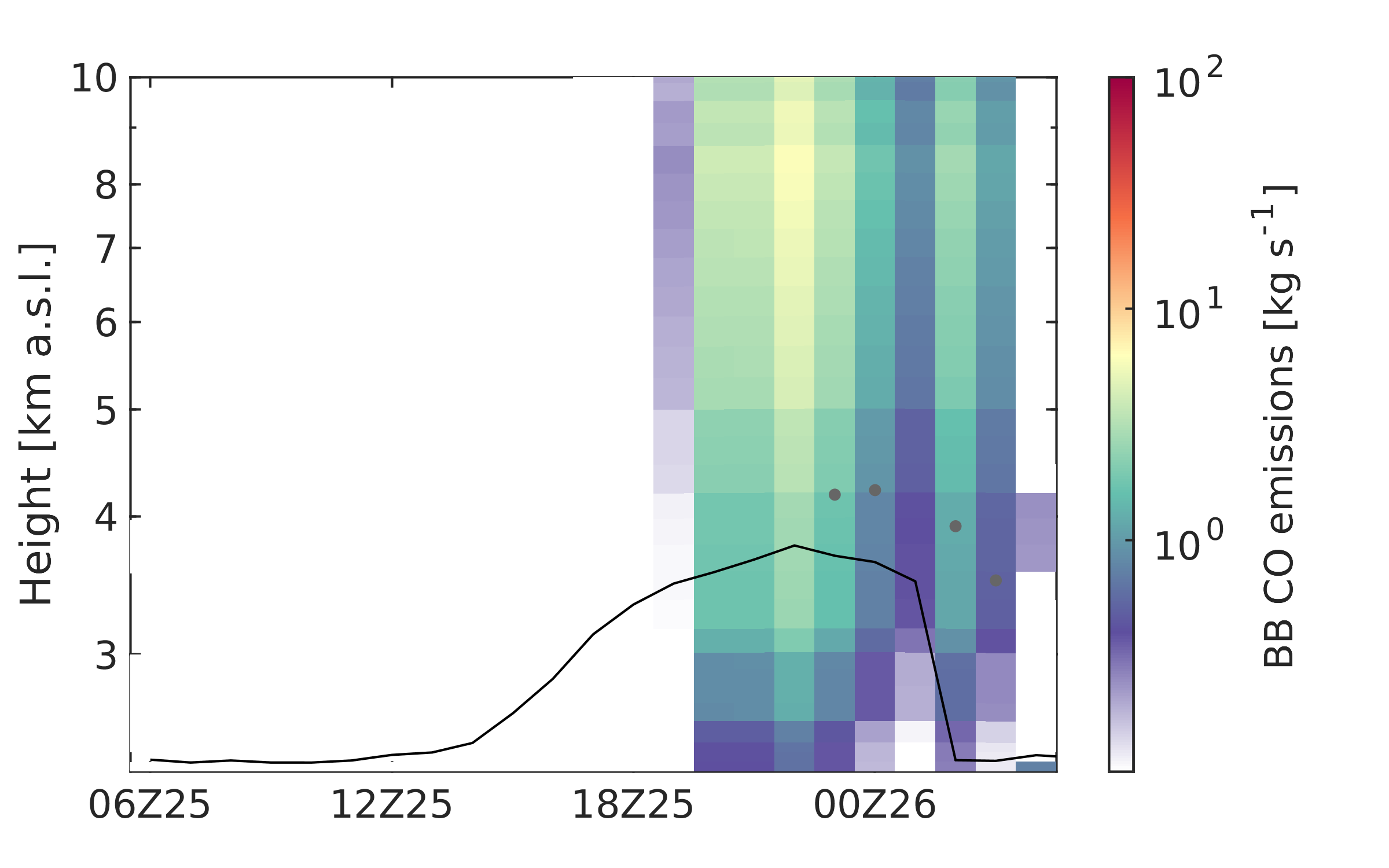

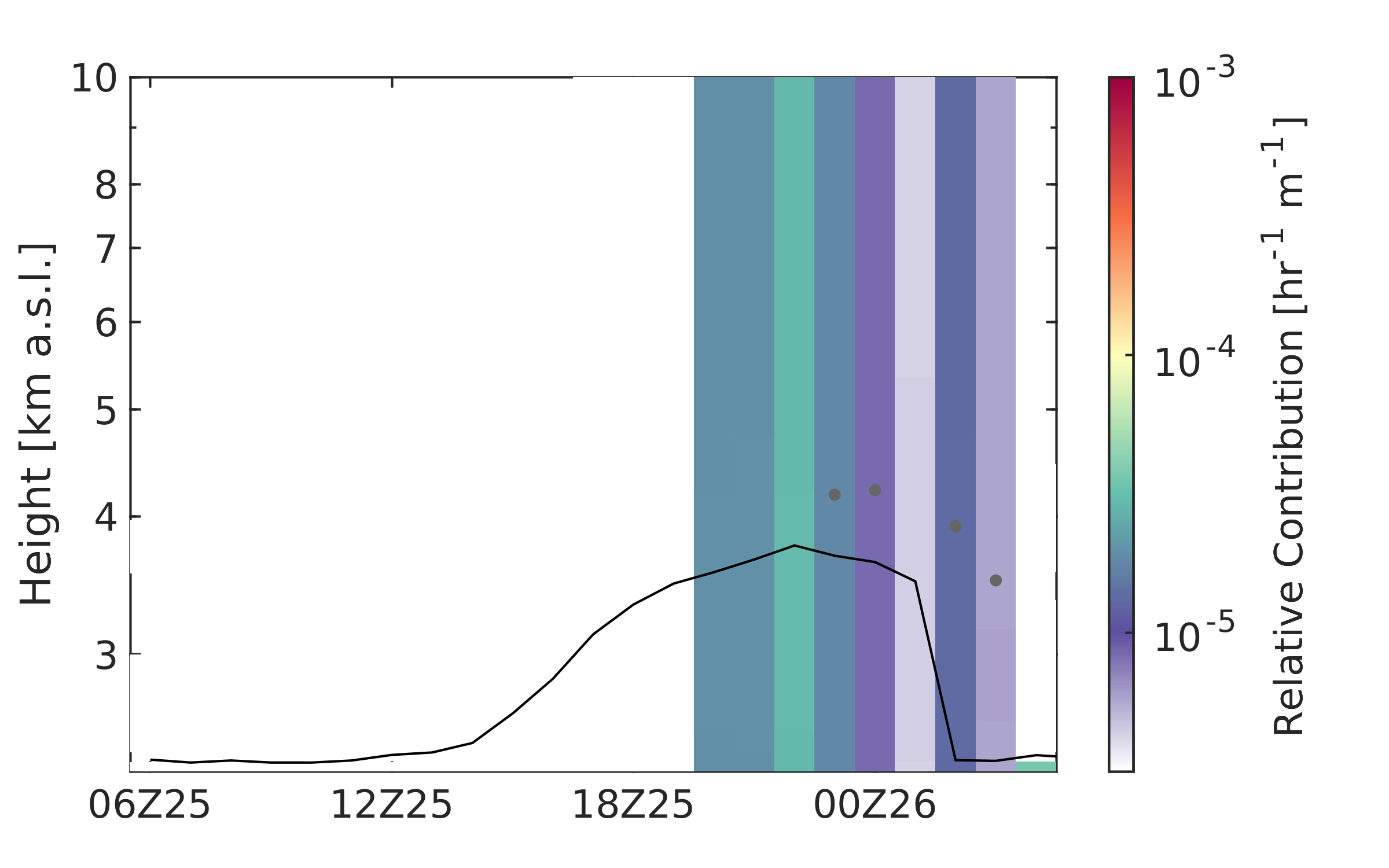


(a) (b)

(c) (d)

(e) (f)

(g) (h)


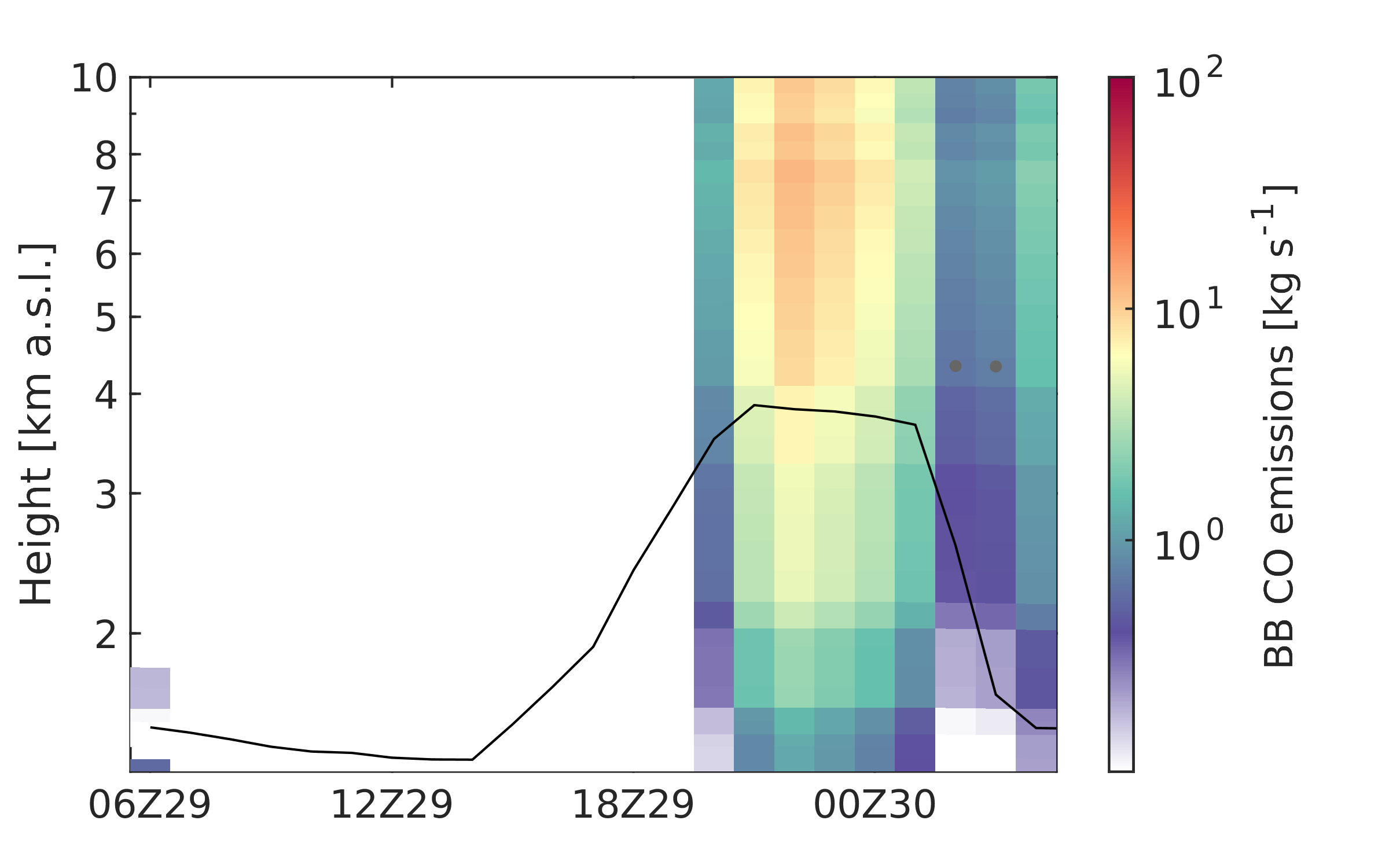

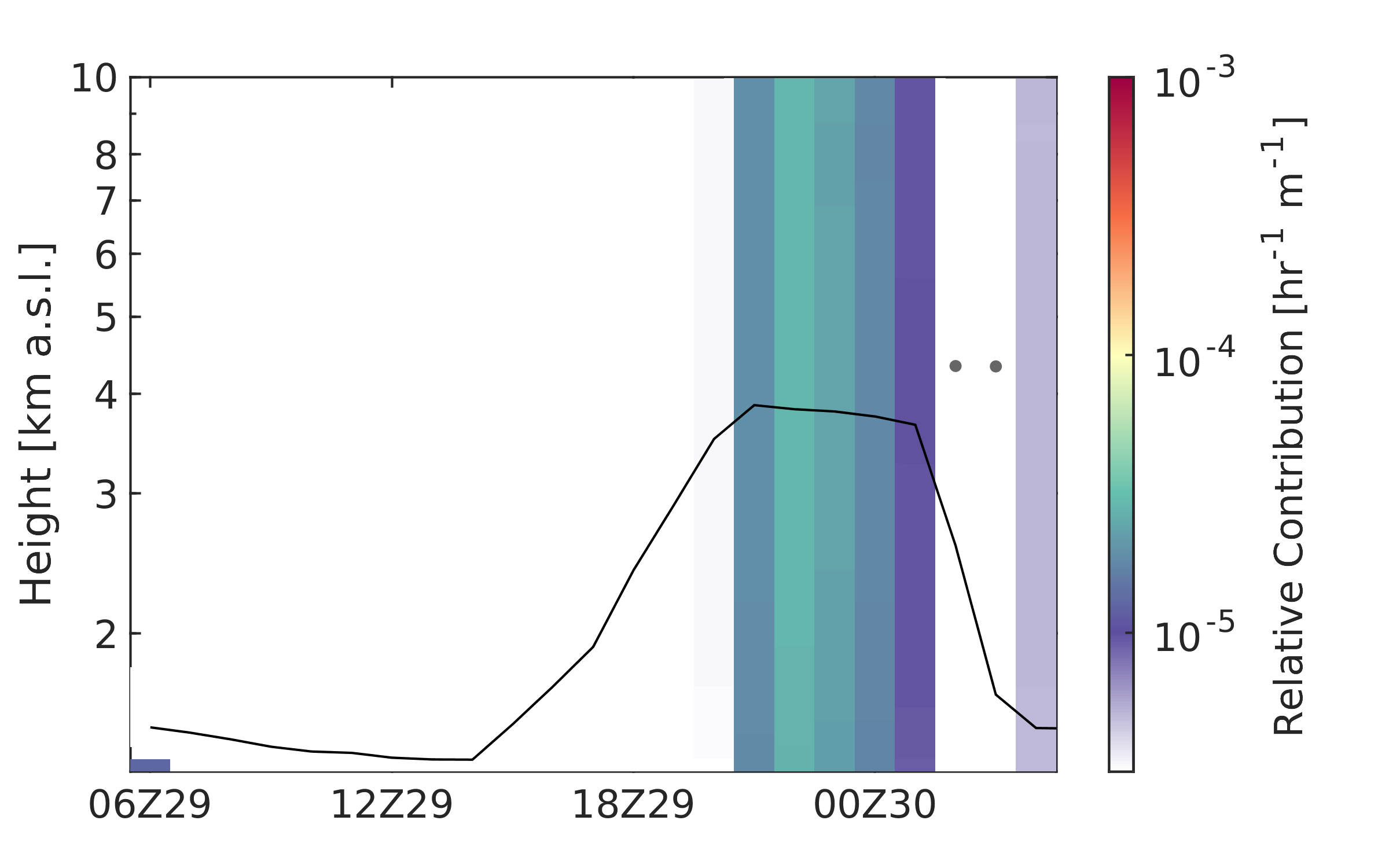


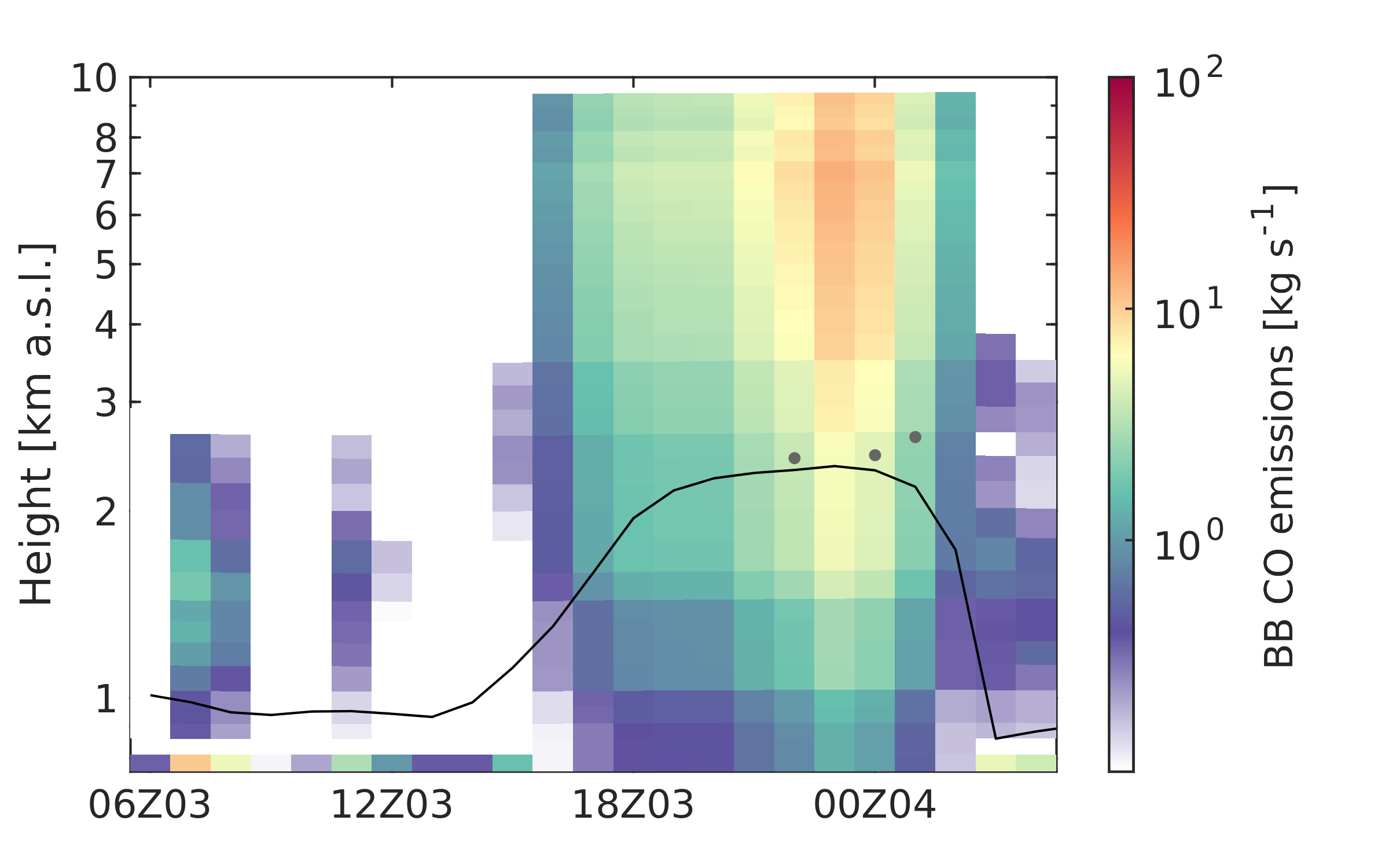

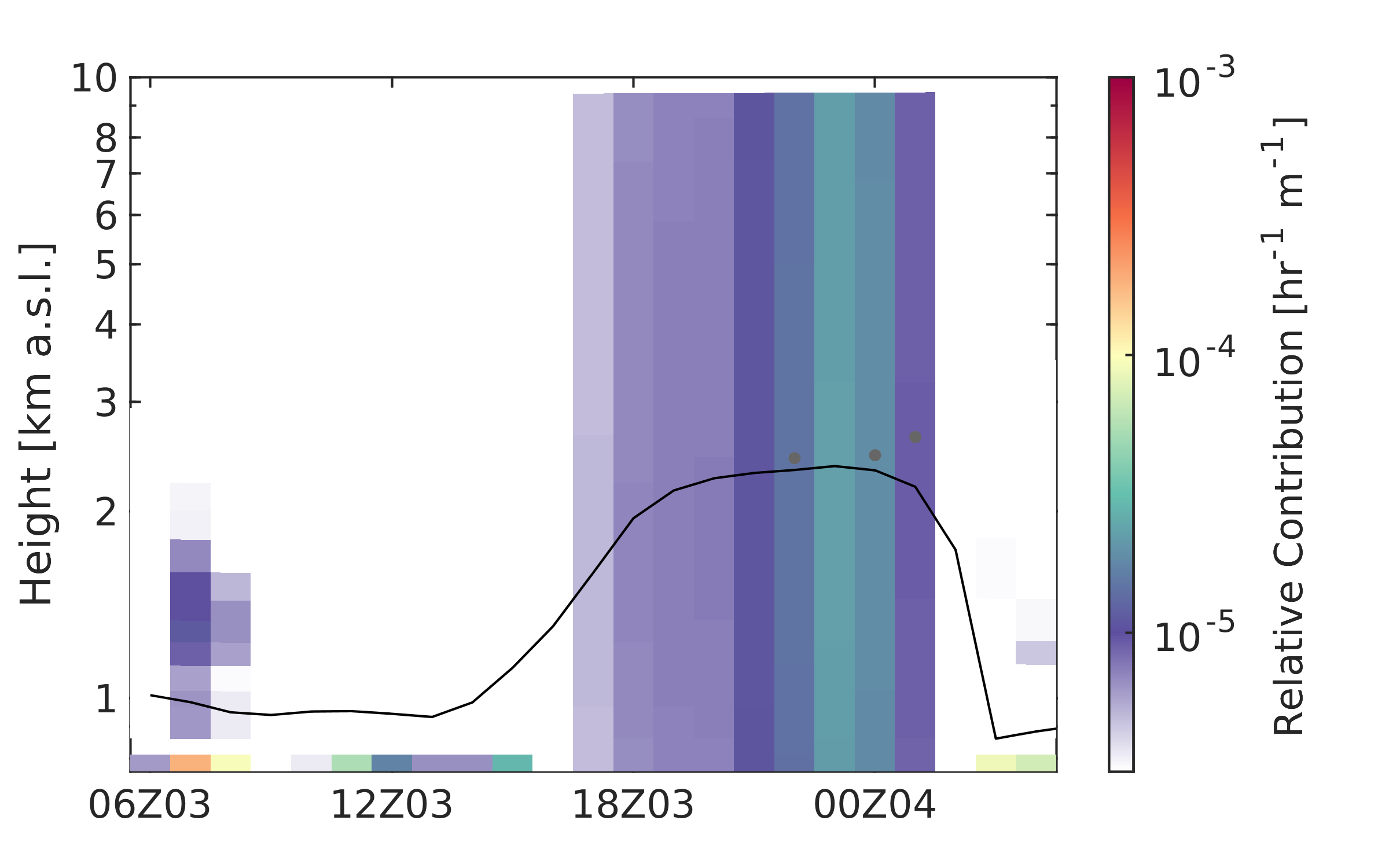


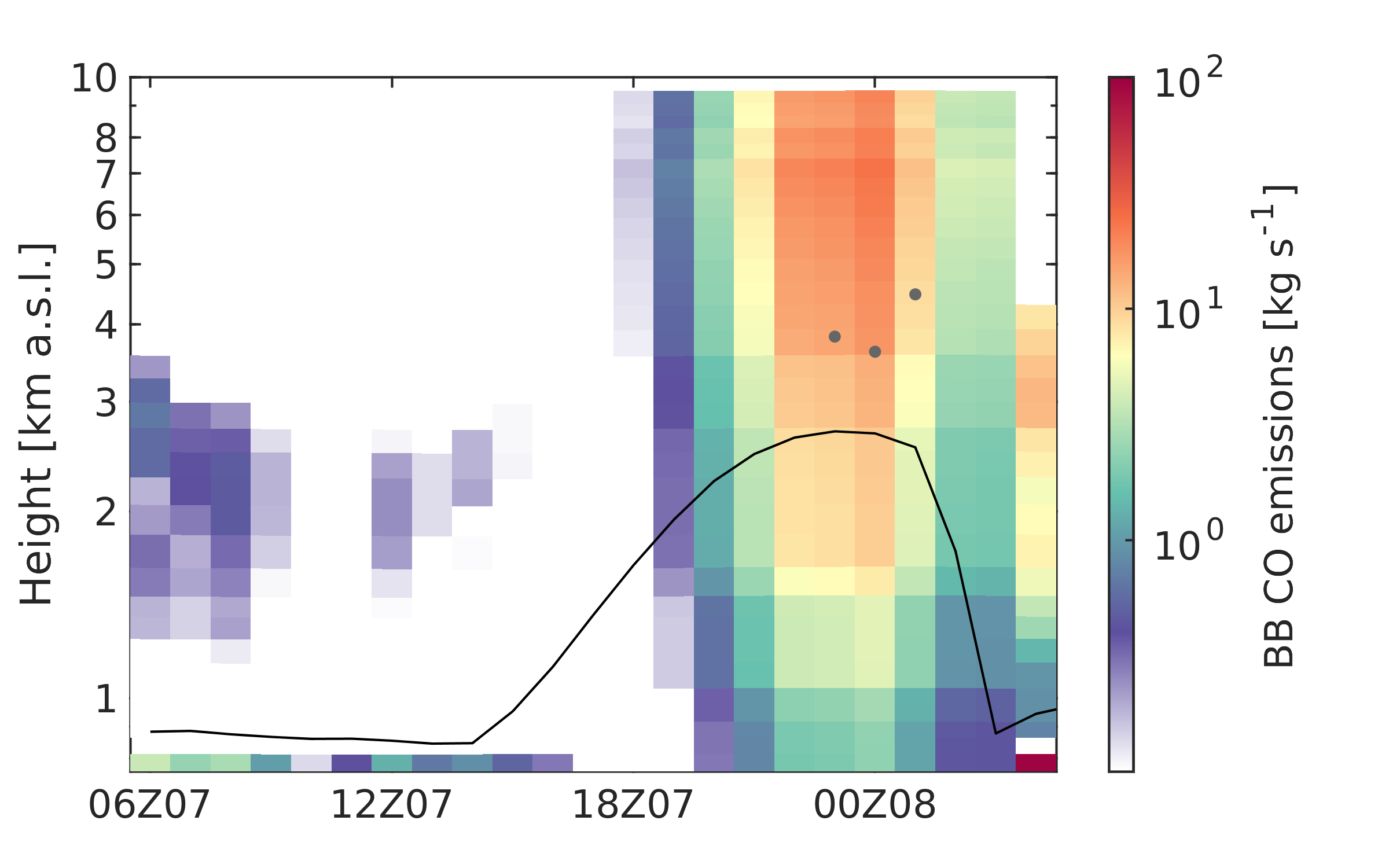

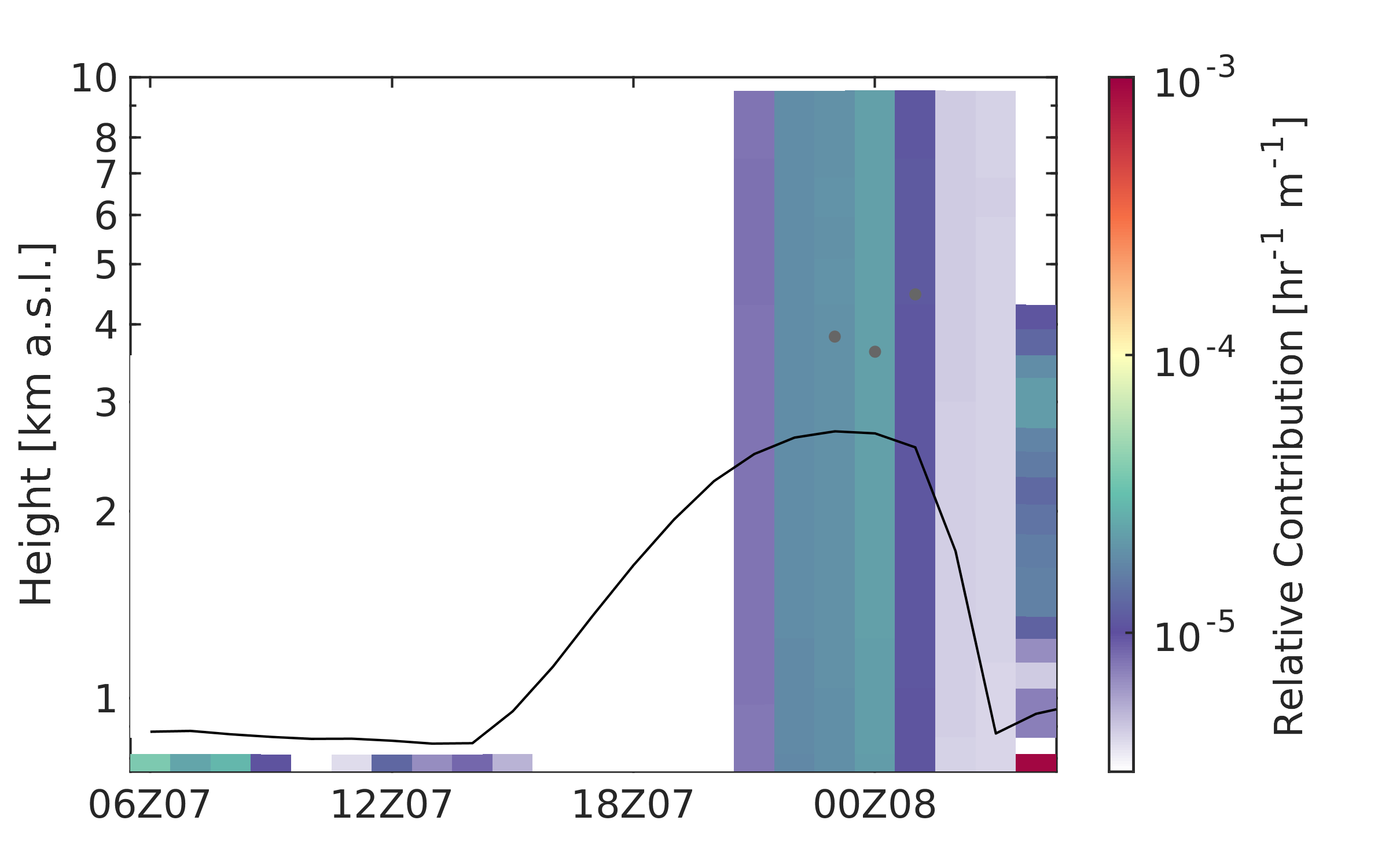


**Figure S5**. Fire CO emissions for the Shady (25 July, a-b), Tucker (29 July, c-d), and Williams Flats fire (3 and 7 August, e-h) in 2019. The left column shows the emissions by hour and model layer. The right column presents relative emission density per hour and per kilometer. The emissions are evenly distributed in vertical direction during the periods of interest (17:00 UTC Jul 25 to 03:00 UTC Jul 26, 18:00 UTC Jul 29 to 04:00 UTC Jul 30, 16:00 UTC Aug 3 to 02:00 UTC Aug 4, and 17:00 UTC Aug 7 to 03:00 UTC Aug 8 for the four sampling dates analyzed here, respectively). Note that the heights are shown in log scale and time in UTC (hhZdd).


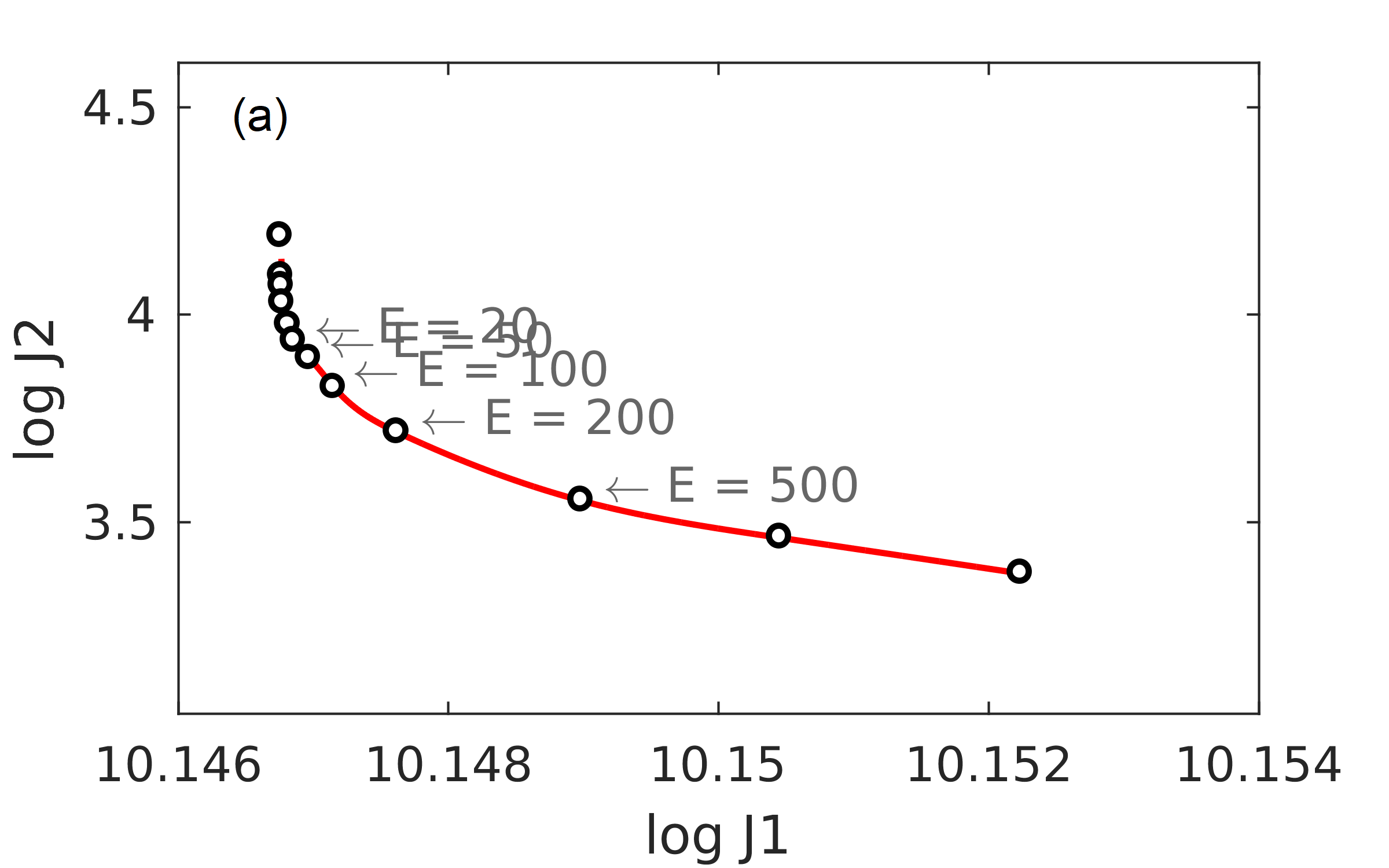

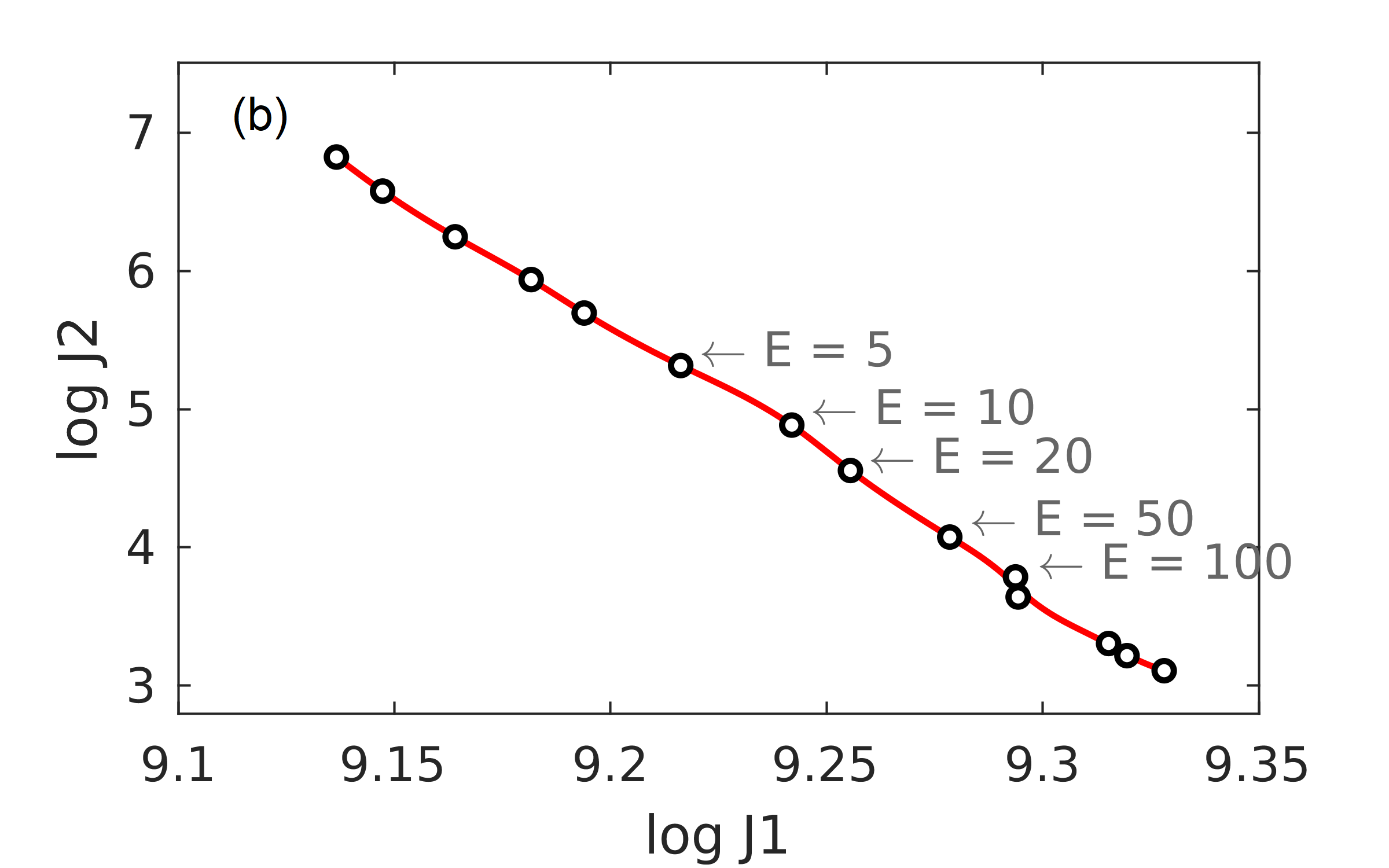


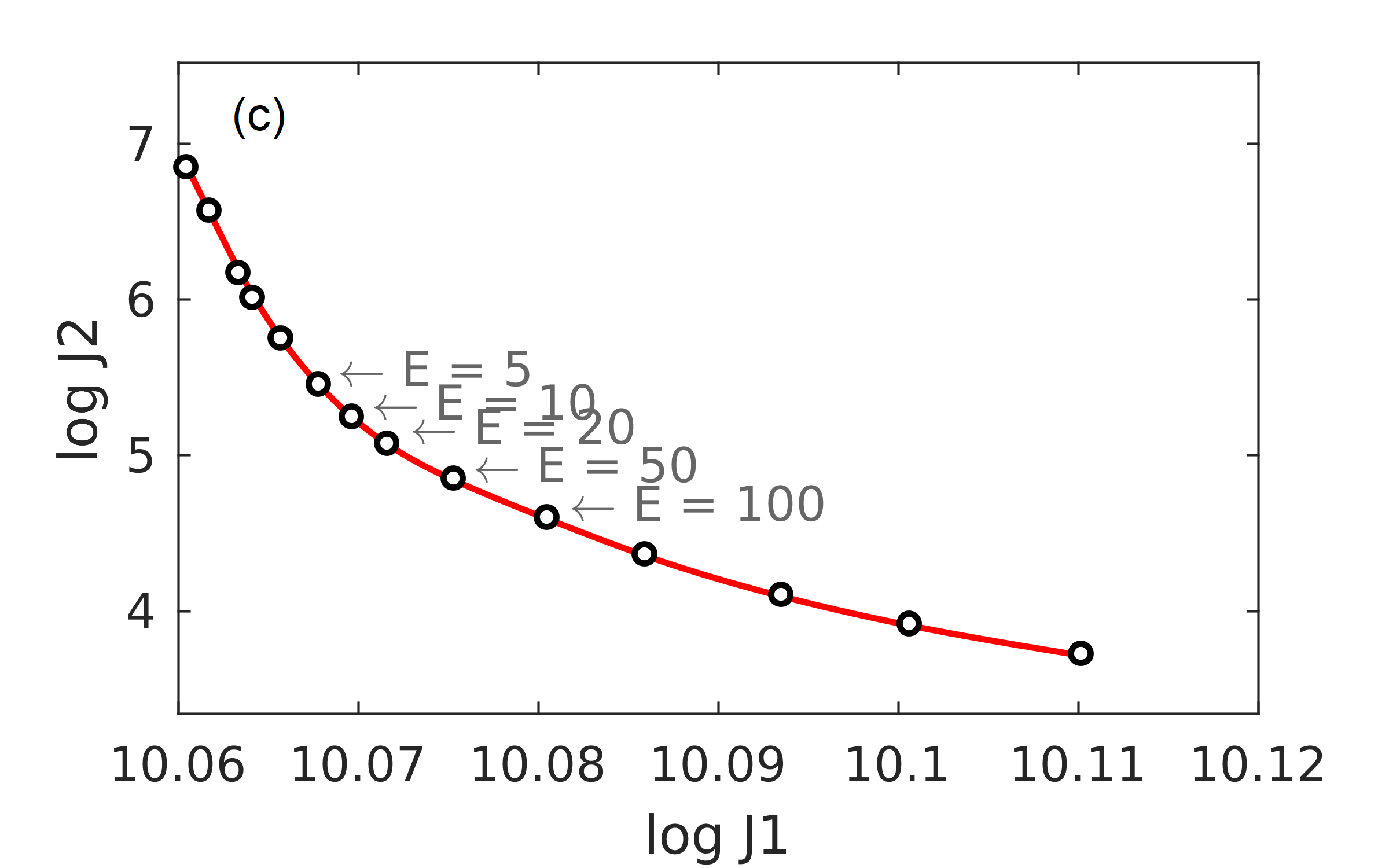

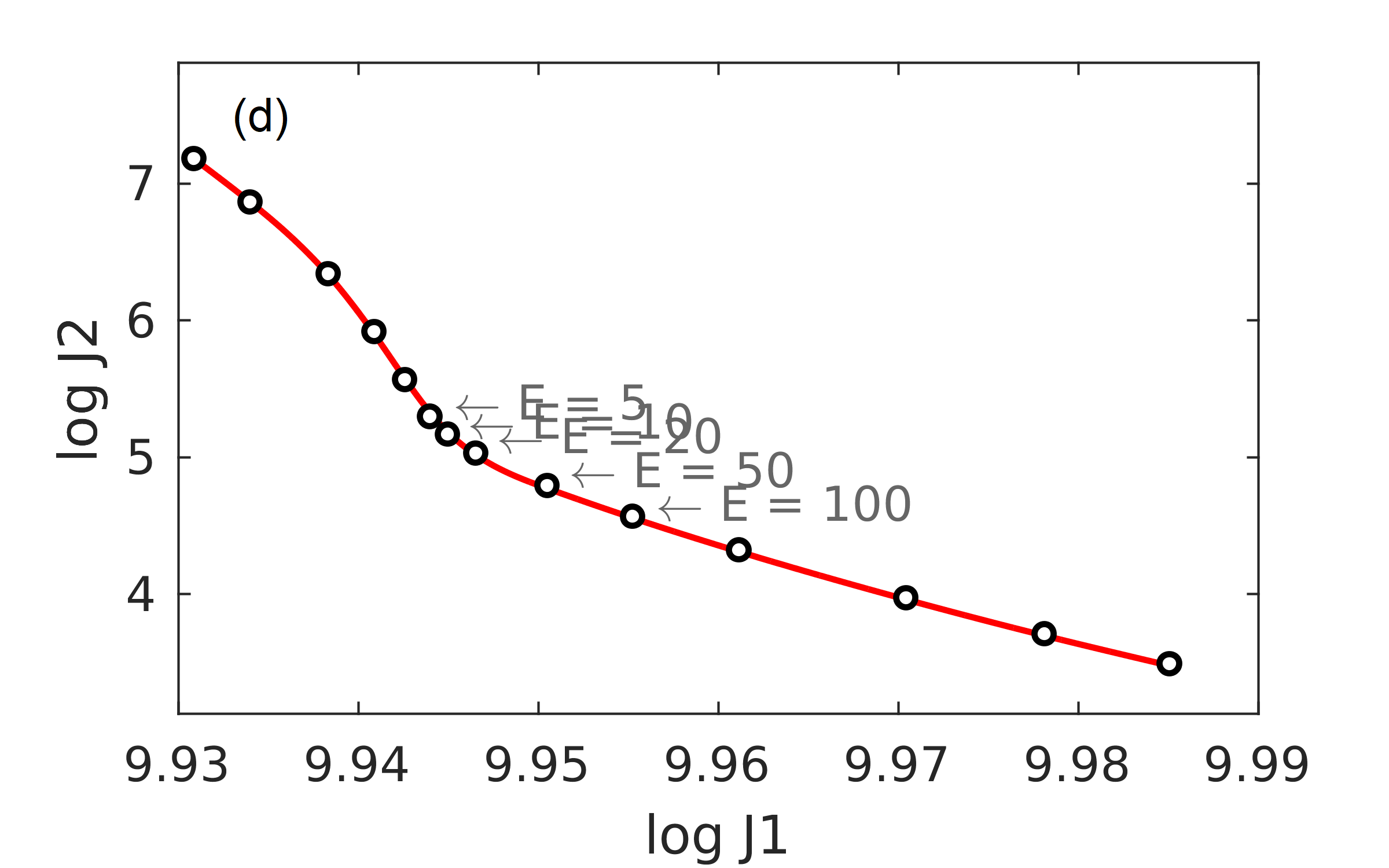


**Figure S6**. J1 vs J2 as a function of E (L-curve plot) for inversions performed for (a) Shady fire on July 25, (b) Tucker fire on July 29, and Williams Flats fire on (c) August 3 and (d) August 7, 2019.


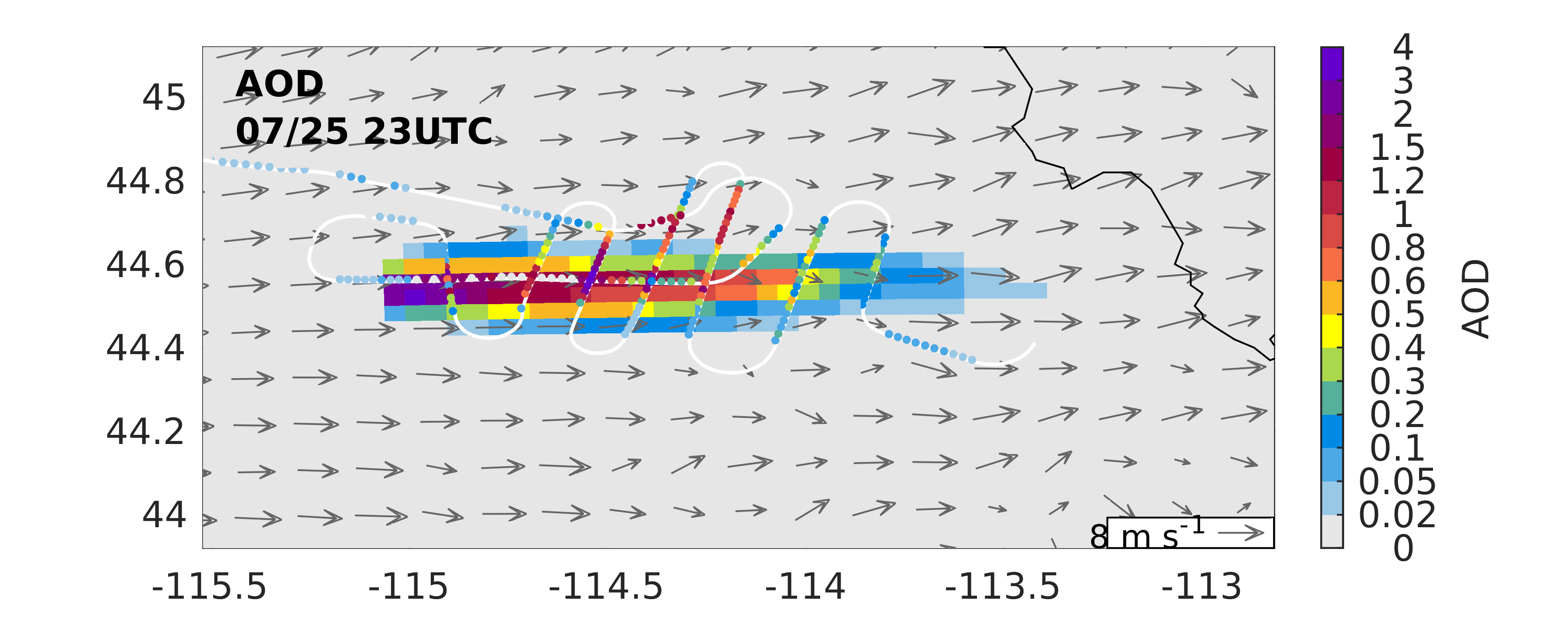

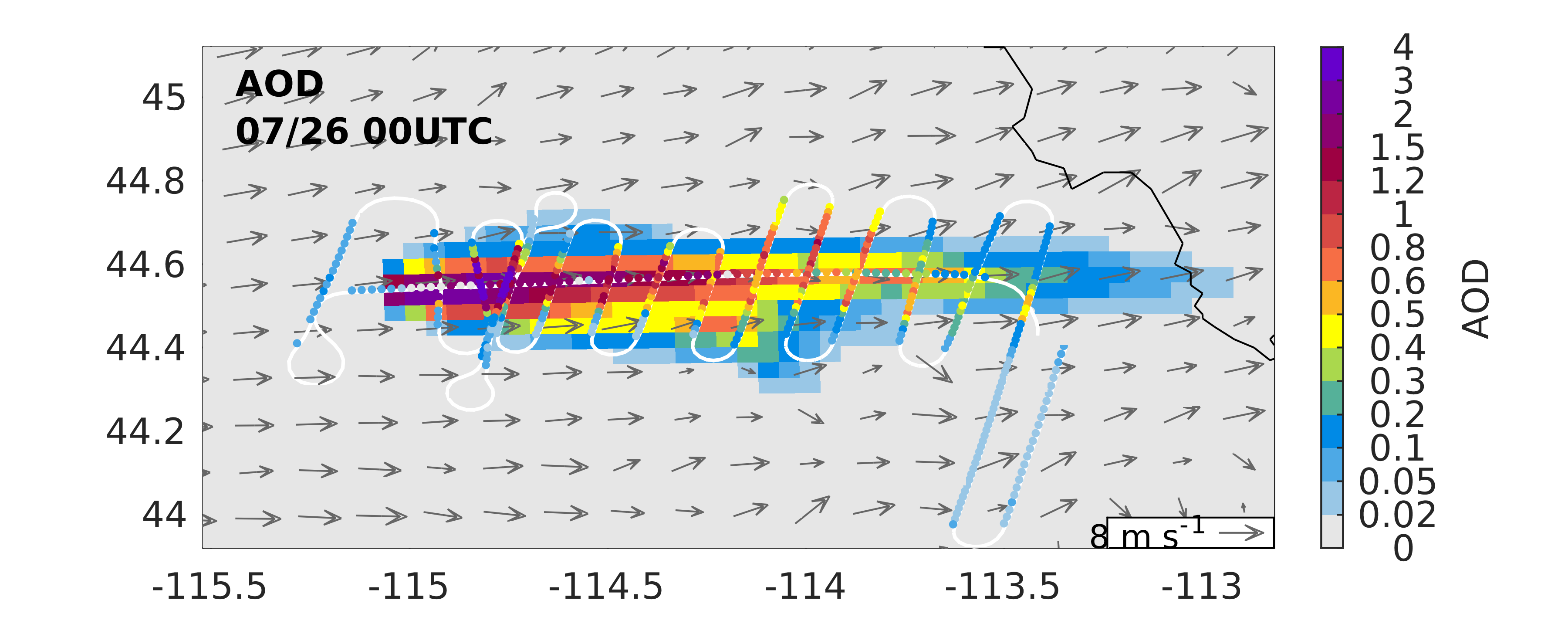


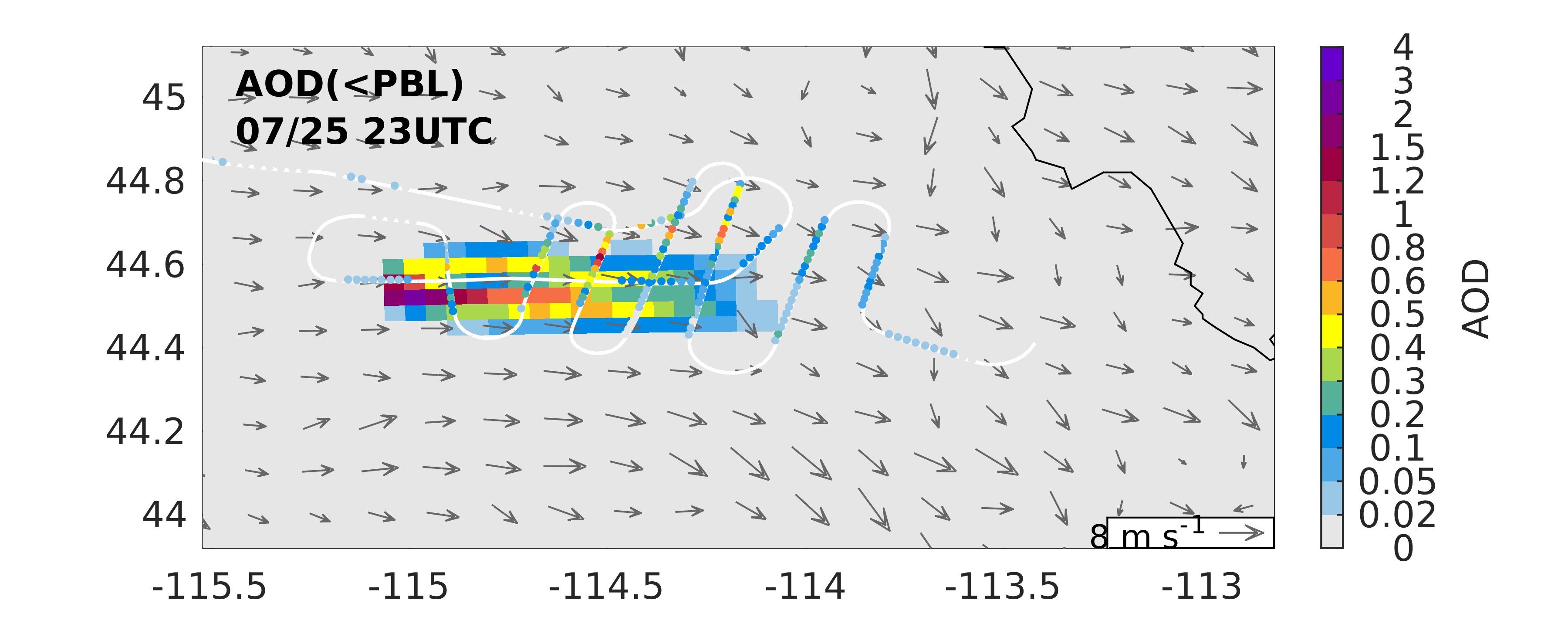

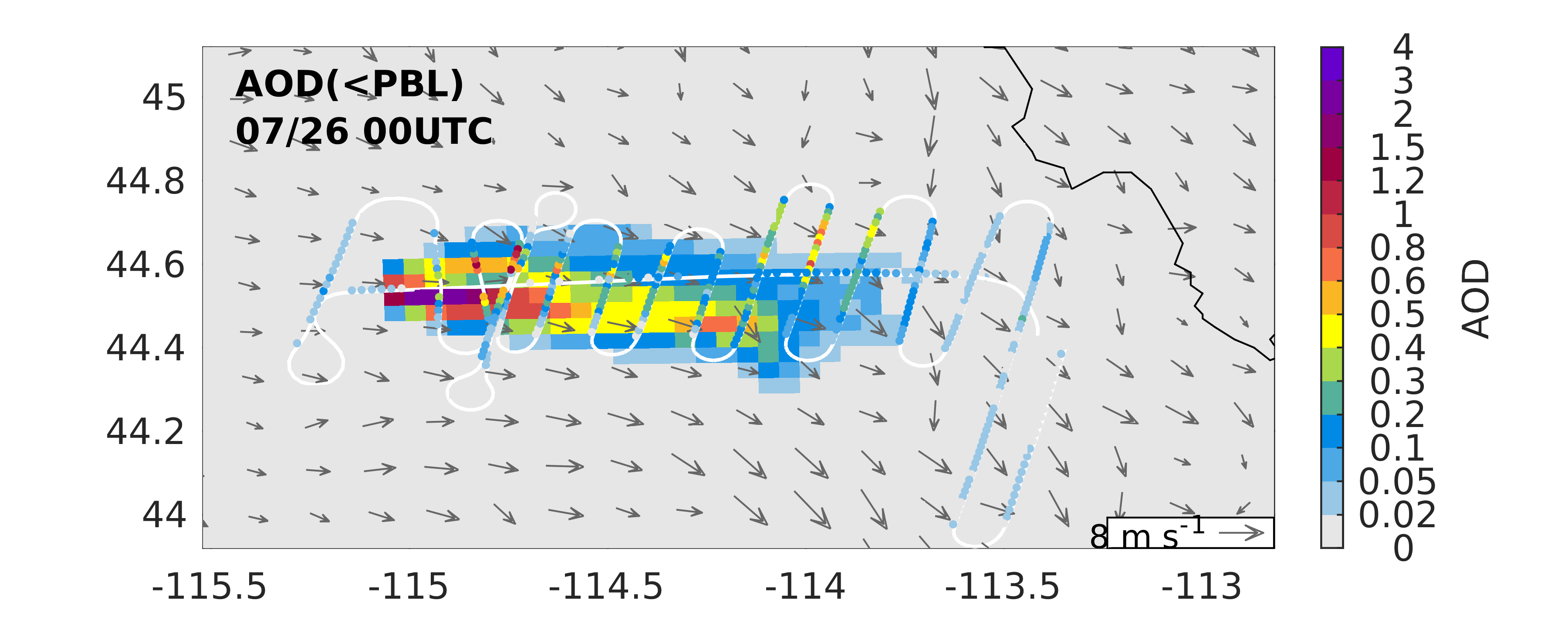


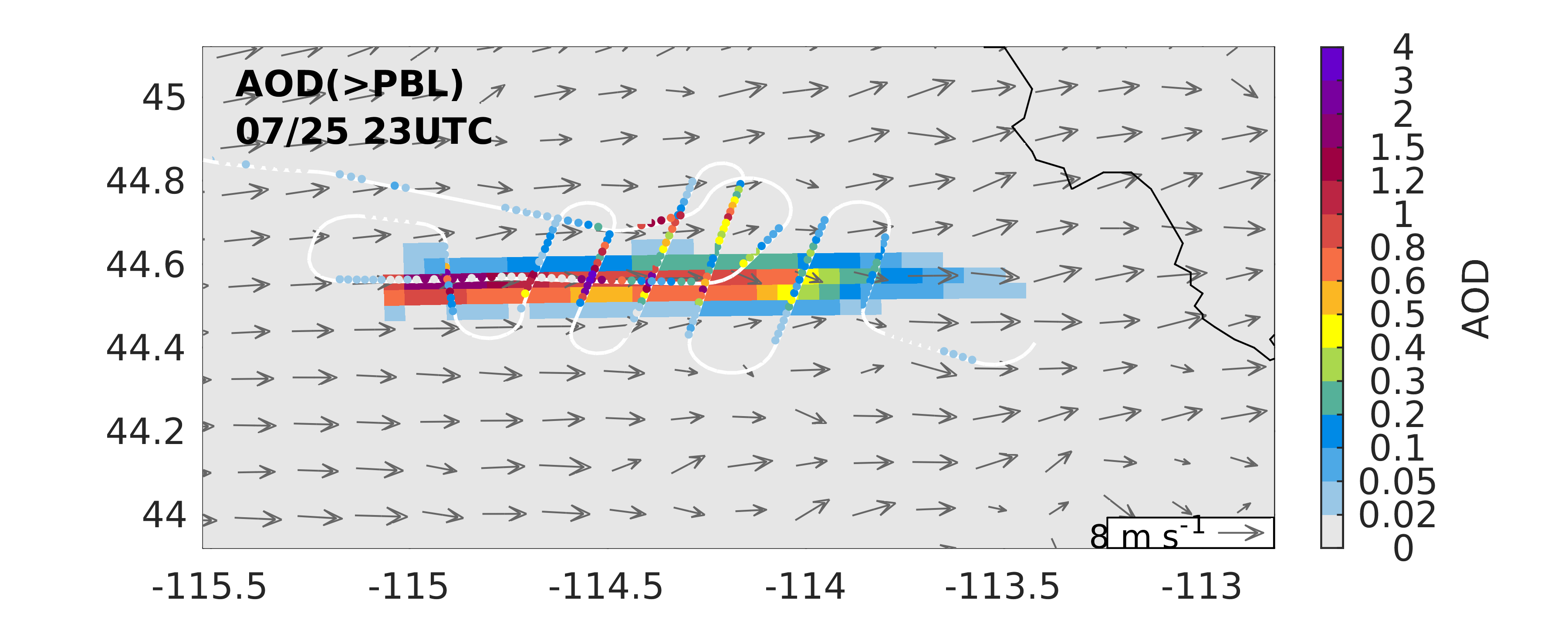

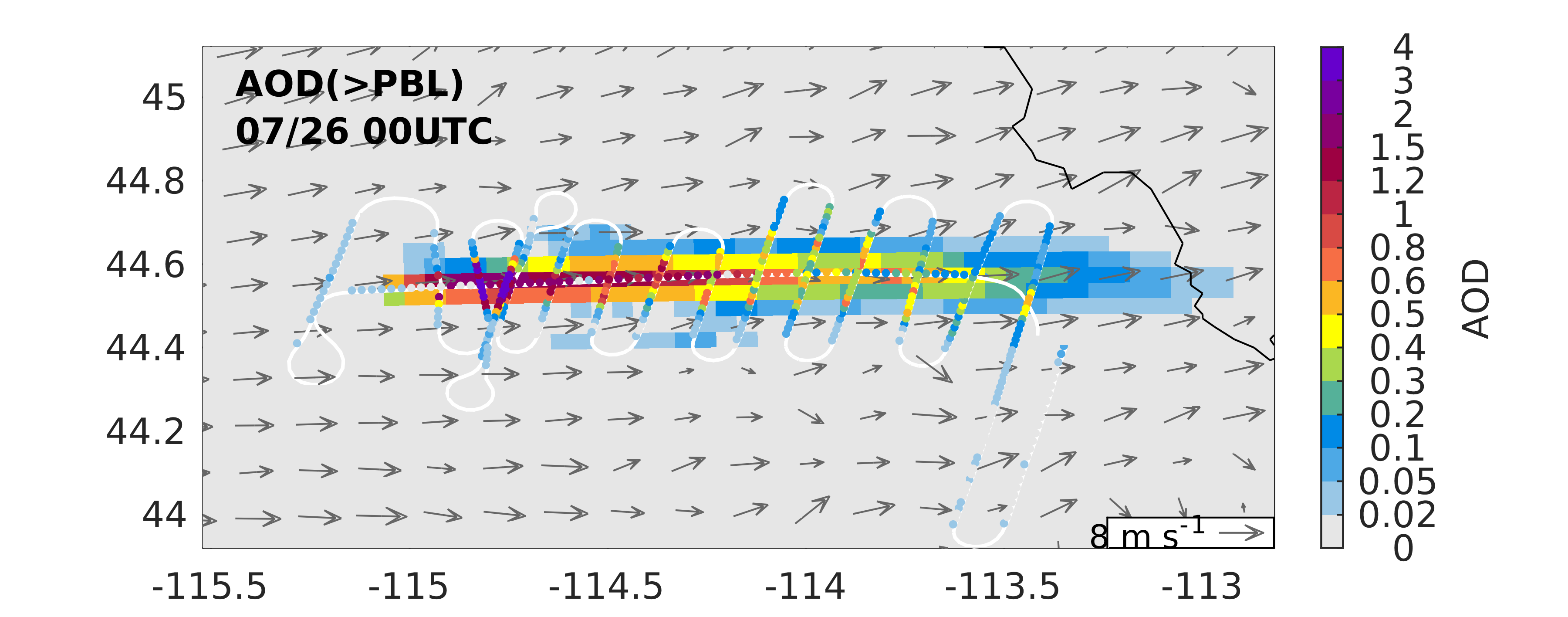


**Figure S7**. Comparison of simulated smoke AOD enhancements and observations from DIAL-HSRL at 23:00 UTC 25 July and 00:00 UTC 26 July 2019. Results are shown by integrated smoke AOD for total column atmosphere (top row) with wind vectors at 2 km above ground level (a. g. l.), below the PBL height (middle row) with wind at 0.6 km a. g. l., and above the PBL (bottom row) with wind at 2 km a. g. l., respectively. The PBL height is derived from model diagnosis.


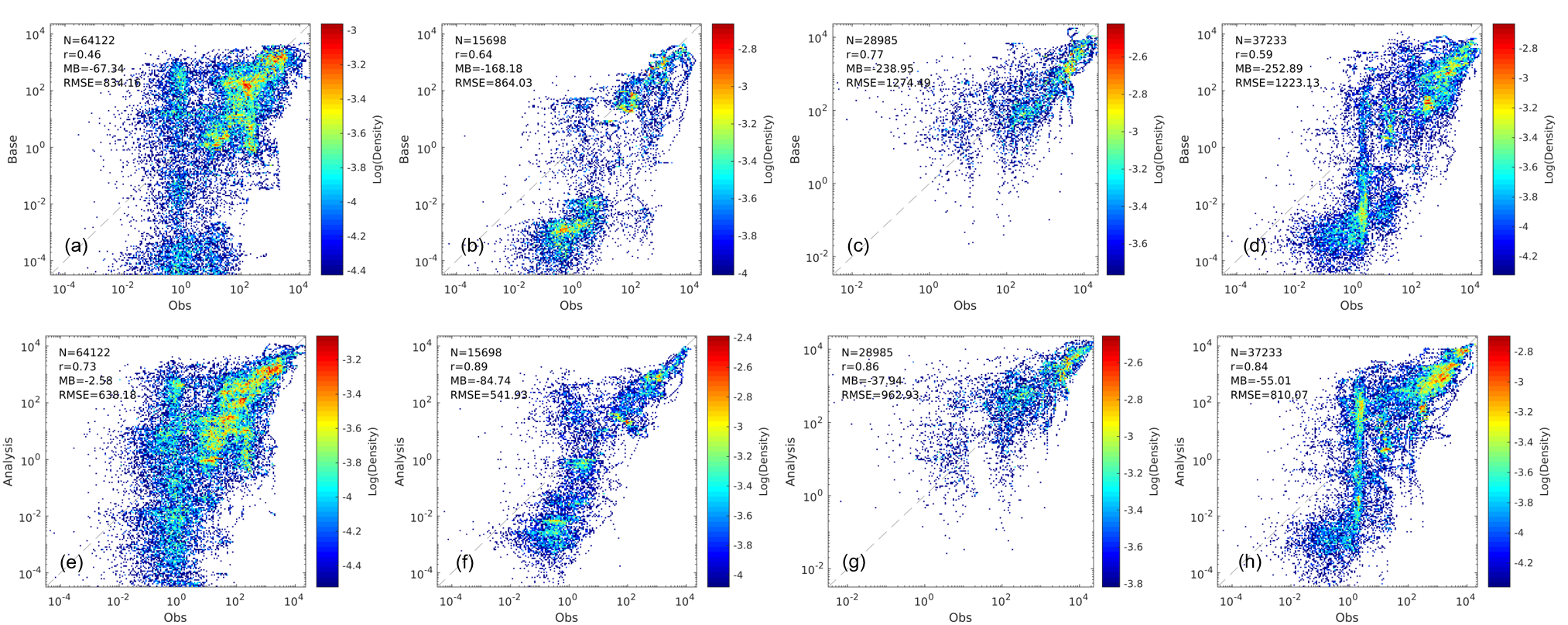


**Figure S8**. Density plots of the fraction of number model-observation pairs of the assimilated observables in each bin. The columns correspond to sampling dates of 25 July (a, e), 29 July (b, f), 3 August (c, g), and 7 August (d, h) 2019, respectively. Results are shown for the Base run (upper row) and the Analysis run (with constrained emissions, lower row). The observations, simulations, and density of fraction are shown in log scale. Total number of points (N), correlation coefficient (r), mean bias (MB), and root-mean-square error (RMSE) are labeled on each panel. The grey dashed line represents 1:1.


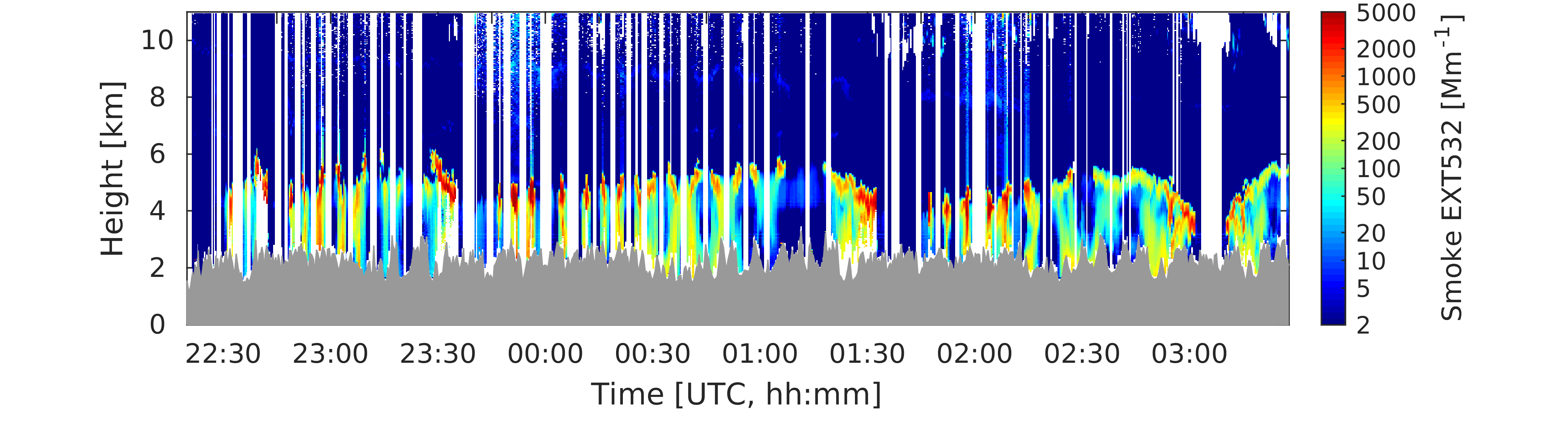

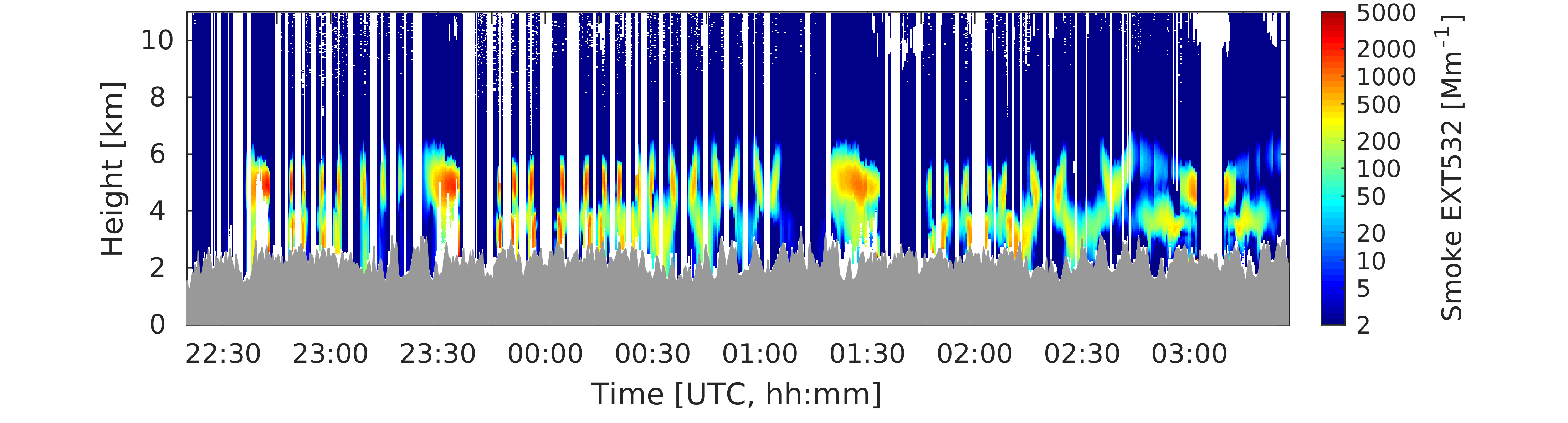

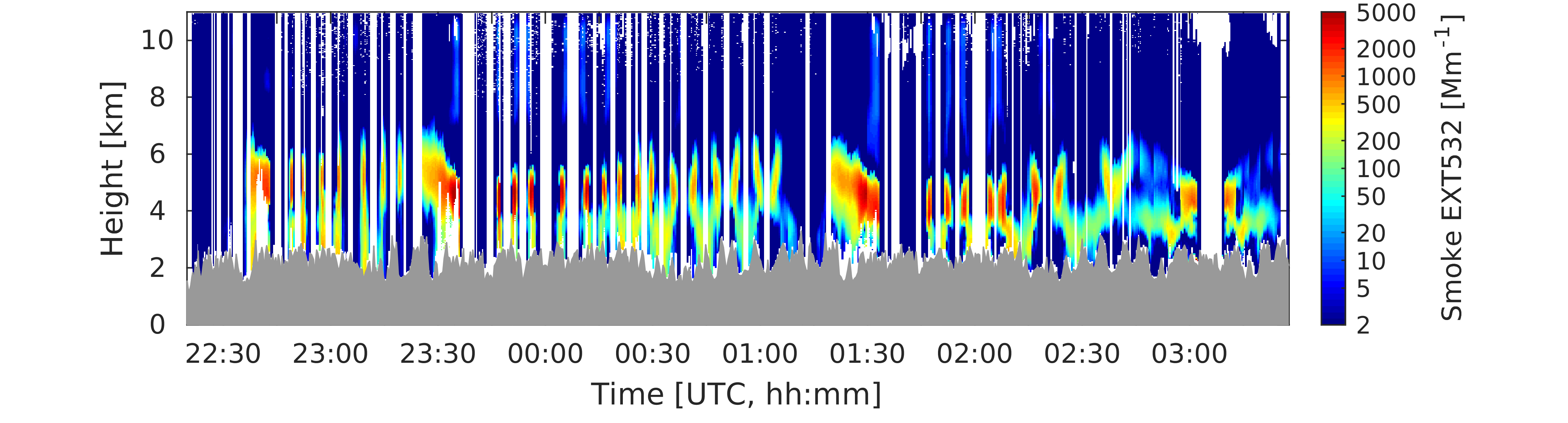


Obs

Base

Analysis

**Figure S9**. Curtain plots smoke aerosol extinction enhancement at 532 nm for Shady fire from 22:20 UTC 25 July to 03:28 UTC 26 July 2019. The three panels show DIAL-HSRL observations (top), simulation of the Base run (middle), and simulation with constrained emissions (bottom). The blank areas correspond to missing data. Areas with no data in either direction is when the lidar was turned off while in a turn of the aircraft. Missing data below or above the plume are due to the strong attenuation of the thick plume.


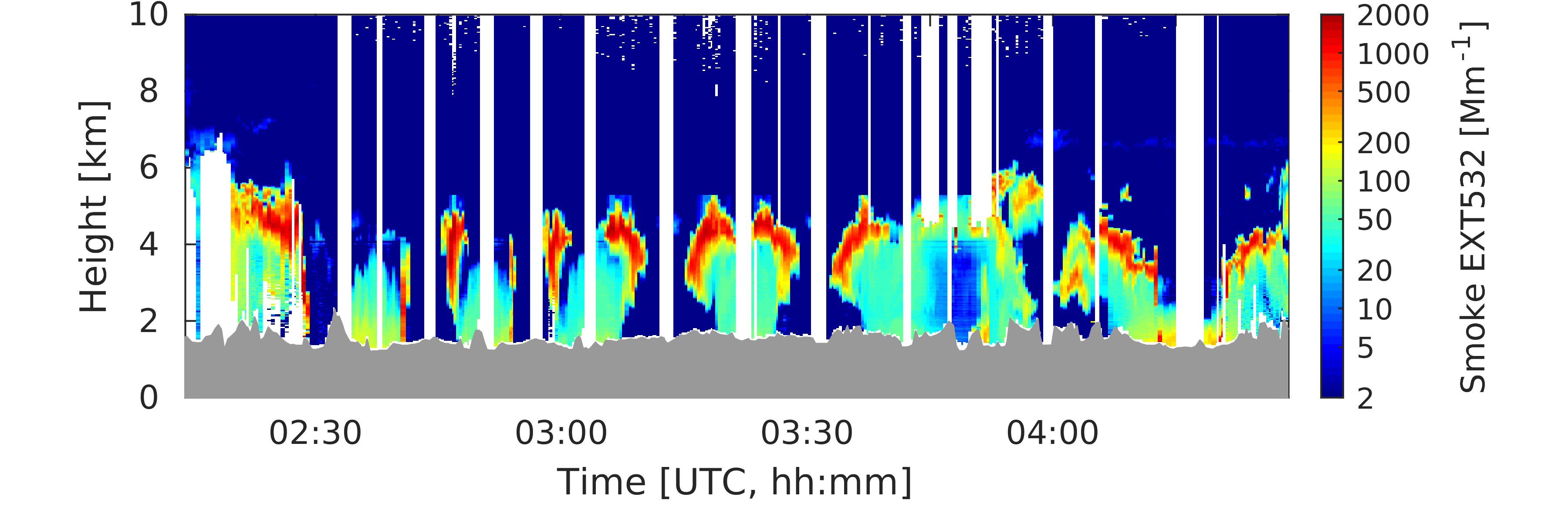

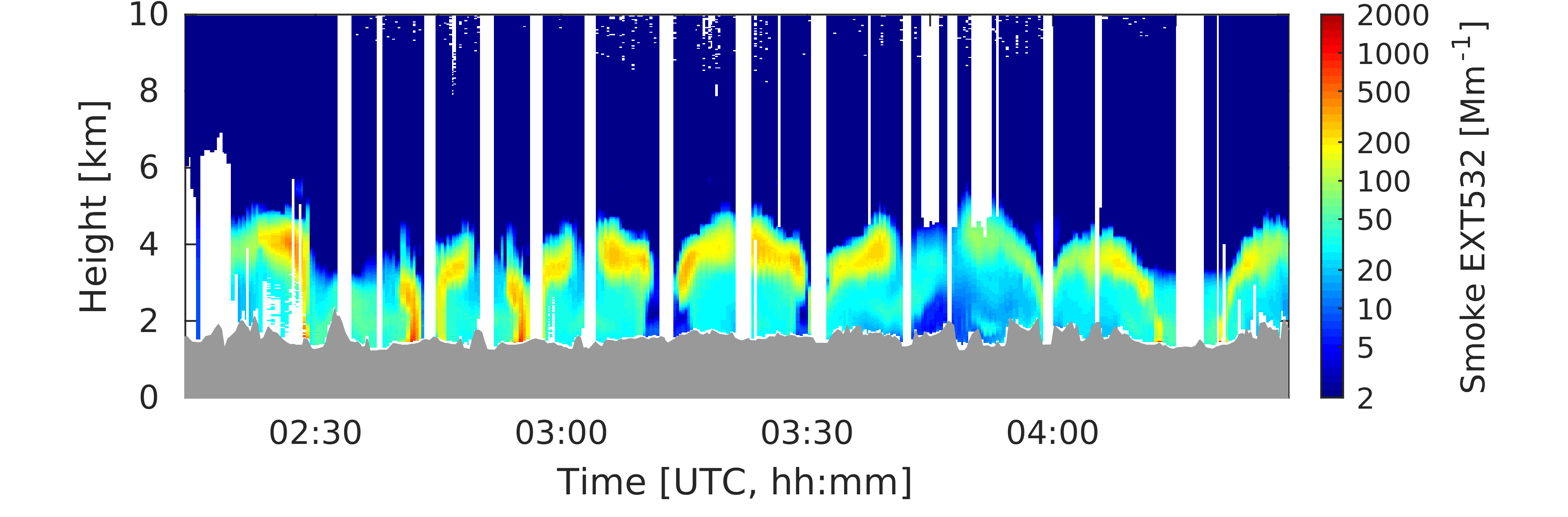

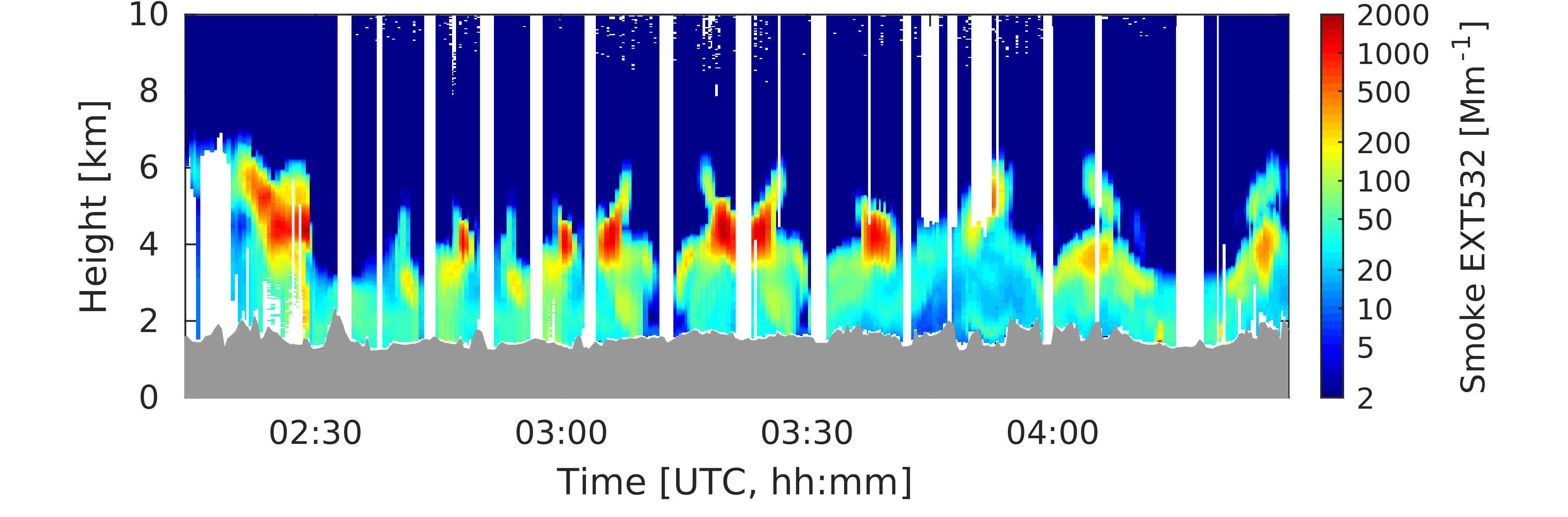


Obs

Base

Analysis

**Figure S10**. Similar to Fig. S9, but for Tucker fire showing results from 02:14 UTC to 04:29 UTC 30 July 2019.


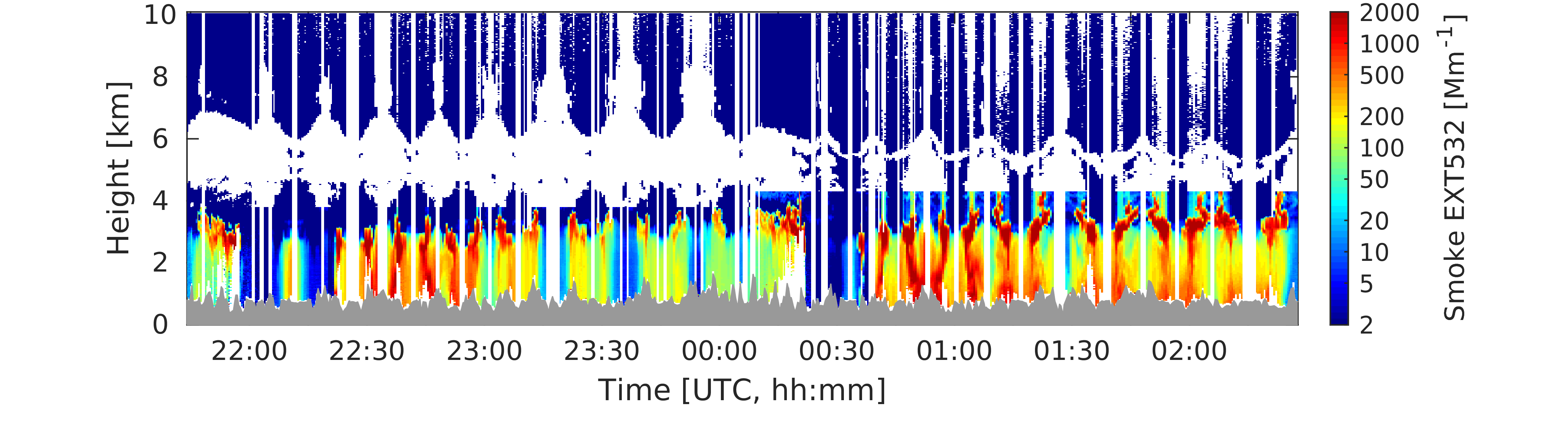

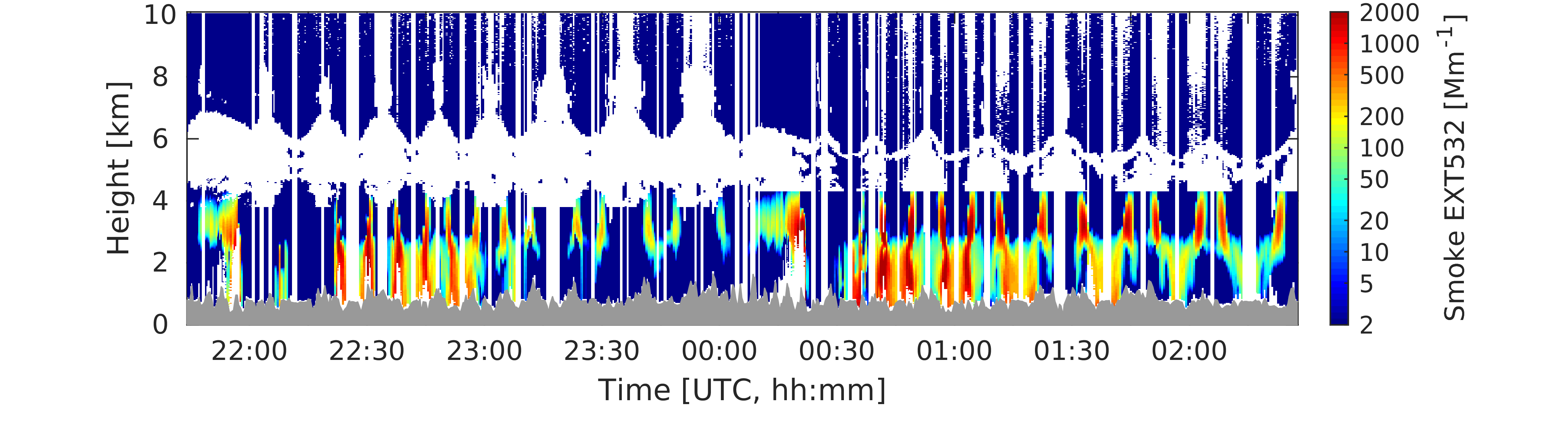

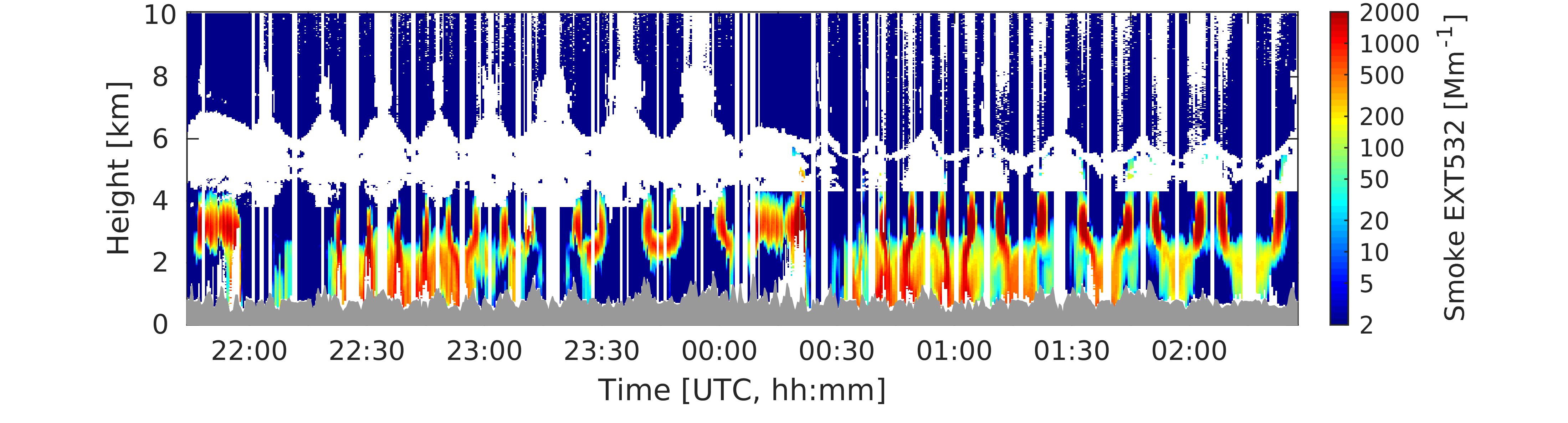


Obs

Base

Analysis

**Figure S11**. Similar to Fig. S9, but for Williams Flats fire showing results from 21:44 UTC 3 August to 02:28 4 August 2019. Note that the signals related to Siberian fires transported over this region have been filtered out, as represented by the blank areas between ~ 5 – 8 km.


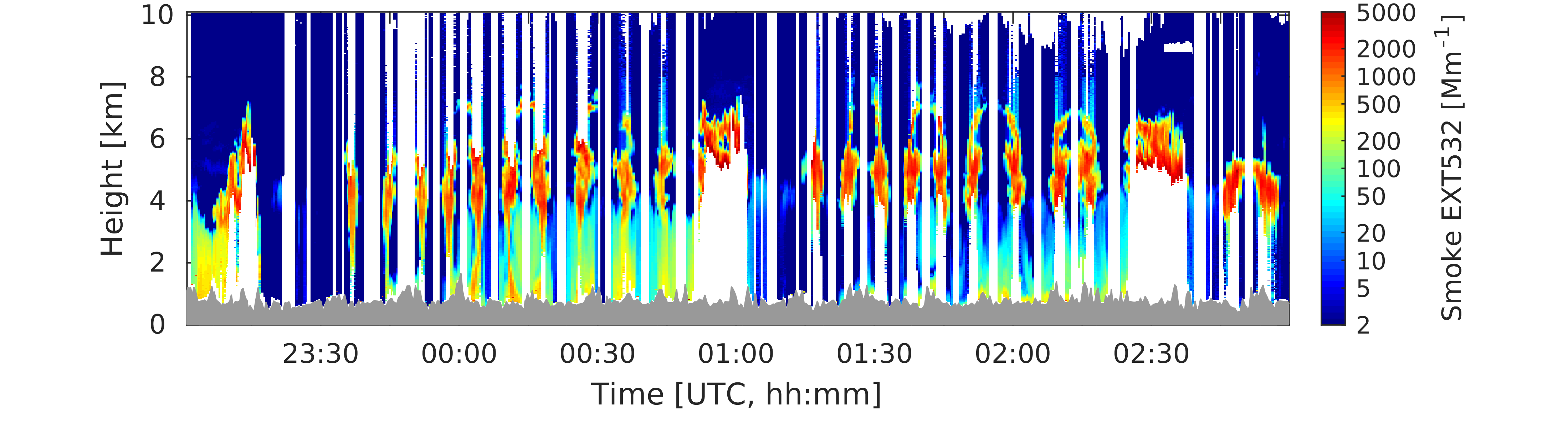

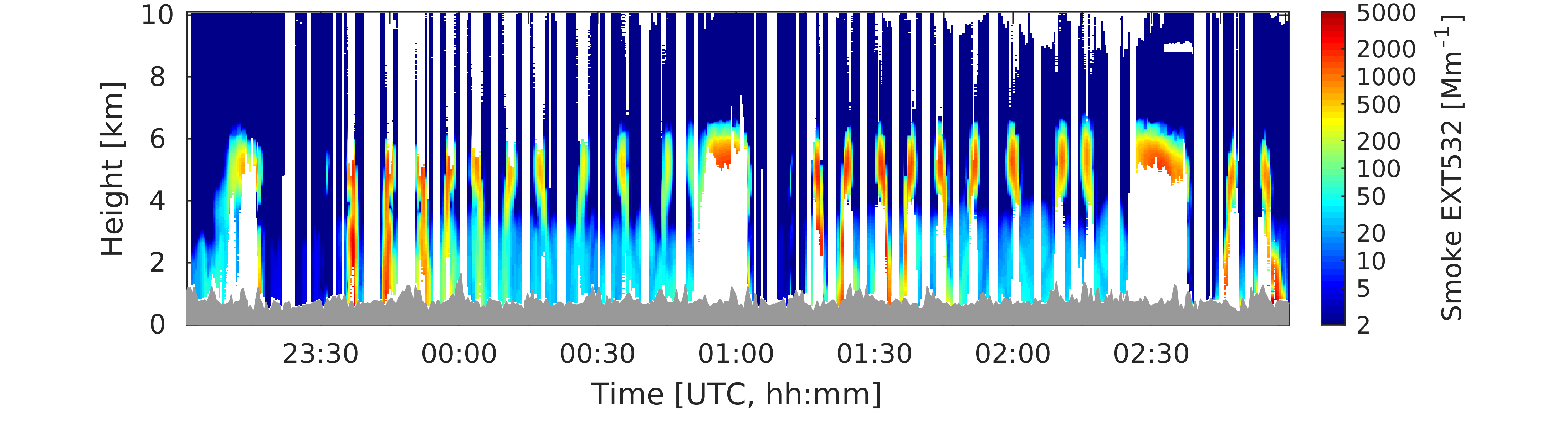

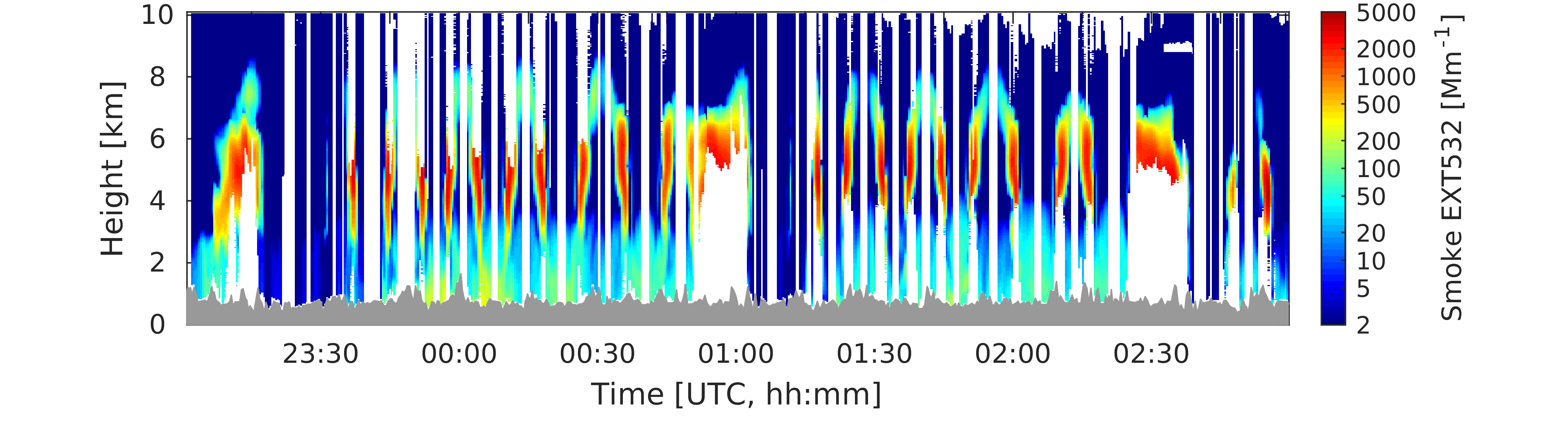


Obs

Base

Analysis

**Figure S12**. Similar to Fig. S9, but for Willimas Flats fire showing results from 23:01 UTC 7 August to 03:00 8 August 2019.


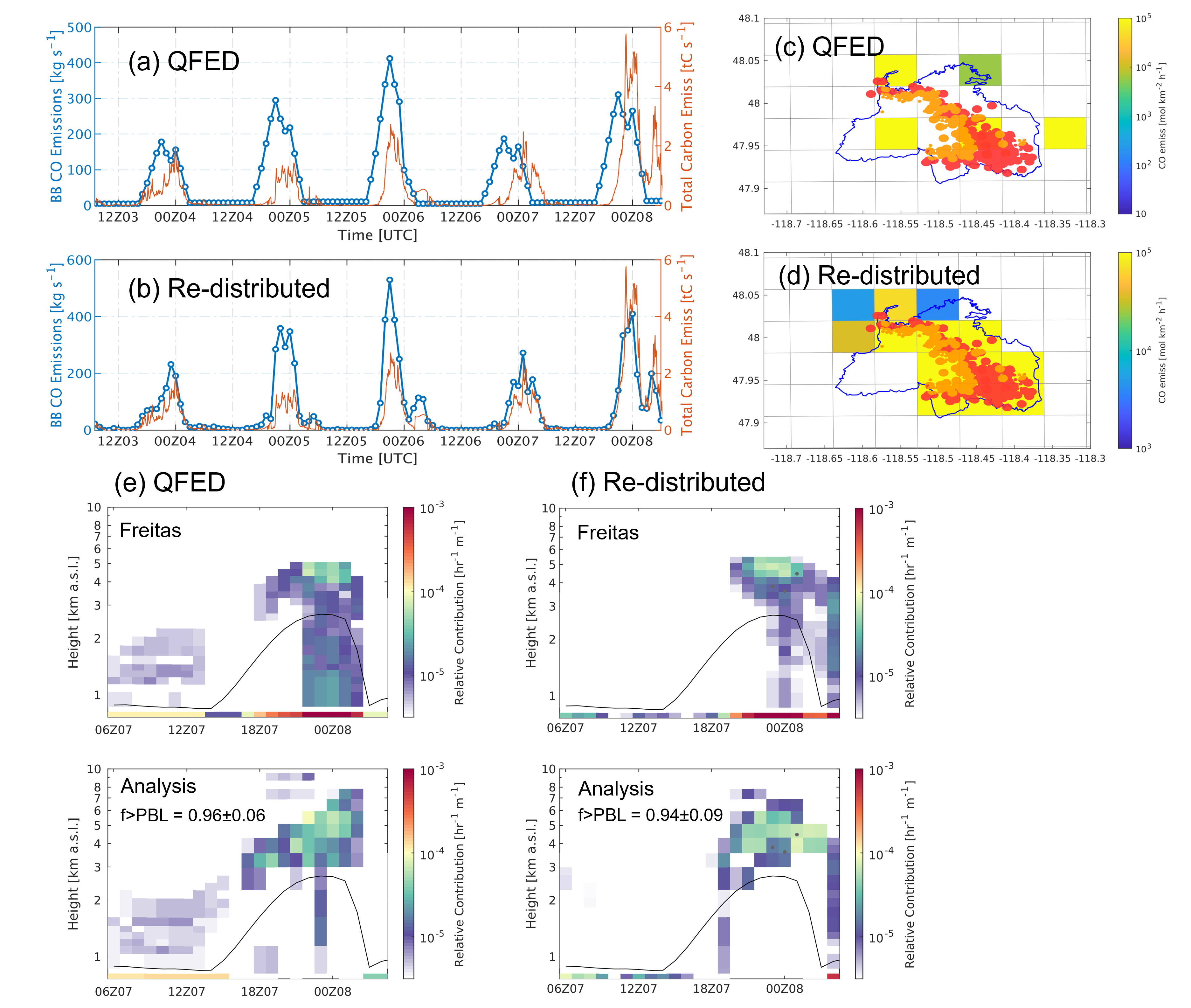


**Figure S13**. Sensitivity of the fire emission profile inversion result on the spatial and temporal distribution of the a priori emissions for Williamvs Flats fire on 7 August 2019. Two sets of emissions are tested: (1) QFED emissions processed with the emission processor “fire_emiss” developed by NCAR (named as “QFED”), and (2) re-distributed spatially and temporally following the Fuel2Fire data (“Re-distributed”). (a-b) Time series of CO emissions from the Williams Flats fire (left y-axis), compared to the total carbon emissions from Fuel2Fire data (right y-axis). (c-d) Spatial distribution of the daily total emissions. The red- and orange-colored circles stand for observed new and residual fire detection footprints, respectively, based on MODIS and VIIRS. (e) Relative distribution of fire emissions by time and height from the simulation using Freitas plume rise scheme (“Freitas”) and from the inversion using DIAL-HSRL data (“Analysis”). (f) Similar to (e), but for the re-distributed emissions.


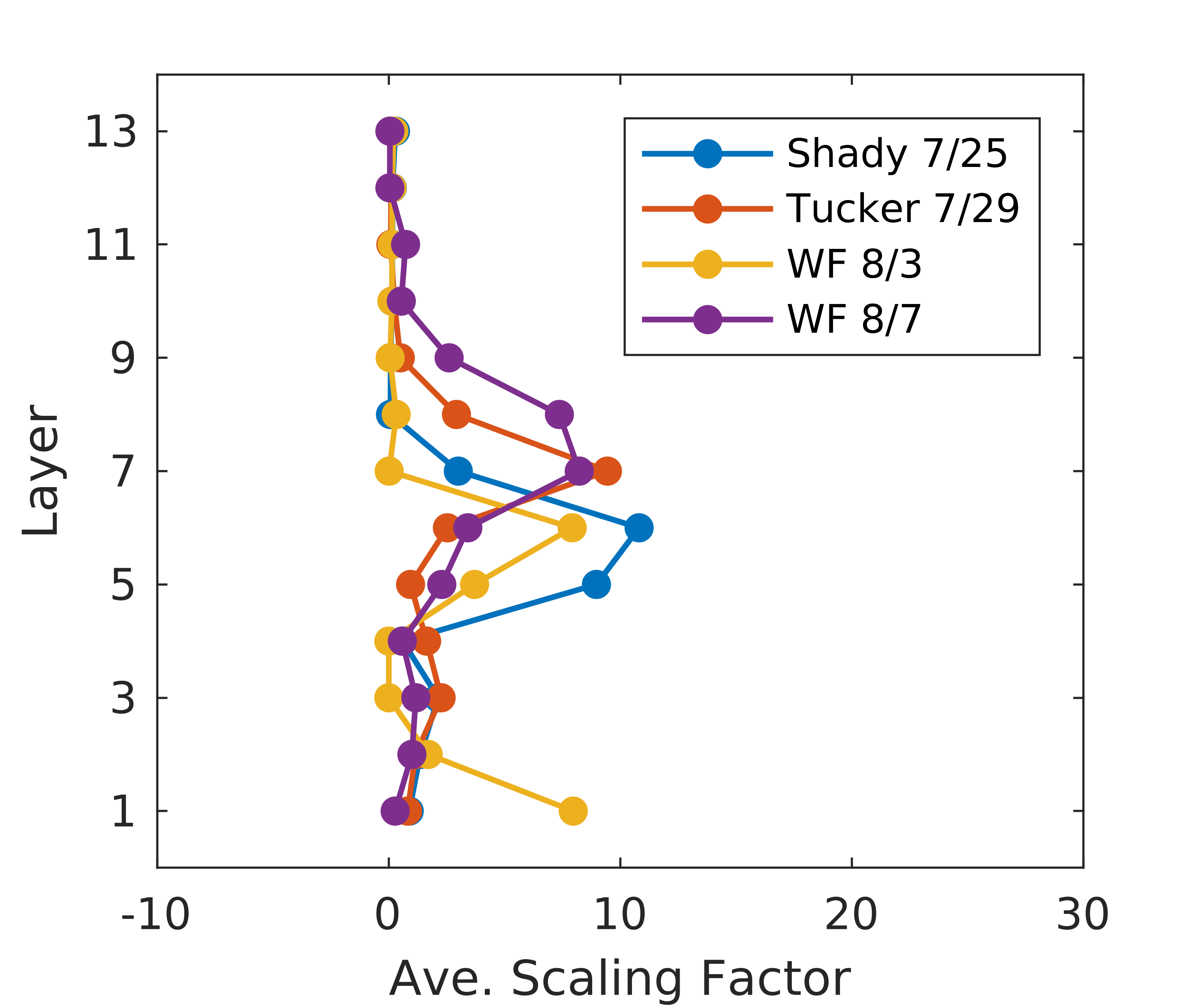


**Figure** **S14**. Average emissions scaling factors over the period of interest at different tracer layers for the four fire cases.


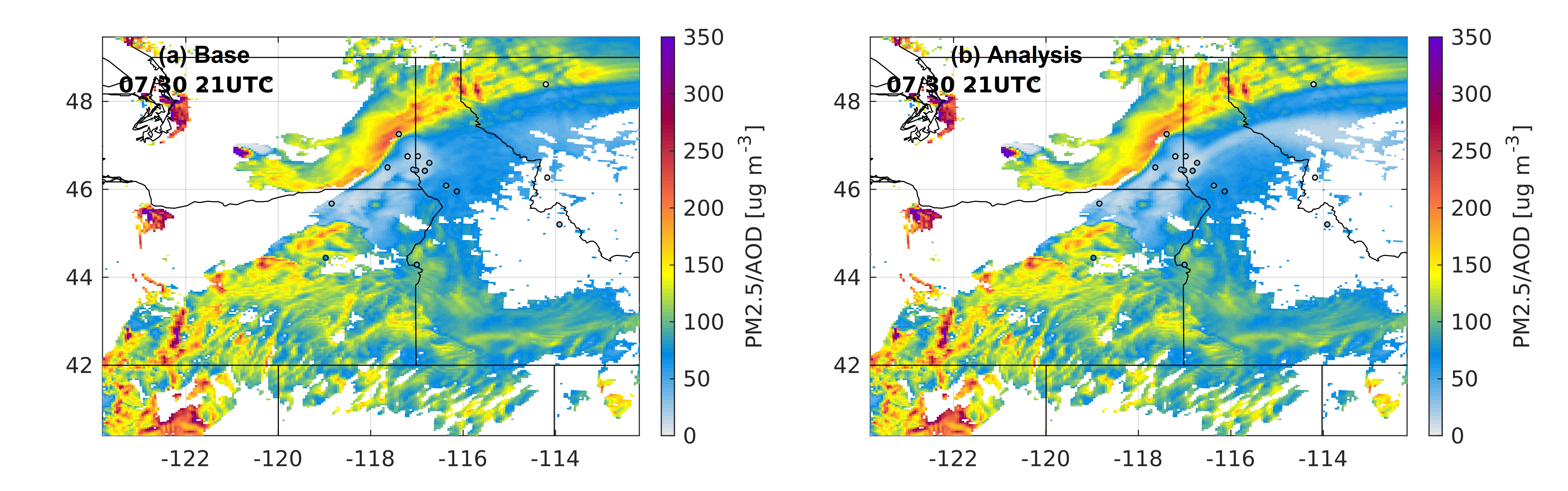


**Figure S15**. Modeled maps of PM2.5/AOD ratio for the (a) Base and (b) Analysis runs at 21 UTC on 30 July 2019. The observations of PM2.5/AOD are marked by the colored circles, calculated using MODIS MAIAC AOD and surface PM2.5 monitoring data. Note that to highlight the area impacted by smoke plume, filters of smoke AOD enhancements (sAOD) are applied to keep only the region with sAOD > 0.01 for model results and sAOD > 0.05 for observations.


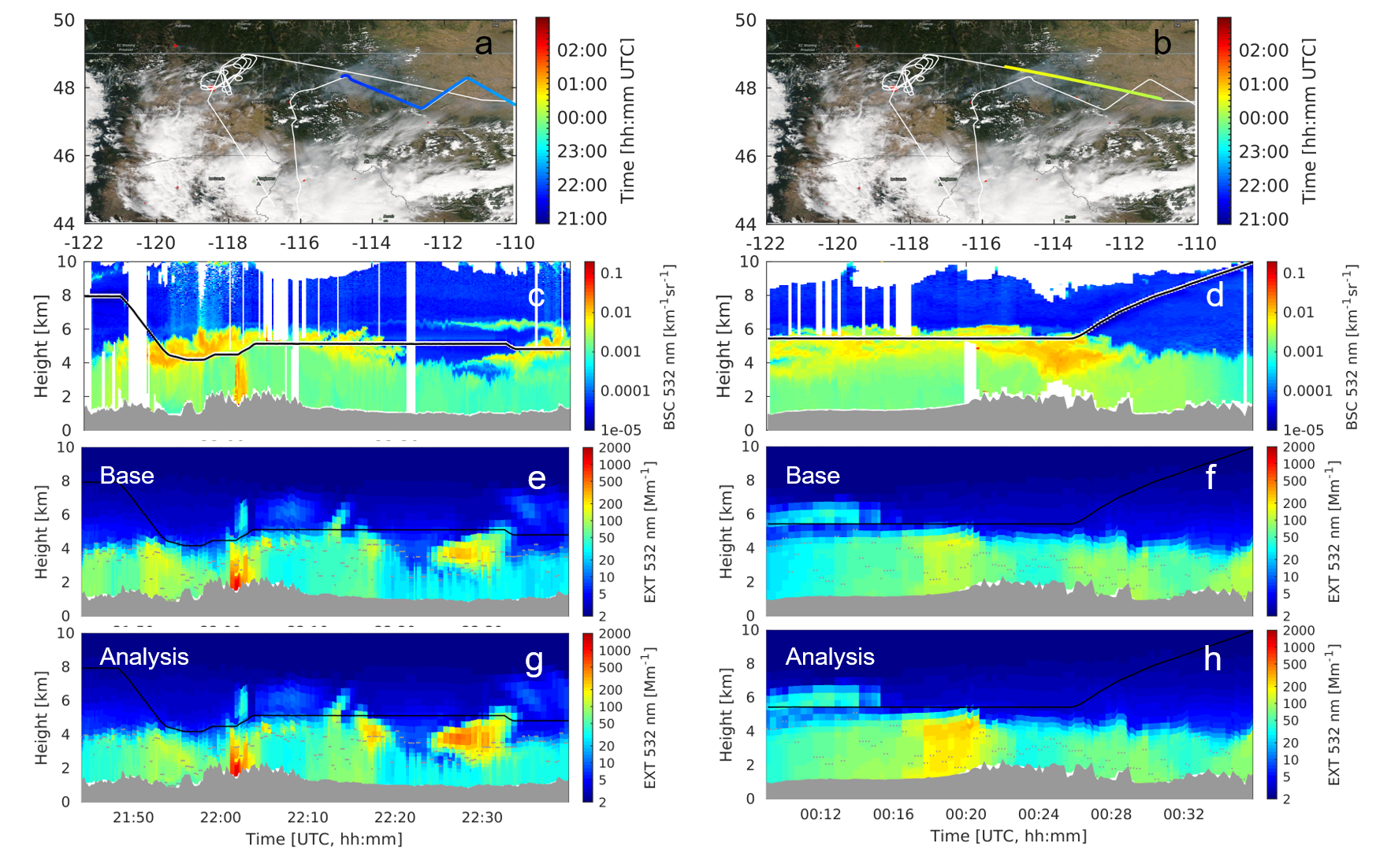


**Figure S16**. Comparison of modeled extinction profiles by the Base and Analysis runs (e-h) against DIAL-HSRL observations of aerosol backscatter (c-d). The left and right column show the results for two transects of 21:44 – 22:40 UTC, 8 August 2019 and 00:09 – 00:36 UTC, 9 August 2019, respectively. Location of the two selected flight transect are shown on maps (a-b), overlayed on top of Aqua-MODIS visible images obtained from NASA Worldview (<https://worldview.earthdata.nasa.gov/>). The black line in panels (c) to (h) represents flight height. Note that the strong extinction enhancements within the PBL in panels (c), (e), and (g) around 22:00 corresponds to another fire.


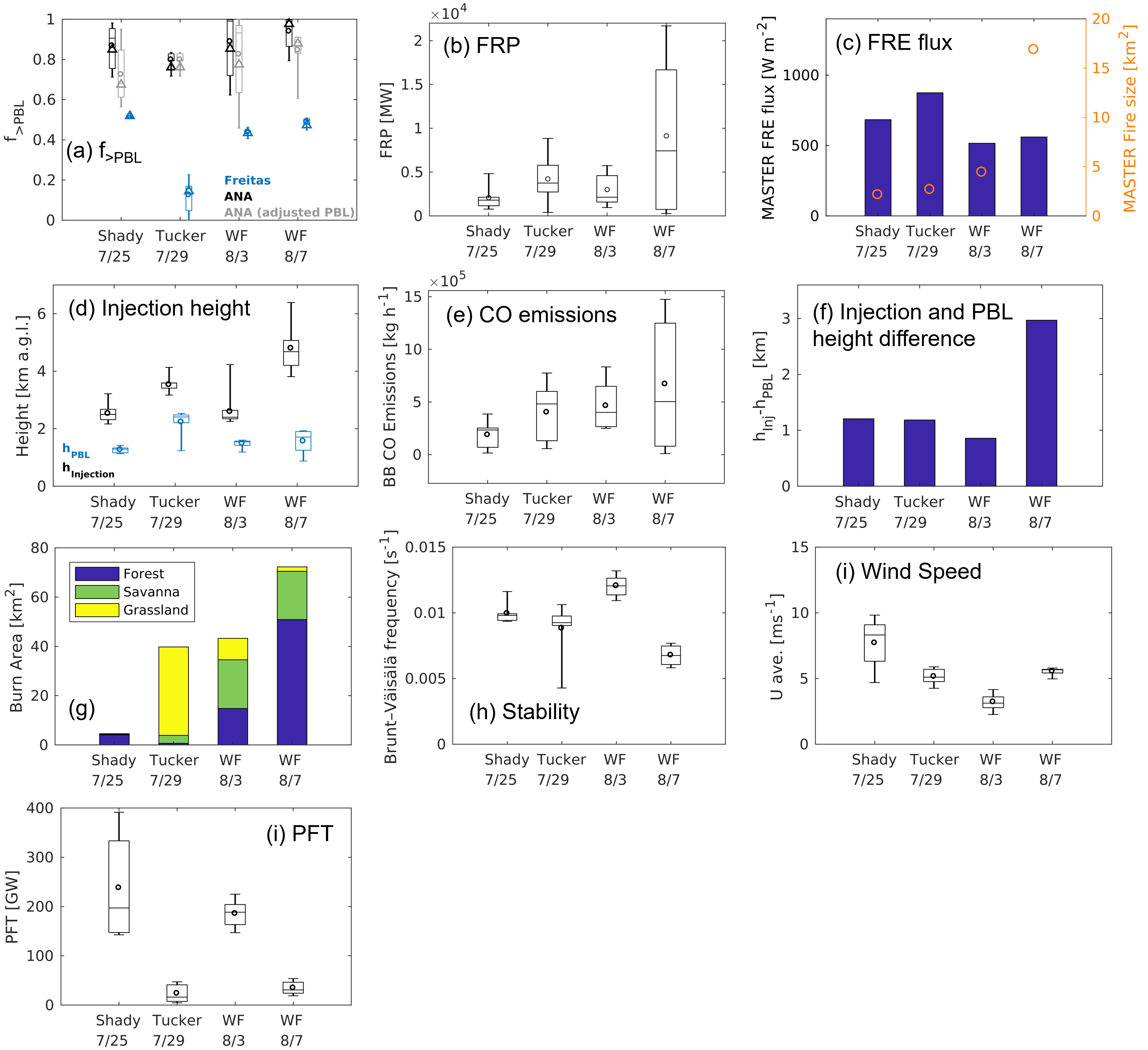


**Figure S17**. Comparison of free-troposphere smoke injection fraction (f>_PBL_) and other parameters of fire burning features and atmospheric conditions, as are labeled on each panel. (a) Free-troposphere smoke injected fraction (f_>PBL_) are shown for model estimates by the Freitas, constrained by the inverse modeling (Analysis), and Analysis with adjusted PBL height. (b-i) See text S2 for descriptions and derivation of the parameters. For the box-whisker plots, the edges and center line of the box stand for the 25^th^, 75^th^ percentiles and the median, respectively, the lower and upper whiskers extend to the minimum and maximum, and the circle stands for the average. The triangle (a) represents the value calculated for smoke emissions during the full period considered.


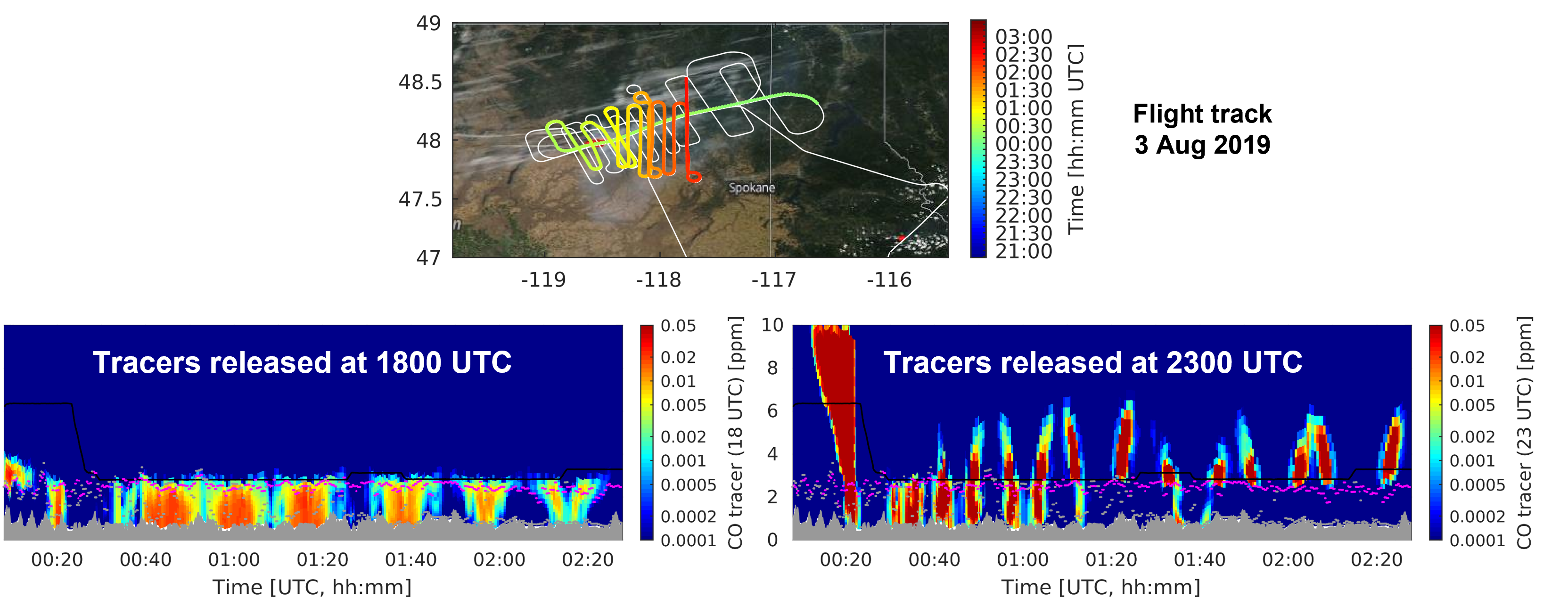


**Figure S18**. Smoke CO tracer concentration (units: ppm) along flight track subset sampling the Williams Flats fire on 3 August 2019. The CO tracers correspond to emissions released in the hour of 18:00 UTC and 23:00 UTC 3 August.


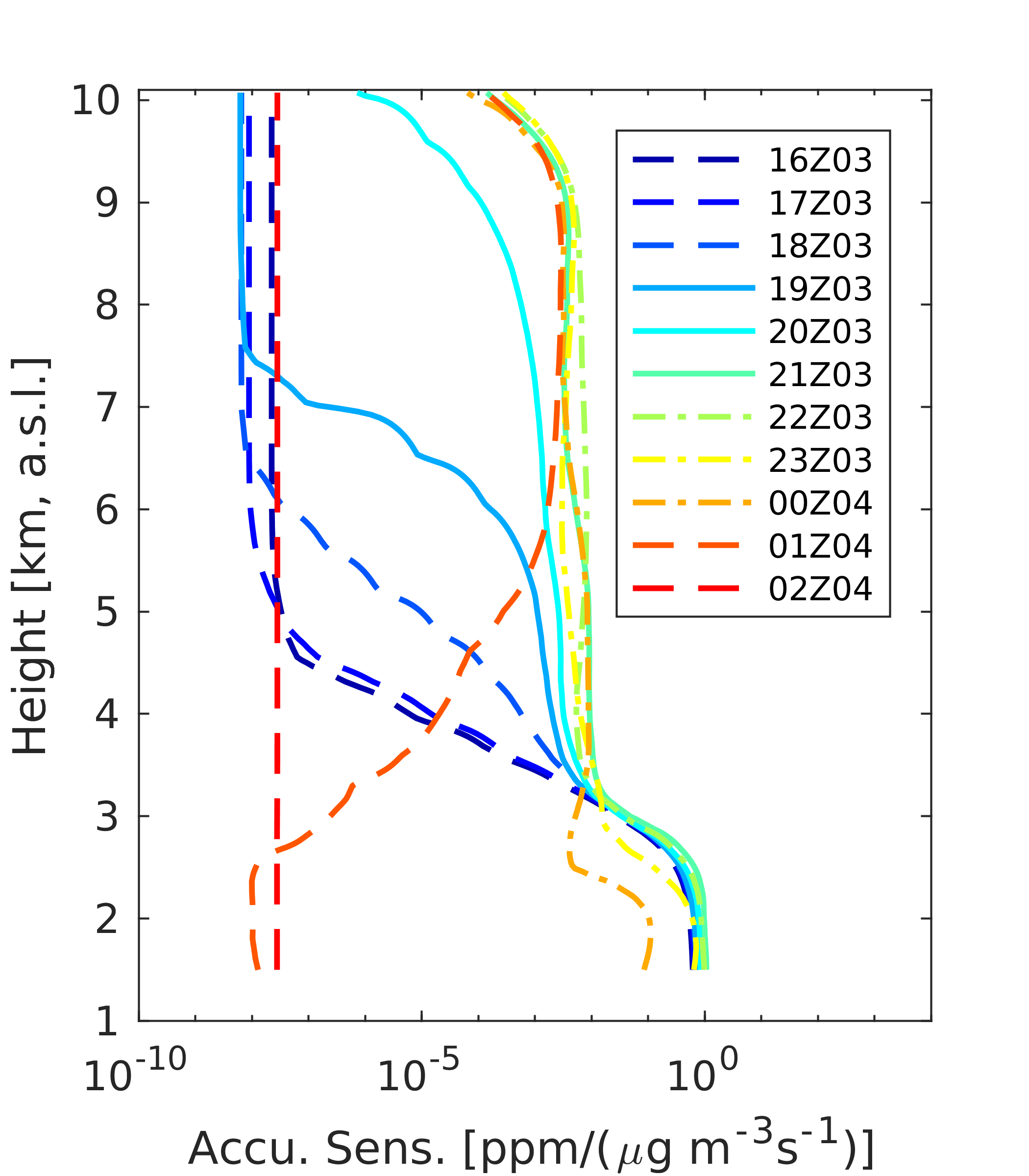


**Figure S19**. Accumulated sensitivity (units: ppm/(ug m-3 s-1)) along flight track shown in Fig. S18 sampling the Williams Flats fire. The sensitivity is calculated as the accumulated CO tracer concentrations per unit emission intensity.
